# An overview of reviews on strategies to reduce health inequalities

**DOI:** 10.1186/s12939-020-01299-w

**Published:** 2020-10-28

**Authors:** Nathaly Garzón-Orjuela, Daniel Felipe Samacá-Samacá, Silvia Catalina Luque Angulo, Carmen Verônica Mendes Abdala, Ludovic Reveiz, Javier Eslava-Schmalbach

**Affiliations:** 1 Grupo de Equidad en Salud, Facultad de Medicina, Universidad Nacional de Colombia, Bogotá, Colombia; 2 Centro Latinoamericano y del Caribe de Información en Ciencias de la Salud, BIREME/OPS/OMS, São Paulo, Brazil; 3 Evidence and Intelligence for Action in Health Department, Pan American Health Organization, Washington DC, USA; 4 Hospital Universitario Nacional de Colombia, Bogotá, Colombia

**Keywords:** Health equity, Strategies, Health status disparities, Systematic review

## Abstract

**Background:**

Governments are incentivized to develop and implement health action programs focused on equity to ensure progress with effective strategies or interventions.

**Objective:**

Identify and synthesize strategies or interventions that facilitate the reduction of health inequalities.

**Methods:**

A systematic search strategy was carried out up until August 2019 in MEDLINE (Ovid), Embase (Elsevier), Cochrane Database of Systematic Reviews, LILACS, Scopus, Scielo and Epistemonikos. In addition, a snowball strategy was used. Literature reviews (LRs) of experimental and quasi-experimental studies were included. The identified interventions and outcomes were categorized based on the recommendation by the Cochrane group in “*Effective Practice and Organization of Care*”. The quality of the included LRs was evaluated using the AMSTAR 2 tool.

**Results:**

Four thousand ninety-five articles were identified, of which 97 were included in the synthesis of evidence. Most of the studies included focused on the general population, vulnerable populations and minority populations. The subjects of general health and healthy lifestyles were the most commonly addressed. According to the classification of the type of intervention, the domain covered most was the delivery arrangements, followed by the domain of implementation strategies. The most frequent group of outcomes was the reported outcome in (clinical) patients, followed by social outcomes.

**Conclusion:**

The strategies that facilitate the reduction of health inequalities must be intersectoral and multidisciplinary in nature, including all sectors of the health system. It is essential to continue generating interventions focused on strengthening health systems in order to achieve adequate universal health coverage, with a process of comprehensive and quality care.

**Supplementary information:**

**Supplementary information** accompanies this paper at 10.1186/s12939-020-01299-w.

## Background

The reduction of health inequalities, described as the differences in health among people or society [1, 2], is a key issue on the global agenda [3]. The implementation of health policies and programs is intended to improve the health conditions of the population in aspects relevant to public policy, based on prioritized needs, and which must be resolved in the short, medium or long term. When implementing these health policies and programs, there is the possibility that the implementation will help to reduce inequalities in health (or at least not to increase them through implementation). Taking into account that equity is one of the objectives of sustainable development (OSD) [3], that it has been linked to the justice concept, redistribution of wealth and income, good governance, empowerment, and transparency [4]. Among those OSD, health inequities have been increasing in recent years [5] that means a climb in the differences in health that are avoidable, unjust and unneeded [1, 2]. Equity in health defined as the lack of health disparities that *“are systematic, potentially avoidable differences in health—or in the major socially determined influences on health—between groups of people who have different relative positions in social hierarchies according to wealth, power, or prestige”* [6]. Overall, this concept is becoming strategic, where possible, for the implementation of health policies and programs at a global level.

Decision makers are aware of the need to reduce health inequalities [7]. However, putting it into practice is not easy due to the high amount of information evidenced in the literature that making the process of choice difficult for decision-makers [8]. Barsanti et al. [9] state that despite the priority of governments to reduce health inequalities, clear objectives are often lacking, as well as impact evaluation systems to demonstrate the effectiveness of actions and interventions.

Currently, governments are incentivized to develop and implement health action programs focused on equity to ensure progress with effective strategies, where identify actions on social determinants of health through interventions in the health, economic and education sectors, by mean of designing a plan to resources to set priorities and crafting solutions, dedicating time, and political attention [10]. For this reason, actions such as the identification, synthesis and transfer of scientific knowledge are required, a process that requires transparent and reproducible research methods that allow evidence to be evaluated more efficiently [9]. Also, some authors have develop overview of reviews focused on strategies to implement evidence-based interventions in low-income countries [11] or to implement and evaluation of health promotion services and programs to improve cultural competency [12]. As a result, the objective of this overview of reviews was to identify and synthesize the strategies or interventions that facilitate the reduction of health inequalities.

## Methods

### Criteria for considering studies for this overview of literature reviews

#### Inclusion criteria

Literature reviews (LRs) of experimental and quasi-experimental studies, in English and Spanish, evaluating strategies or interventions focused on reducing health inequalities or inequities. It was restricted to publication date within the last 5 years (January 2014 to July 2019), owing to the amount of information provided in the literature and the recommendation by the Cochrane group on the maintenance and periodic updating of LRs [13].

#### Exclusion criteria

LRs with unclear methodology specifically the search method for identification of information (i.e. narrative review).

### Search method for identification of information

A systematic search strategy was performed in the following electronic databases: MEDLINE (Ovid), Embase (Elsevier), Cochrane Database of Systematic Reviews - CDSR (Wiley platform), LILACS (Virtual Health Library - VHL), Scopus, Scielo and Epistemonikos. The search strategy was composed of key concepts related to “*health education*”, “*health planning*”, “*training*”, “*healthcare disparities*”, “*health inequities*”, “*health inequalities*”, and other terms that can see in Additional file 1. In addition, a manual “snowball” search was performed by reviewing the list of bibliographic references of the selected studies and Google Scholar.

### Collection and synthesis of information

An initial review by title and summary of potentially eligible studies was performed by three of the authors. Subsequently the full text of the preselected studies was revised for its final inclusion. A data extraction form was designed in which including information of the included studies relating to types of studies included in the review, type of population, intervention, comparator, health outcomes, reduction of health inequalities and main conclusions. The data was synthesized narratively. The interventions identified were categorized based on the EPOC taxonomy (*Effective Practice and Organization of Care*) developed by the Cochrane EPOC group, which consists of classifying health system interventions into four domains of intervention (Table 1) with their respective subdomains and categories, which provide a structure for the classification of evidence regarding interventions or strategies to reduce health inequalities [14]. Likewise, the outcomes evidenced were classified into the categories recommended by the Cochrane EPOC group [15].

**Table 1:** Defining the domains of the types of interventions

Domains	Definition
Delivery arrangements	Interventions aimed at generating changes in how, when and where health care is organized, as well as who provides care services.
Financial arrangements	Interventions aimed at seeking changes in how insurance funds and plans are raised, how services are purchased, and the use of financial incentives or disincentives.
Governance arrangements	Interventions related to rules or processes that affect the way powers are exercised, particularly with respect to authority, responsibility, openness, participation and coherence.
Implementation strategies	Interventions designed to provoke changes in health care organizations, as well as the behavior of health professionals or the use of health services by users.

Source: Effective Practice and Organisation of Care (EPOC) Taxonomy. Cochrane Effective Practice and Organisation of Care. 2015 [14]

Additionally, to provide an overview of the evidence on health inequalities, a graphic synthesis was generated using the method adapted by BIREME in the “*International Initiative for Impact Evaluation (3ie -* https://www.3ieimpact.org)”. As a result, an evidence map, and an interactive online platform were obtained that allow users to explore the evidence base. In the evidence map, the bubbles are the intersections between the interventions and the findings, which denote the existence of at least one review (the larger the bubble, the greater the volume of evidence). The color of each bubble represents the type of evidence and a confidence rating. In the online version, hovering over a bubble displays a list of the evidence for that cell. The links for these studies lead to registration in a database of the Virtual Health Library (VHL). Users can filter the evidence by type, confidence rating, region, country, study design, and population. To generalizing and standardization of type of population of each review included and added in the evidence map, it was based on the eligibility criteria original of each review if it was not clear, the final decision was done through a consensus among the authors after evaluating the full-text.

### Quality assessment

The quality of the LR included for the synthesis of evidence was evaluated using the AMSTAR 2 tool for reviews that included randomized or non-randomized studies of health interventions [16]. Due to the evidence map includes the type of evidence and a confidence rating, the overall score result of AMSTAR 2 was classified in high, moderate, and low quality with the online “*AMSTAR stands for A MeaSurement Tool to Assess systematic Reviews*” [17].

## Results

**Fig. 1:**
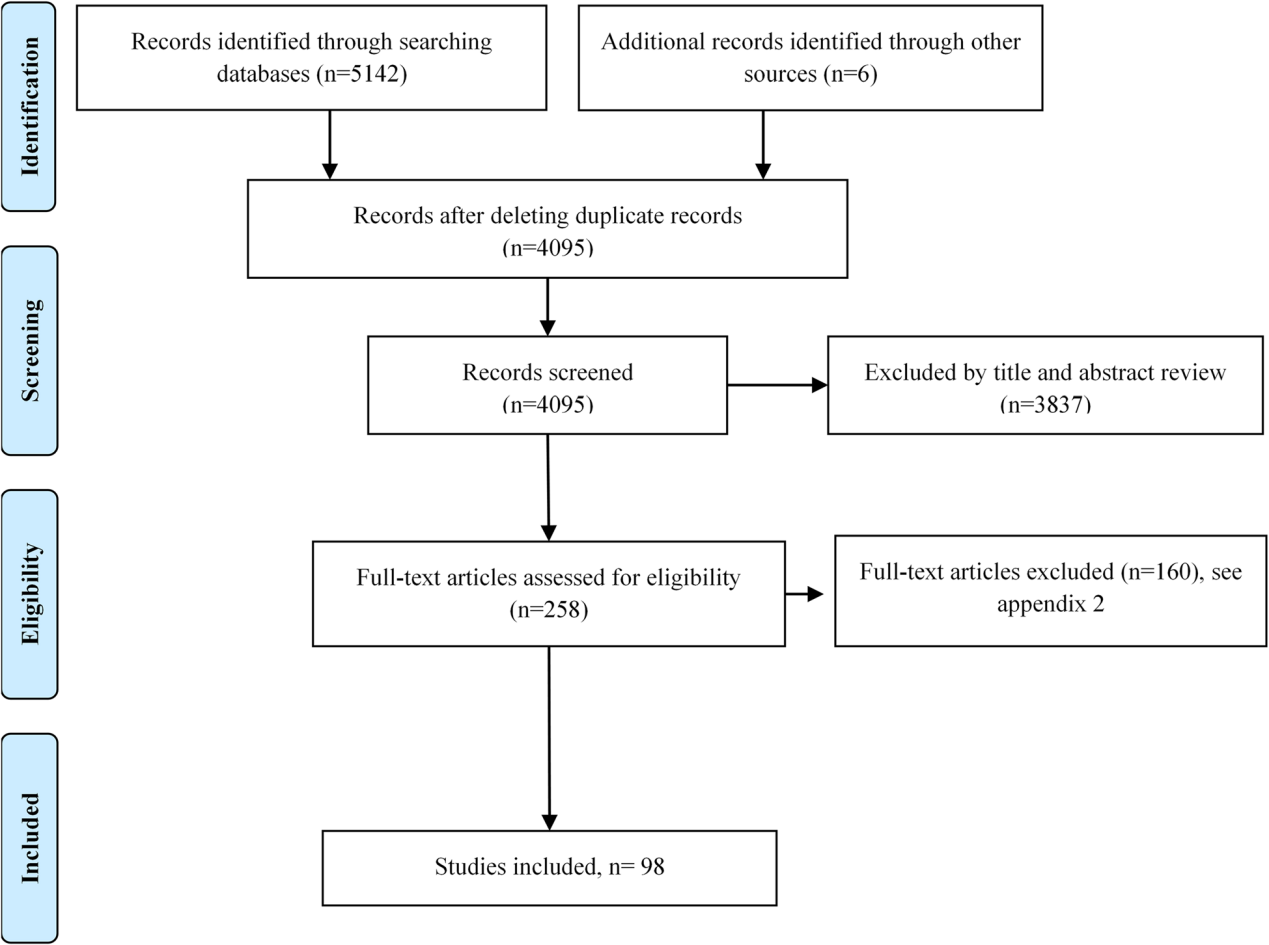
Flow diagram (PRISMA)

After removing duplicates, a total of 4095 references were identified. After reviewing the full text, 98 studies were included in the synthesis of evidence (Fig. 1). Additional file 2 shows the list of excluded studies. The characteristics of the included studies and the overall result of the quality assessment are detailed in Additional file 3.

### Evidence map

**Fig. 2:**
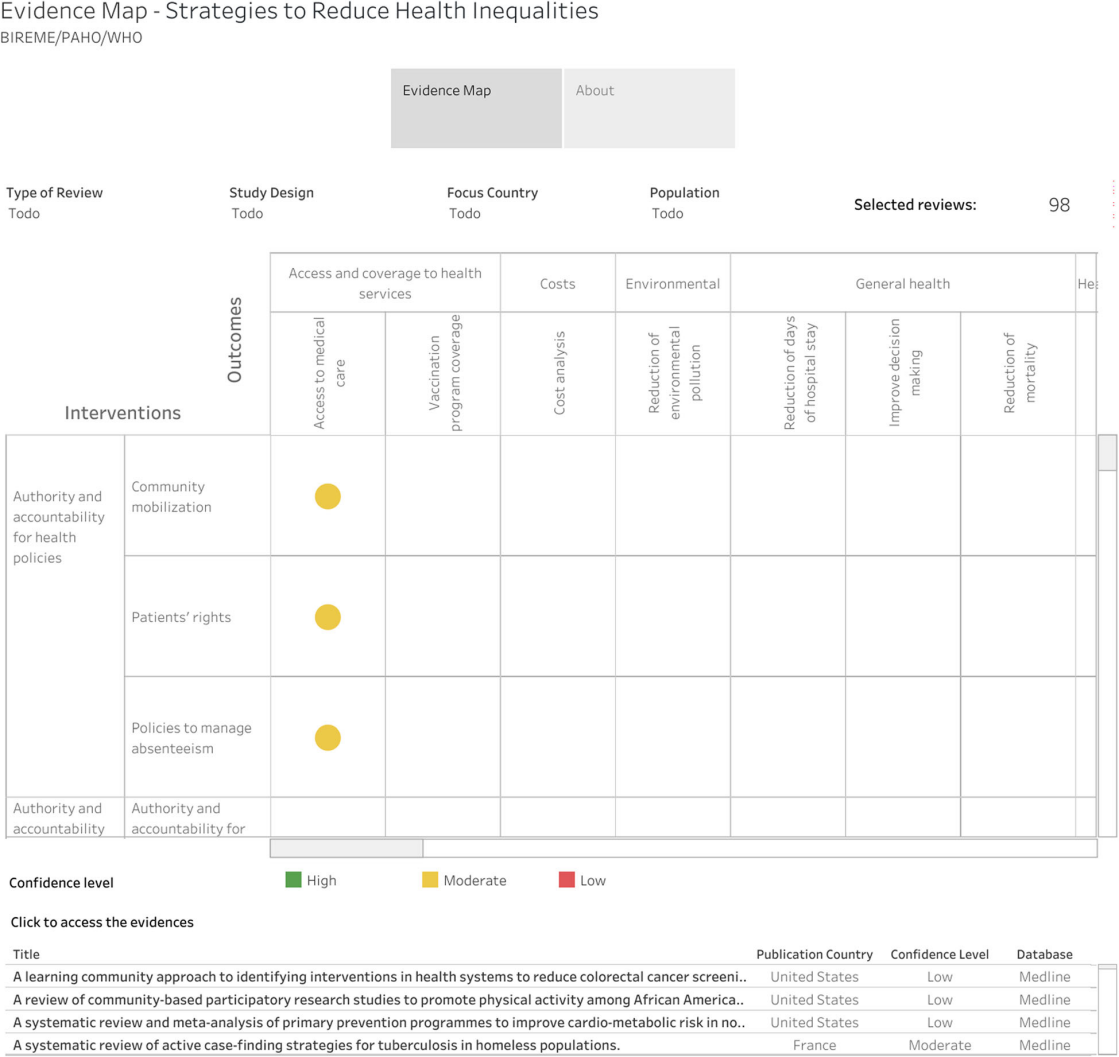
Evidence map. Source: BIREME, based on the characterization of each literature review (LR) included

From the characterization of the included studies, an evidence map was developed, which graphically distributes these studies according to a matrix with 38 interventions and 39 findings that can be observed in Fig. 2 and at the following link: https://public.tableau.com/profile/bireme#!/vizhome/desigualdades-en-salud-en/evidence-map. In this evidence map, it is shown that 10 of the included RSs displayed a high level of confidence, including outcomes of access to medical care, costs, health care, reduction of inequalities, promotion and prevention, among others.

Most of the included studies focused on the general population (including any age group, or focused on the community in general), vulnerable populations (marginalized groups and sex workers, among others) and minority populations (ethnic or racial group). The topics of general health (26.5%) and healthy lifestyles (16.3%) were the most addressed by the included LRs. Additionally, 63.3% of the strategies evidenced in the LR were focused on the health sector and 36.7% were of an intersectoral nature (Additional file 3).

According to the classification of the type of intervention by the EPOC taxonomy [14], the most covered domain in LRs was the domain of service provision, followed by the domain of implementation strategies (Table 2).

**Table 2:** Classification of the interventions identified in the included LRs

Types of intervention	n (%)^a^
- Delivery arrangements (domain)	77 (78.6)
• Coordination of care and management of care processes (category)	38 (49.4)
▪ Care pathways (subcategory)	26 (113)
▪ Integration the provision of different healthcare services (subcategory)	9 (39.1)
▪ Disease management (subcategory)	5 (21.7)
▪ Case management (subcategory)	3 (13)
▪ Communication between providers (subcategory)	3 (13)
▪ Continuity of care (subcategory)	2 (8.7)
▪ Multidisciplinary team of healthcare workers (subcategory)	1 (4.3)
• Who provides care and how the health care workforce is managed (category)	33 (42.9)
▪ Self-management (subcategory)	17 (65.4)
▪ Role expansion or task shifting (subcategory)	16 (61.5)
• Information and communication technology (category)	16 (20.8)
▪ Technology based methods to transfer healthcare information and support the delivery of care (subcategory)	11 (100)
▪ Health information systems (subcategory)	3 (27.3)
▪ Telemedicine (subcategory)	3 (27.3)
▪ Smart home technologies (subcategory)	1 (9.1)
• Where care is provided and changes in the healthcare environment (category)	16 (20.8)
▪ Site of service delivery (subcategory)	10 (83.3)
▪ Changes to the physical or sensory healthcare environment, by adding or altering equipment (subcategory)	3 (25)
▪ Visits by health workers to different locations (subcategory)	2 (16.7)
▪ Arrangements for transporting patients from one place to another (subcategory)	1 (8.3)
• How and when care is provided (category)	7 (9.1)
▪ Quality and safety systems (subcategory)	3 (50)
▪ Coordination of care among different providers (subcategory)	2 (33.3)
▪ Group versus individual care (subcategory)	1 (16.7)
▪ A reduction or increase in time to access a healthcare intervention (subcategory)	1 (16.7)
- Financial arrangements (domain)	7 (6.1)
• Mechanisms for payment of health services (category)	6 (85.7)
▪ Voucher schemes (subcategory)	6 (100)
• Insurance schemes (category)	1 (16.7)
▪ Community-based health insurance (subcategory)	1 (100)
- Governance arrangements (domain)	7 (7.1)
• Authority and accountability for health policies (category)	6 (85.7)
▪ Community mobilization (subcategory)	3 (50)
▪ Patients’ rights (subcategory)	2 (33.3)
▪ Policies to manage absenteeism (subcategory)	2 (33.3)
• Authority and accountability for health professionals (category)	1 (14.3)
▪ Authority and accountability for the quality of the practice (subcategory)	1 (100)
- Implementation strategies (domain)	62 (63.3)
• Interventions targeted at specific types of practice, conditions or settings (category)	45 (72.6)
▪ Practice and setting (subcategory)	36 (100)
▪ Health conditions (subcategory)	11 (30.6)
• Interventions targeted at healthcare workers (category)	28 (45.2)
▪ Communities of practice (subcategory)	9 (34.6)
▪ Tailored interventions (subcategory)	8 (30.8)
▪ Patient-mediated interventions (subcategory)	4 (15.4)
▪ Educational meetings (subcategory)	3 (11.5)
▪ Educational outreach visits, or academic detailing (subcategory)	3 (11.5)
▪ Adherence of clinical practice guidelines (subcategory)	2 (7.7)
▪ Routine patient-reported outcome measures (subcategory)	2 (7.7)
▪ Inter-professional education (subcategory)	2 (7.7)
▪ Educational materials (subcategory)	1 (3.8)

Source: authors based on the information extracted from the literature reviews (LRs) included

^a^ n = total of domains, categories or subcategories contained in the interventions identified in the LR. Percentage = n / total of LR

### Delivery arrangements intervention

Of the total of LRs that included the delivery arrangements domain, eight displayed a high quality [18–25]. The two most frequent categories in this domain were “coordination of care and management of care processes”, and “who provides care and how the health care workforce is managed”. In addition, the most common subcategories were care pathways and self-management (Table 2).

In the case of self-management or self-care, the main focus was on interventions related to weight loss and the promotion of physical activity [26, 27], through interventions focused on lifestyle changes and behavioral counseling that have shown positive results in weight loss and diet modification [28, 29]. Likewise, it was evident in vulnerable populations that multi-component and personally tailored interventions based on counseling are more effective for glycemic control and the reduction of inequalities in oral health [30, 31].

In addition, the interventions of expansion or change of roles/tasks in care included the creation of medical-legal associations for the continuous search for patient well-being [32], community-based peer support where improvements in health literacy were observed for vulnerable populations [33], and nutritional interventions promoting healthy lifestyles aimed at indigenous populations, in which the community was encouraged to be involved in the design, implementation, and evaluation of interventions for their own well-being [34–36].

Regarding the category of the use of information and communication technologies, reviews were found showing positive results specifically in the reduction of body mass index in the obese population, through the use of text messages or calls [37], as well as computerized cognitive-behavioral interventions for the management of anxiety and/or depression for people living in rural and remote areas [38]. Similarly, it was shown that the implementation of interventions using audiovisual media, such as videoconferences and telemedicine, contribute to the health care of older adults since they help informed decision-making [18, 39]. It was also shown that this category of intervention had a positive effect on vulnerable populations with the use of educational strategies at home, telemedicine, text message reminders, calls and other technological contributions that facilitate access to health by improving the quality of life of patients and their caregivers [40–42].

Regarding the category of “where care is provided and changes in the healthcare environment,” several reviews included interventions that focused on improving access to healthcare services by connecting healthcare professionals to work or educational institutions, or directly to the homes of vulnerable populations with geographic difficulties or who had been displaced, to provide access health care [43–45]. In this context, subjects such as healthy eating in the school environment in low-income countries [36], assessment and rapid testing for the human immunodeficiency virus in street dwellers or disadvantaged women [19, 46], medical visits for detection of colorectal, breast and cervical cancer [47–51], and informed advocacy measures in the community to promote cardiovascular disease protective factors [27, 52] had positive effects when implemented in places where there are concentrations of disadvantaged groups, thus improving access to health care.

### Financial arrangements intervention

The six LRs that included the financial arrangements domain were of an average quality [26, 43, 50, 53–55]. The category of “mechanisms for the payment of health services” was the most common within this domain (Table 2), which focused on providing financial support to disadvantaged groups to guarantee health care – for example, transportation aid (paying for transport tickets or reimbursement of tickets) to socioeconomically disadvantaged groups, improving access to the different care services [53]. Interventions with economic incentives accompanied by other types of interventions were also found, aiming to provide a comprehensive package such as self-care interventions and technology management, which improve access to diagnostic cancer detection procedures [26] or tuberculosis [43].

### Governance arrangements intervention

Governance arrangements domain was identified in seven of the included studies, one of high quality [25] and three of medium quality [53, 56, 57]. Interventions aimed at public policy in health or organizations were identified, where the construction of social networks and organizational interventions carried out in work environments improve health conditions, decrease hours and work stress, as well as health inequalities [25, 53].

### Implementation strategies

Sixty two LRs were included in the implementation strategies domain, of which six were of high quality [18, 22–24, 58, 59]. The category of interventions targeting specific practices and conditions was the most frequent (Table 2). Among these, effective strategies were identified in oral health care in the immigrant population [60] and the reduction of infant mortality in low- and middle-income countries [45]. Some programs aimed at specific conditions such as unemployment and its impact on health issues, such as reemployment and rapid job search, showed positive effects on outcomes such as quality of life [61].

These interventions were mainly aimed at indigenous, immigrant, maternal and child populations, and people of low socioeconomic levels. In addition, it was shown that in the population of pregnant women, multifaceted strategies are effective to improve prenatal controls, assisted childbirth and breastfeeding, but conditioned to the characteristics of each population [23, 62].

Regarding interventions aimed at health workers, it was noted that strategies focused on improving adherence to clinical practice guidelines lead to better results in primary care, specifically in the reduction of cardiovascular disease risk factors [52]. Likewise, the involvement of community participants as health agents in the implementation of the strategies shows reductions in maternal and infant mortality in low and middle income countries [63], in addition to improving access and coverage to health services in vulnerable populations with chronic diseases [34, 64].

In addition, three of the included studies evaluated the impact of “tailored” implementation strategies and health policies on equity. The interventions were aimed at tobacco control in adults, adolescents, and the general population [65–67]. In general, the increase in tobacco taxes improved equity both at the population level and at the individual level [65–67]. Likewise, it was found that interventions tend to be more effective at high economic levels, so a special approach is required according to social class and vulnerable population to avoid increasing the gap in health inequalities [65–67].

**Fig. 3:**
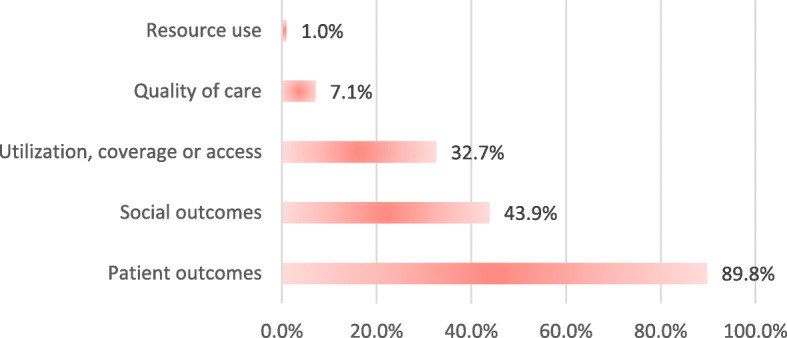
Classification of the outcomes identified in the literature reviews. Source: authors, based on the information extracted from the literature reviews (LRs) included Percentage = sum of each category of outcome contained in the interventions identified in the LR/total LR

Regarding the classification of the results, the outcome of equity was an inclusion criterion of the present review, so the classification of the outcomes was made in accordance with the other categories recommended by the Cochrane group of EPOC [15]. In Fig. 3, it can be seen that the most frequent group of outcomes was the reported outcome in (clinical) patients, followed by the social outcomes, and utilization, coverage and access outcomes.

## Discussion

### Main findings

The present review identified a large amount of existing literature on strategies or interventions that facilitate the reduction of health inequalities, which allowed a robust body of information to be put together that could help in health decision-making. Within the results, the wide variety of health conditions and intervened sectors with the aim of reducing health inequalities was highlighted, which reinforces the fact that the problem of inequalities occurs throughout the health system, and implies that work in reducing inequalities is intersectoral and multidisciplinary [68]. Most of the studies included focused on the general population, vulnerable populations, and minority populations. The subjects of general health and healthy lifestyles were the most addressed. According to the classification of the type of intervention, the domain covered most was the delivery arrangements, followed by the domain of implementation strategies. The most frequent group of outcomes was the reported outcome in (clinical) patients, followed by social outcomes.

The classification of strategies that facilitate the reduction of health inequalities identified in the present study were consistent with the three approaches described by Díez E and Peirò R [69], regarding strategies aimed at reducing health inequalities. The authors describe as a first approach those strategies aimed at the area of policies with economic changes, such as the increase in tobacco taxes, education policies aimed at vulnerable populations, economic aid, and community care in geographically remote areas, among others. In their second approach they group strategies for reforms in work and housing. Finally, in their third approach they include those strategies that promote an equal distribution of risk factors using the universalization of care and access systems through telemedicine, cultural approaches and intersectoral actions to improve lifestyles, educate the community and reduce risky behaviors [69].

### Findings by types of intervention

In addition, the included studies were oriented to a greater degree to strategies in the domain of delivery arrangements, where the strengthening of the coordination of care and management of care processes was displayed, which could be demonstrated by current efforts to transform the health system based on equity. Moreover, the need to strengthen health systems to achieve adequate access and universal health coverage is emphasized, as well as the guarantee of a comprehensive and high-quality care process to improve the states of health and well-being of the population [70].

In relation to the findings, care pathway-oriented strategies were the most common within this domain, highlighting the existence of interventions aimed at self-management in health, with positive results in particular topics such as weight loss or promotion of lifestyle changes [26–29]. In this sense, there is a significant interest in the strategies that seek to change the role in health care, which allow the inclusion of other participants to be involved in the health system and granting them an important role in maintaining it, such as self-care by patients and caregivers, the integration of entities and managers at the community level, or the creation and participation of medical-legal groups [71].

The second most frequent domain was that of implementation strategies, especially those directed at specific types of practice, conditions or environments, which highlighted the need to recognize the contextual conditions that influence the adequate maintenance of health, in particular living and working conditions as social determinants of health [52, 61]. These strategies aimed at improving these conditions have positive results, reaffirming the need to understand the individual as a subject within a social context that affects the quality of their well-being. These results are similar to those described in the review of systematic reviews of strategies to implement evidence-based interventions in low-income countries of Pantoja T *et. al.* 2017, where most of the available evidence focused on strategies aimed at health workers and health care recipients [11].

In addition, although there is a large variation in the types of strategies implemented, it is important to highlight the presence of interventions that recognize the intrinsic characteristics of the disadvantaged population. The implementation of these types of strategies encourages the acquisition of healthy styles and habits, which removes the focus of health from the disease and places it on the promotion and maintenance of health [12]. Additionally, some studies agree with the results on the integration of strategies based on the use of technologies and guided education, which showed positive results, particularly in clinical decision-making, consolidation and strengthening of health systems [70, 72].

EPOC taxonomy permits a structure for the classification of different interventions or strategies that help reduce health inequalities. This taxonomy proposed by the Cochrane Organization is aligned with the objectives of various groups aimed at reducing health inequality, such as “*Cochrane Child Health Field, Cochrane Public Health Review Group, Cochrane Developmental, Psychosocial and Learning Problems Group*”, which analyze the interventions in order to improve professional practices and the offer of health services, through continuing education of professionals and facilities in insurance for patients [73]. However, despite the clarity of the EPOC taxonomy for the inclusion of the various interventions, a problem that limits the use of this tool was identified, and is related to the high variability in the information reported in the different studies, the level of detail used to describe the strategies, the variability in the language used by the authors who report the results [71] and in general the quality of the studies included in the systematic reviews analyzed. Therefore, a need has arisen for a process of awareness regarding the reporting of results and necessary details in intervention studies, which allow for sufficient understanding at the methodological level and therefore the reproducibility of the results.

Finally, it is recommended that academia, policymakers, and practitioners develop and implement health action programs focused on equity to reduce health inequalities through strategies or interventions focused on the care pathway, intersectoral and multidisciplinary that including all sectors of the health system.

## Conclusion

The main strategies that facilitate the reduction of health inequalities focus on general health issues and the impact on healthy lifestyles, allowing us to observe that the objective is not only focused on the disease but also on the care pathway, that varies by illnesses, disease patterns, locality and multiple factors involved. In addition, these strategies must be intersectoral and multidisciplinary in nature, including all sectors of the health system. It is essential to continue generating interventions focused on strengthening health systems to achieve adequate universal health coverage, with a comprehensive and high-quality care process that leads to the reduction of health inequalities.

## Supplementary information

**Additional file 1.** Evidence search report in electronic databases.

**Additional file 2.** List of studies excluded and reasons for their exclusion.

**Additional file 3.** Characteristics of studies included in the evidence synthesis.

## Data Availability

Not Applicable.

## Abbreviations

LRs: Literature reviews
EPOC: Effective Practice and Organization of Care
CDSR: Cochrane Database of Systematic Reviews
VHL: Virtual Health Library

## References

[1] Arcaya MC, Arcaya AL, Subramanian SV (2015). Inequalities in health: definitions, concepts, and theories. Glob Health Action.

[2] Whitehead M. The concepts and principles of equity and health. Int J Heal Serv [Internet]. 1992;22:429–45. Available from: http://journals.sagepub.com/doi/10.2190/986L-LHQ6-2VTE-YRRN. 10.2190/986L-LHQ6-2VTE-YRRN 1644507

[3] Reducir las desigualdades entre países y dentro de ellos - Desarrollo Sostenible. Available from: https://www.un.org/sustainabledevelopment/es/inequality/. Cited 2019 Oct 14.

[4] Lele S, Jayaraman T (2011). Equity in the context of sustainable development.

[5] Gwatkin DR (2017). Trends in health inequalities in developing countries. Lancet Glob Health.

[6] Braveman P (2006). Health disparities and health equity: concepts and measurement. Annu Rev Public Health.

[7] Tugwell P, O’Connor A, Andersson N, Mhatre S, Kristjansson E, Jacobsen MJ (2006). Reduction of inequalities in health: assessing evidence-based tools. Int J Equity Health.

[8] Cecchi C (2008). La place de l’information dans la décision en santé publique. Sante Publique (Paris).

[9] Barsanti S, Salmi L-R, Bourgueil Y, Daponte A, Pinzal E, Ménival S (2017). Strategies and governance to reduce health inequalities: evidences from a cross-European survey. Glob Health Res Policy.

[10] Friedman EA, Gostin LO, Kavanagh MM, Periago MR, Marmot M, Coates A (2019). Putting health equity at heart of universal coverage-the need for national programmes of action. BMJ.

[11] Pantoja T, Opiyo N, Ciapponi A, Dudley L, Gagnon M-P, Herrera CA, Pantoja T (2014). Implementation strategies for health systems in low-income countries: an overview of systematic reviews. Cochrane Database Syst Rev.

[12] Jongen CS, McCalman J, Bainbridge RG (2017). The implementation and evaluation of health promotion services and programs to improve cultural competency: a systematic scoping review. Front Public Health.

[13] Higgins JPT, Green S. Cochrane handbook for systematic reviews of interventions version 5.1.0 [updated March 2011]: Cochrane Collab; 2011. Available from: www.handbook.cochrane.org.

[14] Effective Practice and Organisation of Care (EPOC). EPOC taxonomy: Cochrane Eff. Pract. Organ. Care; 2015. Available from: https://epoc.cochrane.org/epoc-taxonomy. Cited 2019 Oct 10.

[15] Cochrane Effective Practice and Organisation of Care (EPOC) (2017). What outcomes should be reported in Cochrane Effective Practice and Organisation of Care (EPOC) reviews?. EPOC Resour Rev.

[16] Ciapponi A (2017). AMSTAR-2: herramienta de evaluación crítica de revisiones sistemáticas de estudios de intervenciones de salud. Evidencia.

[17] AMSTAR - Assessing the Methodological Quality of Systematic Reviews. Available from: https://amstar.ca/About_Amstar.php. Cited 2020 Aug 17.

[18] Durand MA, Carpenter L, Dolan H, Bravo P, Mann M, Bunn F (2014). Do interventions designed to support shared decision- making reduce health inequalities? A systematic review and meta-analysis. PLoS One.

[19] Pottie K, Medu O, Welch V, Dahal GP, Tyndall M, Rader T, et al. Effect of rapid HIV testing on HIV incidence and services in populations at high risk for HIV exposure: an equity-focused systematic review. BMJ Open [Internet]. 2014;4:e006859. Available from: https://bmjopen.bmj.com/lookup/doi/10.1136/bmjopen-2014-006859. 10.1136/bmjopen-2014-006859 PMC4267075 25510889

[20] Speyer R, Denman D, Wilkes-Gillan S, Chen Y, Bogaardt H, Kim J (2018). Effects of telehealth by allied health professionals and nurses in rural and remote areas: a systematic review and meta-analysis. J Rehabil Med.

[21] Antoniades J, Mazza D, Brijnath B. Efficacy of depression treatments for immigrant patients: results from a systematic review. BMC Psychiatry [Internet]. 2014;14:176. Available from: http://bmcpsychiatry.biomedcentral.com/articles/10.1186/1471-244X-14-176. 10.1186/1471-244X-14-176 PMC4084503 24930429

[22] Kristjansson E, Dk F, Liberato S, Jandu MB, Welch V, Batal M (2015). Food supplementation for improving the physical and psychosocial health of socio-economically disadvantaged children aged three months to five years (Review).

[23] Sharma BB, Jones L, Loxton DJ, Booth D, Smith R (2018). Systematic review of community participation interventions to improve maternal health outcomes in rural South Asia. BMC Pregnancy Childbirth.

[24] Mosdøl A, Lidal IB, Straumann GH, Vist GE, Mosdøl A (2015). Targeted mass media interventions promoting healthy behaviours to reduce risk of non-communicable diseases in adult, ethnic minorities. Cochrane Database Syst Rev.

[25] Cairns JM, Bambra C, Hillier-Brown FC, Moore HJ, Summerbell CD (2015). Weighing up the evidence: a systematic review of the effectiveness of workplace interventions to tackle socio-economic inequalities in obesity. J Public Health (United Kingdom).

[26] Escribà-Agüir V, Rodríguez-Gómez M, Ruiz-Pérez I (2016). Effectiveness of patient-targeted interventions to promote cancer screening among ethnic minorities: a systematic review. Cancer Epidemiol.

[27] Walton-Moss B, Samuel L, Nguyen TH, Commodore-Mensah Y, Hayat MJ, Szanton SL (2014). Community-based cardiovascular health interventions in vulnerable populations. J Cardiovasc Nurs.

[28] Njeru JW, Wieland ML, Kwete G, Tan EM, Breitkopf CR, Agunwamba AA (2018). Diabetes mellitus management among patients with limited English proficiency: a systematic review and meta-analysis. J Gen Intern Med.

[29] Melvin CL, Jefferson MS, Rice LJ, Nemeth LS, Wessell AM, Nietert PJ (2017). A systematic review of lifestyle counseling for diverse patients in primary care. Prev Med (Baltim).

[30] Butel J, Braun KL (2019). The role of collective efficacy in reducing health disparities: a systematic review. Fam Community Health.

[31] Nathan AG, Marshall IM, Cooper JM, Huang ES (2016). Use of decision aids with minority patients: a systematic review. J Gen Intern Med.

[32] Martinez O, Boles J, Muñoz-Laboy M, Levine EC, Ayamele C, Eisenberg R (2017). Bridging health disparity gaps through the use of medical legal partnerships in patient care: a systematic review. J Law Med Ethics.

[33] Harris J, Springett J, Croot L, Booth A, Campbell F, Thompson J (2015). Can community-based peer support promote health literacy and reduce inequalities? A realist review. Public Health Res.

[34] Banna J, Bersamin A (2018). Community involvement in design, implementation and evaluation of nutrition interventions to reduce chronic diseases in indigenous populations in the U.S.: a systematic review. Int J Equity Health.

[35] Coughlin SS, Smith SA (2017). Community-based participatory research to promote healthy diet and nutrition and prevent and control obesity among African-Americans: a literature review. J Racial Ethn Health Disparities.

[36] Oliver-Williams C, Brown E, Devereux S, Fairhead C, Holeman I (2017). Using mobile phones to improve vaccination uptake in 21 low- and middle-income countries: systematic review. JMIR Mhealth Uhealth.

[37] Pratt CA, Loria CM, Arteaga SS, Nicastro HL, Lopez-Class M, de Jesus JM (2017). A systematic review of obesity disparities research. Am J Prev Med.

[38] Vallury KD, Jones M, Oosterbroek C (2015). Computerized cognitive behavior therapy for anxiety and depression in rural areas: a systematic review. J Med Internet Res.

[39] Batsis JA, Dimilia PR, Seo LM, Fortuna KL, Kennedy MA, Blunt HB (2019). Effectiveness of ambulatory telemedicine care in older adults: a systematic review.

[40] Hu D, Juarez DT, Yeboah M, Castillo TP (2014). Interventions to increase medication adherence in African-American and Latino populations: a literature review. Hawaii J Med Public Health.

[41] Anderson-Lewis C, Darville G, Mercado RE, Howell S, Di Maggio S (2018). mHealth technology use and implications in historically underserved and minority populations in the United States: systematic literature review. JMIR Mhealth Uhealth.

[42] Parker S, Prince A, Thomas L, Song H, Milosevic D, Harris MF (2018). Electronic, mobile and telehealth tools for vulnerable patients with chronic disease: a systematic review and realist synthesis. BMJ Open.

[43] Hamilton K, Tolfree R, Mytton J (2018). A systematic review of active case-finding strategies for tuberculosis in homeless populations. Int J Tuberc Lung Dis.

[44] Lee-Tauler SY, Eun J, Corbett D, Collins PY (2018). A systematic review of interventions to improve initiation of mental health care among racial-ethnic minority groups. Psychiatr Serv.

[45] Byrne A, Hodge A, Jimenez-Soto E, Morgan A (2014). What works? Strategies to increase reproductive, maternal and child health in difficult to access mountainous locations: a systematic literature review. PLoS One.

[46] Robinson JL, Narasimhan M, Amin A, Morse S, Beres LK, Yeh PT (2017). Interventions to address unequal gender and power relations and improve self-efficacy and empowerment for sexual and reproductive health decision-making for women living with HIV: a systematic review. PLoS One.

[47] Luque JS, Logan A, Soulen G, Armeson KE, Garrett DM, Davila CB (2019). Systematic review of mammography screening educational interventions for Hispanic women in the United States. J Cancer Educ.

[48] Rees I, Jones D, Chen H, Macleod U (2018). Interventions to improve the uptake of cervical cancer screening among lower socioeconomic groups: a systematic review. Prev Med (Baltim).

[49] Clifford A, Mccalman J, Bainbridge R, Tsey K (2015). Interventions to improve cultural competency in health care for indigenous peoples of Australia, New Zealand, Canada and the USA: a systematic review. Int J Qual Health Care.

[50] Barley EA, Borschmann RD, Walters P, Tylee A. Interventions to encourage uptake of cancer screening for people with severe mental illness. Cochrane Database Syst Rev. 2016;7 Available from: http://doi.wiley.com/10.1002/14651858.CD009641.pub3. 10.1002/14651858.CD009641.pub2 23857563

[51] Man LC, DiCarlo M, Lambert E, Sifri R, Romney M, Fleisher L (2018). A learning community approach to identifying interventions in health systems to reduce colorectal cancer screening disparities. Prev Med Rep.

[52] Rodrigues AL, Ball J, Ski C, Stewart S, Carrington MJ (2016). A systematic review and meta-analysis of primary prevention programmes to improve cardio-metabolic risk in non-urban communities. Prev Med (Baltim).

[53] Starbird LE, DiMaina C, Sun CA, Han HR (2019). A systematic review of interventions to minimize transportation barriers among people with chronic diseases. J Community Health.

[54] Blanchard AK, Prost A, Houweling TAJ (2019). Effects of community health worker interventions on socioeconomic inequities in maternal and newborn health in low-income and middle- income countries: a mixed-methods systematic review.

[55] Kim K, Choi JS, Choi E, Nieman CL, Joo JH, Lin FR (2016). Effects of community-based health worker interventions to improve chronic disease management and care among vulnerable populations: a systematic review. Am J Public Health.

[56] Jia L, Yuan B, Huang F, Lu Y, Garner P, Meng Q (1994). Experiencia, intersubjetividad y existencia. Hacia una teoría-práctica de la Etnografía. Run Arch para las Ciencias del Hombre.

[57] Tovar A, Renzaho AMN, Guerrero AD, Mena N, Ayala GX (2014). A systematic review of obesity prevention intervention studies among immigrant populations in the US. Curr Obes Rep.

[58] Gardner F, Leijten P, Mann J, Landau S, Harris V, Beecham J (2017). Could scale-up of parenting programmes improve child disruptive behaviour and reduce social inequalities? Using individual participant data meta-analysis to establish for whom programmes are effective and cost-effective. Public Health Res.

[59] Goudet SM, Griffiths PL, Bogin BA, Madise NJ. Nutritional interventions for preventing stunting in children (0 to 5 years) living in urban slums in low and middle-income countries (LMIC). Cochrane Database Syst Rev. 2015; Available from: http://doi.wiley.com/10.1002/14651858.CD011695. 10.1002/14651858.CD011695.pub2 PMC6572871 31204795

[60] Skeie MS, Klock KS (2018). Dental caries prevention strategies among children and adolescents with immigrant-or low socioeconomic backgrounds-do they work? A systematic review. BMC Oral Health.

[61] Van Rijn RM, Carlier BE, Schuring M, Burdorf A (2016). Work as treatment? The effectiveness of re-employment programmes for unemployed persons with severe mental health problems on health and quality of life: a systematic review and meta-analysis. Occup Environ Med.

[62] Ashman AM, Brown LJ, Collins CE, Rollo ME, Rae KM (2017). Factors associated with effective nutrition interventions for pregnant indigenous women: a systematic review. J Acad Nutr Diet.

[63] Pinzón Flórez CE, Díaz-Quijano DM, Yáñez Álvarez I, Mesa DC. Effectiveness of community workers on preventive measures to maternal and child health in low and middle income countries: systematic review of the literature. Rev Salud Uninorte [Internet]. Scieloco. 2015;31:309–28. Available from: http://rcientificas.uninorte.edu.co/index.php/salud/article/view/7621/7576.

[64] Verhagen I, Steunenberg B, De Wit NJ, Ros WJG (2014). Community health worker interventions to improve access to health care services for older adults from ethnic minorities: a systematic review. BMC Health Serv Res.

[65] Brown T, Platt S, Amos A (2013). Equity impact of European individual-level smoking cessation interventions to reduce smoking in adults: a systematic review.

[66] Brown T, Platt S, Amos A (2014). Equity impact of interventions and policies to reduce smoking in youth: systematic review.

[67] Brown T, Platt S, Amos A (2014). Equity impact of population-level interventions and policies to reduce smoking in adults: a systematic review. Drug Alcohol Depend.

[68] Plamondon KM, Caxaj CS, Graham ID, Bottorff JL (2019). Connecting knowledge with action for health equity: a critical interpretive synthesis of promising practices. Int J Equity Health.

[69] Díez E, Peirò R (2004). Intervenciones para disminuir las desigualdades en salud. Gac Sanit.

[70] Inequities and barriers in health systems. Pan Am. Heal. Organ. Available from: https://www.paho.org/salud-en-las-americas-2017/?p=59. Cited 2019 Nov 14.

[71] Mazza D, Bairstow P, Buchan H, Chakraborty SP, Van Hecke O, Grech C (2013). Refining a taxonomy for guideline implementation: results of an exercise in abstract classification. Implement Sci.

[72] Watkins K, Wood H, Schneider CR, Clifford R (2015). Effectiveness of implementation strategies for clinical guidelines to community pharmacy: a systematic review. Implement Sci.

[73] Roberts H. What works in reducing inequalities in child health? Policy Press; 2012. Available from: https://books.google.co.uk/books?id=QMkjjvJok5gC.

