# Supplementary material for: An overview of reviews on strategies to reduce health inequalities
Source: Int J Equity Health. 2020 Oct 28;19:192. doi: 10.1186/s12939-020-01299-w (PMC7594271; doi:10.1186/s12939-020-01299-w)
Supplement: Supplementary file 1 — Additional file 1. Evidence search report in electronic databases. [file 12939_2020_1299_MOESM1_ESM.docx]

Additional file 1. Evidence search report in electronic databases

| **Electronic search report No. 1** | |
| --- | --- |
| **Electronic database** | Ovid MEDLINE(R) 1946 to July Week 4 2019 |
| **Platform** | Ovid |
| **Date of search** | 04-08-2019 |
| **Range of date** | Last 5 years |
| **Restriction of language** | None |
| **Other limits** | Reviews (maximizes specificity) |
| **Search strategy (results)** | 1. Training.ab,ti. (311931) 2. exp Patient Education Handout/ or exp Education/ (779135) 3. exp Learning/ or exp Social Learning/ (367771) 4. exp Expert Testimony/ (13021) 5. exp Health Education/ (234313) 6. opinion leaders.ab,ti. (1021) 7. exp Health Planning/ (334194) 8. health care planning. ab,ti. (724) 9. health care facility. ab,ti. (1439) 10. exp Leadership/ (39167) 11. leaders opinion.ab,ti. (4) 12. exp Medical Audit/ or exp Clinical Audit/ (21731) 13. audit.ab,ti. (27708) 14. Reminder*.ab,ti. (9505) 15. exp Mass Media/ (44589) 16. mass medium.ab,ti (23) 17. endorsement.ab,ti. (4209) 18. strateg*.ab,ti. (812897) 19. Multifaceted.ab,ti. (14800) 20. multi-faceted.ab,ti. (2339) 21. user-friendly.ab,ti. (9298) 22. exp Decision Support Systems, Clinical/ or exp Decision Making, Computer-Assisted/ (142699) 23. Decision support systems.ab,ti. (2072) 24. (Checklist or tool).ab,ti. (389401) 25. Tailored.ab,ti. (40351) 26. exp Motivation/ (162303) 27. incentive.ab,ti. (9930) 28. exp Physician Incentive Plans/ (2221) 29. personnel management ab,ti. (381) 30. Compensation.ab,ti. (36440) 31. Intervention*.ab,ti. (761098) 32. Health Program.ab,ti. (4652) 33. Community-based intervention.ab,ti. (907) 34. 1 or 2 or 3 or 4 or 5 or 6 or 7 or 8 or 9 or 10 or 11 or 12 or 13 or 14 or 15 or 16 or 17 or 18 or 19 or 20 or 21 or 22 or 23 or 24 or 25 or 26 or 27 or 28 or 29 (3473902) 35. exp Healthcare Disparities/ or exp Health Status Disparities/ (26591) 36. Health* disparit*.ab,ti. (7414) 37. Disparit*.ab,ti. (47780) 38. health-care disparit*.ab,ti. (679) 39. health*care disparit*.ab,ti. (336) 40. (health* adj3 disparit*).ab,ti. (10564) 41. health status disparit*.ab,ti. (20) 42. disparities in health.ab,ti. (1493) 43. health inequ*.ab,ti. (4997) 44. inequ*.ab,ti. (27808) 45. ((economic level or social class or social determinants or social status or social position or social background or social circumstance* or socio-economic or socioeconomic or sociodemographic or socio-demographic) and (health or Health* disparit* or health inequ*)). ab,ti. (73276) 46. (determinant* of health and (disparit* or inequ*)).tw. (1524) 47. ((social disparit* or social inequ*) and health).tw. (2353) 48. (socioeconomically disadvantaged and (health or Health* disparit* or health inequ*)).tw. (527) 49. ((vulnerable or disadvantages) and (population* or group*) and (health or Health* disparit* or health inequ*)).tw. (12434) 50. ((disabled person* or emigrant* or immigrant* or jail population* or minority group* or minority population* or migrant worker* or poverty or prisoner* or race or racial or ethnic* or refugees or rural or urban or sexism or slum* or social discrimination or social marginalization or social segregation or (transients and migrants) or undocumented immigrant* or working poor or bisexual* or bigender female homosexuality or gay* or gender change or gender confirmation or gender disorder* or gender dysphoria or gender diverse or gender-diverse or gender diversity or gender identity or gender minorities or homosexual* or Cultural Deprivation or indigenous) and (health or Health* disparit* or health inequ*)).tw. (146502) 51. 31 or 32 or 33 or 34 or 35 or 36 or 37 or 38 or 39 or 40 or 41 or 42 or 43 or 44 or 45 or 46 (264154) 52. 30 and 47 (113022) 53. limit 48 to ("reviews (maximizes specificity)" and last 5 years) (1579) |
| **Number of references found** | 1579 |
| **Electronic search report No. 2** | |
| **Electronic database** | Embase |
| **Platform** | Elsevier |
| **Date of search** | 04/08/2019 |
| **Range of date** | Last 5 years |
| **Restriction of language** | None |
| **Other limits** | Reviews (maximizes specificity) |
| **Search strategy (results)** | 1. 'training'/exp OR training (676308) 2. 'patient education handout':ab,ti (36) 3. 'education'/exp (1413254) 4. learning:ab,ti (346854) 5. 'social learning'/exp (2693) 6. 'expert testimony':ab,ti (1632) 7. 'health education'/exp (313133) 8. 'opinion leaders':ab,ti (1614) 9. 'health care planning'/exp (97392) 10. 'health planning':ab,ti (4073) 11. 'health care facility'/exp (1541421) 12. 'leadership'/exp (62033) 13. 'leaders opinion':ab,ti (5) 14. 'medical audit':ab,ti (962) 15. 'clinical audit'/exp (49483) 16. audit:ab,ti (68010) 17. reminder*:ab,ti (17596) 18. 'mass medium'/exp (17747) 19. endorsement:ab,ti (6483) 20. strateg*:ab,ti (1267230) 21. multifaceted:ab,ti (22120) 22. 'multi-faceted':ab,ti (4209) 23. 'user-friendly':ab,ti (14672) 24. 'decision support system'/exp (22886) 25. 'clinical decision support system'/exp (2353) 26. 'decision making, computer-assisted':ab,ti (2) 27. checklist:ab,ti OR tool:ab,ti (646823) 28. tailored:ab,ti (69390) 29. 'motivation'/exp (100663) 30. 'incentive'/exp (33) 31. 'personnel management'/exp (88386) 32. 'physician incentive plans':ab,ti (17) 33. 'compensation'/exp (12959) 34. intervention*:ab,ti (1255719) 35. 'health program'/exp (125083) 36. 'community-based intervention':ab,ti (1263) 37. #1 OR #2 OR #3 OR #4 OR #5 OR #6 OR #7 OR #8 OR #9 OR #10 OR #11 OR #12 OR #13 OR #14 OR #15 OR #16 OR #17 OR #18 OR #19 OR #20 OR #21 OR #22 OR #23 OR #24 OR #25 OR #26 OR #27 OR #28 OR #29 OR #30 OR #31 OR #32 OR #33 OR #34 OR #35 OR #36 (6004497) 38. 'health care disparity'/exp (13761) 39. 'health disparity'/exp (17478) 40. 'health status disparit*':ab,ti (26) 41. 'health* disparit*':ab,ti (11174) 42. 'disparit*':ab,ti (74893) 43. 'health*care disparit*':ab,ti (627) 44. 'health status disparit*.':ab,ti (26) 45. 'disparities in health':ab,ti (2027) 46. 'health inequ*':ab,ti (6639) 47. 'inequ*.':ab,ti (38191) 48. ('economic level':ab,ti OR 'social class':ab,ti OR 'social determinants':ab,ti OR 'social status':ab,ti OR 'social position':ab,ti OR 'social background':ab,ti OR 'social circumstance*':ab,ti OR 'socio-economic':ab,ti OR 'socioeconomic':ab,ti OR 'sociodemographic':ab,ti OR 'socio-demographic':ab,ti) AND (health:ab,ti OR 'health* disparit*':ab,ti OR 'health inequ*':ab,ti) (108954) 49. 'determinant* of health':ab,ti AND (disparit*:ab,ti OR inequ*:ab,ti) (2063) 50. ('social disparit*':ab,ti OR 'social inequ*':ab,ti) AND health:ab,ti (2980) 51. 'socioeconomically disadvantaged':ab,ti AND (health:ab,ti OR 'health* disparit*':ab,ti OR 'health inequ*':ab,ti) (754) 52. (vulnerable:ab,ti OR disadvantages:ab,ti) AND (population*:ab,ti OR group*:ab,ti) AND (health:ab,ti OR 'health* disparit*':ab,ti OR 'health inequ*':ab,ti) (20213) 53. ('disabled person*' OR emigrant* OR immigrant* OR 'jail population*' OR 'minority group*' OR 'minority population*' OR 'migrant worker*' OR poverty OR prisoner* OR race OR racial OR ethnic* OR refugees OR rural OR urban OR sexism OR slum* OR 'social discrimination' OR 'social marginalization' OR 'social segregation' OR (transients AND migrants) OR 'undocumented immigrant*' OR 'working poor' OR bisexual* OR 'bigender female homosexuality' OR gay* OR 'gender change' OR 'gender confirmation' OR 'gender disorder*' OR 'gender dysphoria' OR 'gender diverse' OR 'gender-diverse' OR 'gender diversity' OR 'gender identity' OR 'gender minorities' OR homosexual* OR 'cultural deprivation' OR indigenous:ab,ti) AND (health:ab,ti OR 'health* disparit*':ab,ti OR 'health inequ*':ab,ti) (285214) 54. #38 OR #39 OR #40 OR #41 OR #42 OR #43 OR #44 OR #45 OR #46 OR #47 OR #48 OR #49 OR #50 OR #51 OR #52 OR #53 (449649) 55. #37 AND #54 (225588) 56. #55 AND (2014:py OR 2015:py OR 2016:py OR 2017:py OR 2018:py OR 2019:py OR 2020:py) AND [embase]/lim NOT ([embase]/lim AND [medline]/lim) AND 'Review'/it (1133) |
| **Number of references found** | 1133 |
| **Electronic search report No. 3** | |
| **Electronic database** | Cochrane Database of Systematic Reviews 2005 – to July 31, 2019 |
| **Platform** | Ovid |
| **Date of search** | 04/08/2019 |
| **Range of date** | Last 5 years |
| **Restriction of language** | None |
| **Other limits** | None |
| **Search strategy (results)** | 1. Training.ab,ti. (349) 2. (Patient Education Handout or Education).ab,ti. (278) 3. (Learning or Social Learning).ab,ti. (42) 4. Expert Testimony.ab,ti. (0) 5. Health Education.ab,ti. (24) 6. opinion leaders.ab,ti. (3) 7. Health Planning.ab,ti. (0) 8. health care planning.ab,ti. (0) 9. health care facility.ab,ti. (1) 10. leadership.ab,ti. (2) 11. leaders opinion.ab,ti. (0) 12. (Medical Audit or Clinical Audit).ab,ti. (0) 13. audit.ab,ti. (15) 14. Reminder*.ab,ti. (37) 15. Mass Media.ab,ti. (13) 16. mass medium.ab,ti. (0) 17. endorsement.ab,ti. (2) 18. strateg*.ab,ti. (2463) 19. Multifaceted.ab,ti. (23) 20. multi-faceted.ab,ti. (11) 21. user-friendly.ab,ti. (0) 22. (Decision Support Systems, Clinical or Decision Making, Computer-Assisted).ab,ti. (0) 23. Decision support systems.ab,ti. (6) 24. (Checklist or tool).ab,ti. (455) 25. Tailored.ab,ti. (38) 26. Motivation.ab,ti. (24) 27. incentive.ab,ti. (15) 28. Physician Incentive Plans.ab,ti. (0) 29. personnel management.ab,ti. (0) 30. Compensation.ab,ti. (1) 31. Intervention*.ab,ti. (4858) 32. Health Program.ab,ti. (1) 33. Community-based intervention.ab,ti. (3) 34. 1 or 2 or 3 or 4 or 5 or 6 or 7 or 10 or 11 or 12 or 13 or 14 or 15 or 17 or 18 or 19 or 20 or 21 or 22 or 23 or 24 or 25 or 26 or 27 or 28 or 30 or 31 or 32 or 33 (6451) 35. (Healthcare Disparities or Health Status Disparities).ab,ti. (0) 36. Health* disparit*.ab,ti. (4) 37. Disparit*.ab,ti. (12) 38. health-care disparit*.ab,ti. (0) 39. health*care disparit*.ab,ti. (0) 40. (health* adj3 disparit*).ab,ti. (4) 41. health status disparit*.ab,ti. (0) 42. disparities in health.ab,ti. (1) 43. health inequ*.ab,ti. (5) 44. inequ*.ab,ti. (12) 45. ((economic level or social class or social determinants or social status or social position or social background or social circumstance* or socio-economic or socioeconomic or sociodemographic or socio-demographic) and (health or Health* disparit* or health inequ*)).ab,ti. (43) 46. (determinant* of health and (disparit* or inequ*)).ab,ti. (0) 47. ((social disparit* or social inequ*) and health).ab,ti. (0) 48. (socioeconomically disadvantaged and (health or Health* disparit* or health inequ*)).ab,ti. (1) 49. ((vulnerable or disadvantages) and (population* or group*) and (health or Health* disparit* or health inequ*)).ab,ti. (23) 50. ((disabled person* or emigrant* or immigrant* or jail population* or minority group* or minority population* or migrant worker* or poverty or prisoner* or race or racial or ethnic* or refugees or rural or urban or sexism or slum* or social discrimination or social marginalization or social segregation or (transients and migrants) or undocumented immigrant* or working poor or bisexual* or bigender female homosexuality or gay* or gender change or gender confirmation or gender disorder* or gender dysphoria or gender diverse or gender-diverse or gender diversity or gender identity or gender minorities or homosexual* or Cultural Deprivation or indigenous) and (health or Health* disparit* or health inequ*)).ab,ti. (80) 51. 35 or 36 or 37 or 38 or 39 or 40 or 41 or 42 or 43 or 44 or 45 or 46 or 47 or 48 or 49 or 50 (145) 52. 34 and 51 (112) 53. limit 52 to last 5 years (60) |
| **Number of references found** | 60 |
| **Electronic search report No. 4** | |
| **Electronic database** | LILACS |
| **Platform** | VHL Regional Portal - Information and Knowledge for Health  <http://search.bvsalud.org/portal> |
| **Date of search** | 04/08/2019 |
| **Range of date** | Last 5 years |
| **Restriction of language** | None |
| **Other limits** | Systematic review |
| **Search strategy (results)** | 1. training OR (patient education handout) OR education OR learning OR (social learning) OR (expert testimony) OR (health education) OR (opinion leaders) OR (health planning) OR (health care planning) OR (health care facility) OR leadership OR (leaders opinion) OR (medical audit) OR (clinical audit) OR audit OR reminder* OR (mass media) OR (mass medium) OR endorsement OR strateg* OR multifaceted OR multi-faceted OR user-friendly OR (decision support systems, clinical) OR (decision making, computer-assisted) OR (decision support systems) OR checklist OR tool OR tailored OR motivation OR incentive OR (physician incentive plans) OR (personnel management) OR compensation OR intervention* OR (health program) OR (community-based intervention) *(Title, abstract, subject)* (5830366) 2. (healthcare disparities) OR (health status disparities) OR (health* disparit*) OR disparit* OR (health-care disparit*) OR (health*care disparit*) OR (disparities in health) OR (health inequ*) OR (inequ*) OR ((economic level OR social class OR social determinants OR social status OR social position OR social background OR social circumstance* OR socio-economic OR socioeconomic OR sociodemographic OR socio-demographic) AND (health OR health* disparit* OR health inequ*)) OR ((determinant* of health) AND (disparit* OR inequ*)) OR ((social disparit* OR social inequ*) AND health) OR ((socioeconomically disadvantaged) AND (health OR health* disparit* OR health inequ*)) OR ((vulnerable OR disadvantages) AND (population* OR group*) AND (health OR health* disparit* OR health inequ*)) OR ((disabled person* OR emigrant* OR immigrant* OR jail population* OR minority group* OR minority population* OR migrant worker* OR poverty OR prisoner* OR race OR racial OR ethnic* OR refugees OR rural OR urban OR sexism OR slum* OR social discrimination OR social marginalization OR social segregation OR (transients AND migrants) OR undocumented immigrant* OR working poor OR bisexual* OR bigender female homosexuality OR gay* OR gender change OR gender confirmation OR gender disorder* OR gender dysphoria OR gender diverse OR gender-diverse OR gender diversity OR gender identity OR gender minorities OR homosexual* OR cultural deprivation OR indigenous) AND (health OR health* disparit* OR health inequ*)) *(Title, abstract, subject)* (262418) 3. 1 AND 2 (134869)   Filtros: "LILACS" AND type_of_study:("systematic_reviews") AND year_cluster:("2017" OR "2018" OR "2016" OR "2014" OR "2015") (56) |
| **Number of references found** | 56 |
| **Electronic search report No. 5** | |
| **Electronic database** | Scopus |
| **Platform** | Elsevier |
| **Date of search** | 04/08/2019 |
| **Range of date** | Last 5 years |
| **Restriction of language** | None |
| **Other limits** | Review |
| **Search strategy (results)** | (TITLE-ABS-KEY((Training) OR (Patient Education Handout) OR (Education) OR (Learning) OR (Social Learning) OR (Expert Testimony) OR (Health Education) OR (opinion leaders) OR (Health Planning) OR (health care planning) OR (health care facility) OR (leadership) OR (leaders opinion) OR (Medical Audit) OR (Clinical Audit) OR audit OR Reminder* OR (Mass Media) OR (mass medium) OR (endorsement) OR (strateg*) OR (Multifaceted) OR (multi-faceted) OR (user-friendly) OR (Decision Support Systems, Clinical) OR (Decision Making, Computer-Assisted) OR (Decision support systems) OR (Checklist) OR (tool) OR (Tailored) OR (Motivation) OR (incentive) OR (Physician Incentive Plans) OR (personnel management) OR (Compensation) OR (Intervention*) OR (Health Program) OR (Community-based intervention))) AND (TITLE-ABS-KEY((Healthcare Disparities) OR (Health Status Disparities) OR (Health* disparit*) OR (disparit*) OR (health-care disparit*) OR (health*care disparit*) OR (disparities in health) OR (health inequ*) OR (inequ*) OR (((vulnerable) OR (disadvantages)) AND ((population*) OR (group*)) AND ((health) OR (Health* disparit*) OR (health inequ*))))) AND (LIMIT-TO ( SRCTYPE,"j")) AND (LIMIT-TO (DOCTYPE,"re")) AND (LIMIT-TO (PUBYEAR,2020) OR LIMIT-TO (PUBYEAR,2019) OR LIMIT-TO (PUBYEAR,2018) OR LIMIT-TO (PUBYEAR,2017) OR LIMIT-TO (PUBYEAR,2016) OR LIMIT-TO (PUBYEAR,2015) ) AND (LIMIT-TO (EXACTKEYWORD,"Review")) AND (LIMIT-TO (openaccess,1)) |
| **Number of references found** | 885 |
| **Electronic search report No. 6** | |
| **Electronic database** | Scielo |
| **Platform** | <https://search.scielo.org/> |
| **Date of search** | 04/08/2019 |
| **Range of date** | Last 5 years |
| **Restriction of language** | None |
| **Other limits** | Review |
| **Search strategy (results)** | (training OR (patient education handout) OR education OR learning OR (social learning) OR (expert testimony) OR (health education) OR (opinion leaders) OR (health planning) OR (health care planning) OR (health care facility) OR leadership OR (leaders opinion) OR (medical audit) OR (clinical audit) OR audit OR reminder* OR (mass media) OR (mass medium) OR endorsement OR strateg* OR multifaceted OR multi-faceted OR user-friendly OR (decision support systems, clinical) OR (decision making, computer-assisted) OR (decision support systems) OR checklist OR tool OR tailored OR motivation OR incentive OR (physician incentive plans) OR (personnel management) OR compensation OR intervention* OR (health program) OR (community-based intervention) ) AND ((healthcare disparities) OR (health status disparities) OR (health* disparit*) OR disparit* OR (health-care disparit*) OR (health*care disparit*) OR (disparities in health) OR (health inequ*) OR (inequ*) OR ((economic level OR social class OR social determinants OR social status OR social position OR social background OR social circumstance* OR socio-economic OR socioeconomic OR sociodemographic OR socio-demographic) AND (health OR health* disparit* OR health inequ*)) OR ((determinant* of health) AND (disparit* OR inequ*)) OR ((social disparit* OR social inequ*) AND health) OR ((socioeconomically disadvantaged) AND (health OR health* disparit* OR health inequ*)) OR ((vulnerable OR disadvantages) AND (population* OR group*) AND (health OR health* disparit* OR health inequ*)) OR ((disabled person* OR emigrant* OR immigrant* OR jail population* OR minority group* OR minority population* OR migrant worker* OR poverty OR prisoner* OR race OR racial OR ethnic* OR refugees OR rural OR urban OR sexism OR slum* OR social discrimination OR social marginalization OR social segregation OR (transients AND migrants) OR undocumented immigrant* OR working poor OR bisexual* OR bigender female homosexuality OR gay* OR gender change OR gender confirmation OR gender disorder* OR gender dysphoria OR gender diverse OR gender-diverse OR gender diversity OR gender identity OR gender minorities OR homosexual* OR cultural deprivation OR indigenous) AND (health OR health* disparit* OR health inequ*)) ) AND year_cluster:("2018" OR "2015" OR "2017" OR "2016" OR "2014") AND type:("review-article") |
| **Number of references found** | 54 |
| **Electronic search report No. 7** | |
| **Electronic database** | Epistemonikos |
| **Platform** | <https://www.epistemonikos.org/> |
| **Date of search** | 04/08/2019 |
| **Range of date** | Last 5 years |
| **Restriction of language** | None |
| **Other limits** | Systematic review |
| **Search strategy (results)** | (title:((Training) OR (Patient Education Handout) OR (Education) OR (Learning) OR (Social Learning) OR (Expert Testimony) OR (Health Education) OR (opinion leaders) OR (Health Planning) OR (health care planning) OR (health care facility) OR (leadership) OR (leaders opinion) OR (Medical Audit) OR (Clinical Audit) OR audit OR Reminder* OR (Mass Media) OR (mass medium) OR (endorsement) OR (strateg*) OR (Multifaceted) OR (multi-faceted) OR (user-friendly) OR (Decision Support Systems, Clinical) OR (Decision Making, Computer-Assisted) OR (Decision support systems) OR (Checklist) OR (tool) OR (Tailored) OR (Motivation) OR (incentive) OR (Physician Incentive Plans) OR (personnel management) OR (Compensation) OR (Intervention*) OR (Health Program) OR (Community-based intervention)) OR abstract:((Training) OR (Patient Education Handout) OR (Education) OR (Learning) OR (Social Learning) OR (Expert Testimony) OR (Health Education) OR (opinion leaders) OR (Health Planning) OR (health care planning) OR (health care facility) OR (leadership) OR (leaders opinion) OR (Medical Audit) OR (Clinical Audit) OR audit OR Reminder* OR (Mass Media) OR (mass medium) OR (endorsement) OR (strateg*) OR (Multifaceted) OR (multi-faceted) OR (user-friendly) OR (Decision Support Systems, Clinical) OR (Decision Making, Computer-Assisted) OR (Decision support systems) OR (Checklist) OR (tool) OR (Tailored) OR (Motivation) OR (incentive) OR (Physician Incentive Plans) OR (personnel management) OR (Compensation) OR (Intervention*) OR (Health Program) OR (Community-based intervention))) AND (title:((Healthcare Disparities) OR (Health Status Disparities) OR (Health* disparit*) OR (disparit*) OR (health-care disparit*) OR (health*care disparit*) OR (disparities in health) OR (health inequ*) OR (inequ*) OR (((vulnerable) OR (disadvantages)) AND ((population*) OR (group*)) AND ((health) OR (Health* disparit*) OR (health inequ*)))) OR abstract:((Healthcare Disparities) OR (Health Status Disparities) OR (Health* disparit*) OR (disparit*) OR (health-care disparit*) OR (health*care disparit*) OR (disparities in health) OR (health inequ*) OR (inequ*) OR (((vulnerable) OR (disadvantages)) AND ((population*) OR (group*)) AND ((health) OR (Health* disparit*) OR (health inequ*))))) |
| **Number of references found** | 1375 |
