# Supplementary material for: An overview of reviews on strategies to reduce health inequalities
Source: Int J Equity Health. 2020 Oct 28;19:192. doi: 10.1186/s12939-020-01299-w (PMC7594271; doi:10.1186/s12939-020-01299-w)
Supplement: Supplementary file 2 — Additional file 2. List of studies excluded and reasons for their exclusion. [file 12939_2020_1299_MOESM2_ESM.docx]

Additional file 2. List of studies excluded and reasons for their exclusion

| **Nº** | **Author** | **Title** | **Reason for exclusion** |
| --- | --- | --- | --- |
| **1** | Duarte, Goncalo Silva (1) | Analysis of the Cochrane Review: Non-medical Prescribing versus Medical Prescribing for Acute and Chronic Disease Management in Primary and Secondary Care. Cochrane Database Syst Rev. 2016;11:CD011227. | Type of idiom: different from Spanish and English  Type of study: opinion article |
| **2** | Guignard, R (2) | Interventions for smoking cessation among low socioeconomic status smokers: a literature review. | Type of idiom: different from Spanish and English |
| **3** | Fekete, Christine (3) | Social inequalities in nutrition: Evidence, causes and interventions | Type of idiom: different from Spanish and English |
| **4** | Li, Wen-Wen (4) | The Use of Telemedicine Interventions to Improve Hypertension Management Among Racial Ethnic Minorities: A Systematic Review | Type of idiom: different from Spanish and English |
| **5** | CA, Rozendo (5) | A critical review of social and health inequalities in the nursing curriculum. | Type of included studies: qualitative studies |
| **6** | Ortiz, J R (6) | A global review of national influenza immunization policies: Analysis of the 2014 WHO/UNICEF Joint Reporting Form on immunization | Type of study: Narrative review |
| **7** | Gallagher, Ruth W (7) | A meta-analysis of educational interventions designed to enhance cultural competence in professional nurses and nursing students. | The strategies are not focused on reducing health inequities |
| **8** | Mann-Jackson, L (8) | A Qualitative Systematic Review of Cigarette Smoking Cessation Interventions for Persons Living with HIV. | The strategies are not focused on reducing health inequities |
| **9** | Robertson, Jan (9) | A Review of Trends in Indigenous Australian Tobacco Research (From 2004 to 2013), its Associated Outputs and Evidence of Research Translation. | Type of included studies and the strategies are not focused on reducing health inequities |
| **10** | Yehia, F (10) | A roadmap to parity in mental health financing: the case of Lebanon. | Type of included studies and the strategies are not focused on reducing health inequities |
| **11** | Farmer, J (11) | A scoping review of the association between rural medical education and rural practice location. | Type of included studies: qualitative studies |
| **12** | Lopez-Class, M (12) | A strategic framework for utilizing late-stage (t4) translation research to address health inequities | Type of included studies and the strategies are not focused on reducing health inequities |
| **13** | Driscoll, Susan D (13) | A Systematic Review and Quantitative Meta-Analysis of the Accuracy of Visual Inspection for Cervical Cancer Screening: Does Provider Type or Training Matter? | Type of included studies and the strategies are not focused on reducing health inequities |
| **14** | de Dassel, Jessica Langloh (14) | A systematic review of adherence in Indigenous Australians: an opportunity to improve chronic condition management. | Type of included studies |
| **15** | HR, Han (15) | A Systematic Review of Community Health Center Based Interventions for People with Diabetes. | The outcomes are not focused on reducing health inequities |
| **16** | Brooker, Katie (16) | A systematic review of interventions aiming to improve involvement in physical activity among adults with intellectual disability. | The outcomes are not focused on reducing health inequities |
| **17** | Conrad, E (17) | A systematic review of obesity interventions targeting anthropometric changes in youth with intellectual disabilities. | The outcomes are not focused on reducing health inequities |
| **18** | C.A., Pelletier (18) | A systematic review of physical activity interventions to improve physical fitness and health outcomes among Indigenous adults living in Canada | The outcomes are not focused on reducing health inequities |
| **19** | AL, Stangl (19) | A systematic review of selected human rights programs to improve HIV-related outcomes from 2003 to 2015: what do we know? | Type of included studies: observational studies |
| **20** | Newton, R L Jr (20) | A systematic review of weight loss, physical activity and dietary interventions involving African American men. | The outcomes are not focused on reducing health inequities |
| **21** | Pressick, Elizabeth L (21) | A systematic review on research into the effectiveness of group-based sport and exercise programs designed for Indigenous adults. | The outcomes are not focused on reducing health inequities |
| **22** | M., Johri (22) | Adding interventions to mass measles vaccinations in India | Type of included studies |
| **23** | Lee, J (23) | Addressing health equity through action on the social determinants of health: A global review of policy outcome evaluation methods | Type of included studies: observational studies |
| **24** | Farrer, Linden (24) | Advocacy for health equity: a synthesis review. | Type of study: Narrative review |
| **25** | Chopel, Alison Marie (25) | An ecological community-based participatory research study of late diagnosed HIV/AIDS in Oakland, California: Investigating influential factors in racial/ ethnic health inequities. | Type of included studies |
| **26** | JL, Bottorff (26) | An Updated Review of Interventions that Include Promotion of Physical Activity for Adult Men. | The outcomes are not focused on reducing health inequities. |
| **27** | FL, Brown (27) | Annual Research Review: Breaking cycles of violence - a systematic review and common practice elements analysis of psychosocial interventions for children and youth affected by armed conflict. | The outcomes are not focused on reducing health inequities |
| **28** | Hatzenbuehler, Mark L (28) | Anti-bullying Policies and Disparities in Bullying: A State-Level Analysis. | Type of included studies |
| **29** | Koller, T S (29) | Applying the Innov8 approach for reviewing national health programmes to leave no one behind: lessons learnt from Indonesia | Type of included studies |
| **30** | Mawn, Lauren (30) | Are we failing young people not in employment, education or training (NEETs)? A systematic review and meta-analysis of re-engagement interventions. | The outcomes are not focused on reducing health inequities. |
| **31** | Njoroge, Martin (31) | Assessing the feasibility of eHealth and mHealth: a systematic review and analysis of initiatives implemented in Kenya. | It talks about eHealth implementation and its impact on health. However, it does not speak of its impact or reduction of inequities in health |
| **32** | Farnbach, Sara (32) | Australian Aboriginal and Torres Strait Islander-focused primary healthcare social and emotional wellbeing research: a systematic review protocol. | Protocol |
| **33** | Rice, Kathleen (33) | Best Practices for the Prevention and Management of Diabetes and Obesity-Related Chronic Disease among Indigenous Peoples in Canada: A Review. | The outcomes are not focused on reducing health inequities |
| **34** | Rayner, G (34) | Can a pediatric department provide health care for vulnerable adolescents? | Type of study: Narrative review |
| **35** | T.W., Brown (35) | Centre-based day care for children younger than five years of age in low- and middle-income countries | The outcomes are not focused on reducing health inequities |
| **36** | Tsuji, H (36) | Challenges and solutions in immigrant occupational health in the United States: a literature review and comparative analysis. | Type of idiom: different from Spanish and English |
| **37** | Islam, Nadia S (37) | Characteristics of Asian American, Native Hawaiian, and Pacific Islander community health worker programs: a systematic review. | The outcomes are not focused on reducing health inequities |
| **38** | Cheng, T L (38) | Child health disparities: What can a clinician do? | Type of included studies |
| **39** | Camp, Nadine Lauer (39) | Childhood Healthy Behaviors Intervention in a Pediatric Primary Care Setting: Impact on Provider Practice. | Type of study: Narrative review |
| **40** | Killian-Farrell, Candace (40) | Childhood trauma and adolescent mental health: A transdisciplinary approach for social work research and practice. | The outcomes are not focused on reducing health inequities |
| **41** | AD, Racine (41) | Children's Health Insurance Program (CHIP): accomplishments, challenges, and policy recommendations. | Type of included studies |
| **42** | J.M., Carethers (42) | Clinical and genetic factors to inform reducing colorectal cancer disparitites in African Americans | Type of included studies |
| **43** | León Cabrera, Pablo (43) | Cobertura y acceso a los servicios de salud para el abordaje de la tuberculosis | Type of study: Narrative review |
| **44** | Cooper, L A (44) | Commentary: Training and mentoring the next generation of health equity researchers: Insights from the field | Type of study: Narrative review |
| **45** | Schiavo, Renata (45) | Communicating risk and promoting disease mitigation measures in epidemics and emerging disease settings. | The outcomes are not focused on reducing health inequities |
| **46** | McFadden, Alison (46) | Community engagement to enhance trust between Gypsy/Travellers, and maternity, early years' and child dental health services: protocol for a multi-method exploratory study. | The outcomes are not focused on reducing health inequities |
| **47** | Eslava-Schmalbach, J (47) | Conceptual framework of equity-focused implementation research for health programs (EquIR). | Type of study |
| **48** | Lyman, D Russell (48) | Consumer and family psychoeducation: assessing the evidence. | The outcomes are not focused on reducing health inequities |
| **49** | Orkin, A M (49) | Defining and measuring health equity in research on task shifting in high-income countries: A systematic review | The strategies are not focused on reducing health inequities |
| **50** | Mazarello Paes, V (50) | Determinants of sugar-sweetened beverage consumption in young children: a systematic review. | The outcomes are not focused on reducing health inequities |
| **51** | Abdul Manaf, Mohd Rizal (51) | Dialysis Provision and Implications of Health Economics on Peritoneal Dialysis Utilization: A Review from a Malaysian Perspective. | The outcomes are not focused on reducing health inequities |
| **52** | Al-Khudairy, Lena (52) | Diet, physical activity and behavioural interventions for the treatment of overweight or obese adolescents aged 12 to 17 years. | The outcomes are not focused on reducing health inequities |
| **53** | EM, Heitkemper (53) | Do health information technology self-management interventions improve glycemic control in medically underserved adults with diabetes? A systematic review and meta-analysis. | The outcomes are not focused on reducing health inequities |
| **54** | Houle-Johnson, Stephanie A (54) | Do sex differences in reported weight loss intentions and behaviours persist across demographic characteristics and weight status in youth? A systematic review. | The outcomes are not focused on reducing health inequities |
| **55** | Brody, Carinne (55) | Economic self-help group programs for improving women’s empowerment | The outcomes are not focused on reducing health inequities |
| **56** | A., Munns (56) | Effectiveness and experiences of families and support workers participating in peer-led parenting support programs delivered as home visiting programs: A comprehensive systematic review | Type of study: Narrative review |
| **57** | Hudon, C (57) | Effectiveness of case management interventions for frequent users of healthcare services: a scoping review. | Type of study: Narrative review |
| **58** | Ricci, Federico (58) | Effectiveness of occupational health and safety training: A systematic review with meta-analysis. | The outcomes are not focused on reducing health inequities |
| **59** | Walter, Helen (59) | Effectiveness of school-based family asthma educational programs in quality of life and asthma exacerbations in asthmatic children aged five to 18: a systematic review. | The outcomes are not focused on reducing health inequities |
| **60** | JP, Salerno (60) | Effectiveness of Universal School-Based Mental Health Awareness Programs Among Youth in the United States: A Systematic Review. | The outcomes are not focused on reducing health inequities |
| **61** | L., Jia (61) | Effects of drug cost sharing policy on the drug use, financial risks and moral hazard for the health insurance beneficiaries | Type of study: Póster |
| **62** | Seward, Nadine (62) | Effects of women's groups practising participatory learning and action on preventive and care-seeking behaviours to reduce neonatal mortality: A meta-analysis of cluster-randomised trials. | The outcomes are not focused on reducing health inequities |
| **63** | Burström, B (63) | Equity aspects of the Primary Health Care Choice Reform in Sweden - A scoping review | Type of study: Narrative review |
| **64** | Lehne, Gesa (64) | Equity Impact Assessment of Interventions to Promote Physical Activity among Older Adults: A Logic Model Framework. | Type of study |
| **65** | G., Lehne (65) | Equity impact assessment-Considering social inequalities in the evaluation of interventions to promote physical activity among older adults. An equity-focused review as part of the project EQUAL. | Type of study |
| **66** | Lehne, Gesa (66) | Equity impact of interventions to promote physical activity in older adults: protocol for a systematic review. | Protocol |
| **67** | Mashamba-Thompson, T P (67) | Evaluating the accessibility and utility of HIV-related point-of-care diagnostics for maternal health in rural South Africa: a study protocol. | Protocol |
| **68** | Janis Eugenio, Samira (68) | Family Health strategy: a public initiative aimed at materializing the right to health among vulnerable populations - a critical bibliographic review | Type of idiom: different from Spanish and English |
| **69** | Browne, Jennifer (69) | Food and nutrition programs for Aboriginal and Torres Strait Islander Australians: an overview of systematic reviews. | Type of study: Overview review |
| **70** | Gelormino, E (70) | From built environment to health inequalities: An explanatory framework based on evidence | Type of study |
| **71** | BJ, Angell (71) | Global systematic review of the cost-effectiveness of indigenous health interventions. | Type of study |
| **72** | Loyola-Sanchez, Adalberto (72) | Healthcare utilization for arthritis by indigenous populations of Australia, Canada, New Zealand, and the United States: A systematic review. | Type of included studies |
| **73** | Márquez-González, H (73) | Hysterectomy for the Management of Menstrual Hygiene in Women With Intellectual Disability. A Systematic Review Focusing on Standards and Ethical Considerations for Developing Countries. | The outcomes are not focused on reducing health inequities |
| **74** | SG, Pitama (74) | Implementation and impact of indigenous health curricula: a systematic review. | The outcomes are not focused on reducing health inequities |
| **75** | Hopkin, G (75) | Interventions at the Transition from Prison to the Community for Prisoners with Mental Illness: A Systematic Review. | The outcomes are not focused on reducing health inequities |
| **76** | Behera, Manas Ranjan (76) | Interventions for improved retention of skilled health workers in rural and remote areas. | The outcomes are not focused on reducing health inequities |
| **77** | Short, Megan M (77) | Interventions for motor vehicle crashes among Indigenous communities: strategies to inform Canadian initiatives. | The outcomes are not focused on reducing health inequities |
| **78** | Jenkins, Felicia (78) | Interventions Promoting Physical Activity in African American Women: An Integrative Review. | The outcomes are not focused on reducing health inequities |
| **79** | Craike, Melinda (79) | Interventions to improve physical activity among socioeconomically disadvantaged groups: an umbrella review. | Type of study |
| **80** | Clasen, Thomas F (80) | Interventions to improve water quality for preventing diarrhoea | The outcomes are not focused on reducing health inequities |
| **81** | ER, Bull (81) | Interventions to Promote Healthy Eating, Physical Activity and Smoking in Low-Income Groups: a Systematic Review with Meta-Analysis of Behavior Change Techniques and Delivery/Context. | The outcomes are not focused on reducing health inequities |
| **82** | Goudet, Sophie (82) | Interventions to tackle malnutrition and its risk factors in children living in slums: a scoping review. | The outcomes are not focused on reducing health inequities |
| **83** | Mitchell, Kevin (83) | Knowledge-related effect of interventions communicated in American Sign Language in the deaf population: A meta-analysis. | The outcomes are not focused on reducing health inequities |
| **84** | Aaron, G J (84) | Multiple-micronutrient fortified non-dairy beverage interventions reduce the risk of anemia and iron deficiency in school-aged children in low-middle income countries: A systematic review and meta-analysis(i–iv) | The outcomes are not focused on reducing health inequities |
| **85** | Burton, A E (85) | Pain Management Programmes for Non-English-Speaking Black and Minority Ethnic Groups With Long-Term or Chronic Pain. | Type of study: Narrative review |
| **86** | Busolo, David (86) | Palliative care experiences of adult cancer patients from ethnocultural groups: a qualitative systematic review protocol. | Type of study: Narrative review |
| **87** | Emami, Elham (87) | Patient satisfaction with E-Oral Health care in rural and remote settings: a systematic review protocol. | The outcomes are not focused on reducing health inequities |
| **88** | Yiu, A (88) | Patient-focused interventions to support vulnerable people using oral anticoagulants: a Type of study: Narrative review. | Type of study: Narrative review |
| **89** | MA, Pesantes (89) | Resilience in Vulnerable Populations With Type 2 Diabetes Mellitus and Hypertension: A Systematic Review and Meta-analysis. | The strategies are not focused on reducing health inequities |
| **90** | Kirkland, Laurie (90) | School-based nutrition and garden programs and parental dietary changes in low-income settings: a review. | The strategies are not focused on reducing health inequities |
| **91** | Feltner, Cynthia (91) | Screening for Intimate Partner Violence, Elder Abuse, and Abuse of Vulnerable Adults: Evidence Report and Systematic Review for the US Preventive Services Task Force. | Type of population |
| **92** | A., Andermann (92) | Screening for social determinants of health in clinical care: Moving from the margins to the mainstream | The strategies are not focused on reducing health inequities |
| **93** | Tugwell, Peter (93) | Setting priorities for knowledge translation of Cochrane reviews for health equity: Evidence for Equity. | Type of study |
| **94** | Barreto, Raissa (94) | Shares in health education for public teen: an integrative review | Type of study: Narrative review |
| **95** | Hamilton, Sandra J (95) | Smartphones in the secondary prevention of cardiovascular disease: a systematic review. | The outcomes are not focused on reducing health inequities |
| **96** | Latulippe, K (96) | Social Health Inequalities and eHealth: A Literature Review With Qualitative Synthesis of Theoretical and Empirical Studies. | Type of included studies |
| **97** | Garcia, R (97) | Specific antenatal interventions for Black, Asian and Minority Ethnic (BAME) pregnant women at high risk of poor birth outcomes in the United Kingdom: a scoping review. | Type of study: scoping review |
| **98** | Ghebre, Rahel G (98) | State-of-the-science of patient navigation as a strategy for enhancing minority clinical trial accrual. | Type of study |
| **99** | N, Mon Kyaw Soe (99) | STI Health Disparities: A Systematic Review and Meta-Analysis of the Effectiveness of Preventive Interventions in Educational Settings. | The outcomes are not focused on reducing health inequities |
| **100** | K., Canuto (100) | Strategies that target the utilization of primary health care services by Indigenous men in Australia, New Zealand, Canada and America: A comprehensive systematic review protocol | Type of included studies |
| **101** | Liljas, Ann E M (101) | Strategies to improve engagement of 'hard to reach' older people in research on health promotion: a systematic review. | Type of study: Narrative review |
| **102** | Pandian, Jeyaraj Durai (102) | Strategies to Improve Stroke Care Services in Low- and Middle-Income Countries: A Systematic Review. | The outcomes are not focused on reducing health inequities |
| **103** | Pierron, A (103) | Supporting parenting to address social inequalities in health: A synthesis of systematic reviews | Type of study: overview review |
| **104** | Smith, Melody (104) | Systematic literature review of built environment effects on physical activity and active transport - an update and new findings on health equity. | The interventions are not focused on reducing health inequities |
| **105** | K., Harris Nwanyanwu (105) | Systematic review of community-engaged research in ophthalmology | Type of study |
| **106** | Bakitas, Marie A (106) | Systematic Review of Palliative Care in the Rural Setting. | The interventions are not focused on reducing health inequities |
| **107** | Adedoyin, A Christson (107) | The Characteristics of Effective Cancer Education Media Interventions among African Americans: A Systematic Review. | The interventions are not focused on reducing health inequities |
| **108** | Tanninen, Hanna-Mari (108) | The content and effectiveness of home-based nursing interventions to promote health and well-being in families with small children: a systematic review. | Type of population |
| **109** | Marples, Owen (109) | The effect of nutrition training for health care staff on learner and patient outcomes in adults: a systematic review and meta-analysis. | The outcomes are not focused on reducing health inequities |
| **110** | Stormacq, Coraline (110) | The effectiveness of health literacy interventions on health-related outcomes among socioeconomically disadvantaged adults living in the community: a systematic review protocol. | Protocol |
| **111** | M., Vijayaraghavan (111) | The effectiveness of tobacco control policies on vulnerable populations in the USA: A review | Type of study |
| **112** | TAJ, Houweling (112) | The equity impact of community women's groups to reduce neonatal mortality: a meta-analysis of four cluster randomized trials. | Type of study |
| **113** | Pullar, Jessie (113) | The impact of poverty reduction and development interventions on non-communicable diseases and their behavioural risk factors in low and lower-middle income countries: A systematic review. | Non comparison of interventions |
| **114** | Clayton, Wendy Marie (114) | The Nurse's Role in Addressing Barriers to Cancer Screening of African Americans: An Integrative Literature Review. | Type of study |
| **115** | Schroeder, K (115) | The role and impact of community health workers in childhood obesity interventions: a systematic review and meta-analysis. | The interventions are not focused on reducing health inequities |
| **116** | AS, Thagard (116) | The role of extremes in interpregnancy interval in women at increased risk for adverse obstetric outcomes due to health disparities: A literature review | Type of included studies |
| **117** | Yu, S W Y (117) | The scope and impact of mobile health clinics in the United States: A literature review | Type of study |
| **118** | Lawrence, Briana Monique (118) | Understanding strategies that promote minority participation in breast cancer clinical trials. | The interventions are not focused on reducing health inequities |
| **119** | Smylie, J (119) | Understanding the role of Indigenous community participation in Indigenous prenatal and infant-toddler health promotion programs in Canada: A realist review | Type of study |
| **120** | Canuto, Kootsy (120) | Understanding the utilization of primary health care services by Indigenous men: a systematic review. | Type of study |
| **121** | Maden, Michelle (121) | Use of programme theory to understand the differential effects of interventions across socio-economic groups in systematic reviews-a systematic methodology review. | Type of study |
| **122** | KM, Oude Hengel (122) | Socioeconomic inequalities in reach, compliance and effectiveness of lifestyle interventions among workers: protocol for an individual participant data meta-analysis and equity-specific reanalysis. | Protocol |
| **123** | Quansah, Reginald (123) | Effectiveness of interventions to reduce indoor air pollution and/or improve health in homes using solid fuel in lower- and middle-income countries: protocol for a systematic review. | Protocol |
| **124** | Morrow, M (124) | Documenting a long-term development model in the slums of Delhi. | Type of study |
| **125** | Alicea-Alvarez, Norma (125) | A review of barriers to effective asthma management in Puerto Ricans: cultural, healthcare system and pharmacogenomic issues. | The interventions are not focused on reducing health inequities |
| **126** | Glover, Marewa (126) | A systematic review of barriers and facilitators to participation in randomized controlled trials by Indigenous people from New Zealand, Australia, Canada and the United States. | The interventions are not focused on reducing health inequities |
| **127** | Alston, Laura V (127) | A systematic review of published interventions for primary and secondary prevention of ischaemic heart disease (IHD) in rural populations of Australia. | Type of included studies |
| **128** | Morone, J (128) | A Systematic Review of Sociodemographic Representation and Cultural Responsiveness in Psychosocial and Behavioral Interventions with Adolescents with Type 1 Diabetes. | The interventions are not focused on reducing health inequities |
| **129** | Haby, Michelle M (129) | Agriculture, food, and nutrition interventions that facilitate sustainable food production and impact health: an overview of systematic reviews | Type of study: overview review |
| **130** | Utamsingh, Pooja Dushyant (130) | Beyond sensitivity. LGBT healthcare training in U.S. medical schools: A review of the literature. | Type of study |
| **131** | Moniz-Cook, E (131) | Challenge Demcare: management of challenging behaviour in dementia at home and in care homes – development, evaluation and implementation of an online individualised intervention for care homes; and a cohort study of specialist community mental health car | Type of study |
| **132** | Comrie-Thomson, Liz (132) | Challenging gender inequity through male involvement in maternal and newborn health: critical assessment of an emerging evidence base. | Type of study |
| **133** | Robertson, Francesca (133) | Challenging the generational transmission of tobacco smoking: a novel harm reduction approach in vulnerable families. | Type of study |
| **134** | Gandhi, G (134) | Charting the evolution of approaches employed by the Global Alliance for Vaccines and Immunizations (GAVI) to address inequities in access to immunization: a systematic qualitative review of GAVI policies, strategies and resource allocation mechanisms through an equity lens (1999-2014). | Type of study |
| **135** | Larson, Satu (135) | Chronic Childhood Trauma, Mental Health, Academic Achievement, and School-Based Health Center Mental Health Services. | Type of study |
| **136** | Perry, Martha (136) | Community-based interventions for improving maternal health and for reducing maternal health inequalities in high-income countries: a systematic map of research. | Type of study |
| **137** | Buscemi, Joanna (137) | Comparative Effectiveness Trial of an Obesity Prevention Intervention in EFNEP and SNAP-ED: Primary Outcomes. | Type of study |
| **138** | Duncan, K M (138) | Costs and savings of parenting interventions: results of a systematic review. | The interventions are not focused on reducing health inequities |
| **139** | Rodriguez-Fernandez, Rodrigo (139) | Current salt reduction policies across gradients of inequality-adjusted human development in the WHO European region: minding the gaps. | Type of study |
| **140** | SZ, Mabweazara (140) | Development of a context-sensitive physical activity intervention for persons living with HIV and AIDS of low socioeconomic status using the behaviour change wheel. | The interventions are not focused on reducing health inequities |
| **141** | JB, Lu (141) | Do quality improvement initiatives for diabetes care address social inequities? Secondary analysis of a systematic review. | Type of study |
| **142** | Hahn, Robert A (142) | Early Childhood Education to Promote Health Equity: A Community Guide Systematic Review. | Type of study |
| **143** | Onarheim, Kristine Husoy (143) | Economic Benefits of Investing in Women's Health: A Systematic Review. | The interventions are not focused on reducing health inequities |
| **144** | ECHEVERRI, MARGARITA (144) | Educational Interventions for Culturally Competent Healthcare: Developing a Protocol to Conduct a Systematic Review of the Rationale, Content, Teaching Methods, and Measures of Effectiveness. | Protocol |
| **145** | Kumar, G (145) | Do local enhanced services in primary care improve outcomes? Results from a literature review. | Type of study |
| **146** | Magwood, O (146) | Effectiveness of home-based records on maternal, newborn and child health outcomes: A systematic review and meta-analysis. | The interventions are not focused on reducing health inequities |
| **147** | Gibson, Odette (147) | Enablers and barriers to the implementation of primary health care interventions for Indigenous people with chronic diseases: a systematic review. | The interventions are not focused on reducing health inequities |
| **148** | Pasetto, R (148) | Environmental justice in industrially contaminated sites. A review of scientific evidence in the WHO european region | The interventions are not focused on reducing health inequities |
| **149** | Kachikis, Alisa (149) | Equity and intrapartum care by skilled birth attendant globally: protocol for a systematic review. | Protocol |
| **150** | Martinez, Omar (150) | Evaluating the impact of immigration policies on health status among undocumented immigrants: a systematic review. | The interventions are not focused on reducing health inequities |
| **151** | Sawin, Erika Metzler (151) | From Systematic Review to Call for Action : In Search of Evidence-Based Interventions to Decrease Intimate Partner Violence in Rural Hispanic American Women | The interventions are not focused on reducing health inequities |
| **152** | McFadden, Alison (152) | Gypsy, Roma and Traveller access to and engagement with health services: a systematic review. | Type of included studies |
| **153** | IL, Alcazar-Bejerano (153) | Health Behaviors, Disparities and Deterring Factors for Breast Cancer Screening of Immigrant Women - A Challenge to Health Care Professionals. | The interventions are not focused on reducing health inequities |
| **154** | McCollum, Rosalind (154) | How equitable are community health worker programmes and which programme features influence equity of community health worker services? A systematic review. | Type of included studies |
| **155** | Butler, M (155) | Improving Cultural Competence to Reduce Health Disparities | Type of study |
| **156** | Diaz, E (156) | Interventions to improve immigrant health. A scoping review. | Type of study |
| **157** | Tiwari, T (157) | Reducing Indigenous Oral Health Inequalities: A Review from 5 Nations. | The interventions are not focused on reducing health inequities (impact) |
| **158** | Pons-Vigués, M (158) | Social and health policies or interventions to tackle health inequalities in European cities: a scoping review. | The interventions are not focused on reducing health inequities (impact) |
| **159** | de Silva, Andrea M (159) | Community-based population-level interventions for promoting child oral health. | Type of study |
| **160** | Benmarhnia, Tarik (160) | Addressing equity in interventions to reduce air pollution in urban areas: a systematic review. | Type of included studies |

**Referencias**

1. Duarte GS, Delgado RM, Costa J, Vaz-Carneiro A. Analysis of the cochrane review: Non-medical prescribing versus medical prescribing for acute and chronic disease management in primary and secondary care. cochrane database syst ver. 2016;11:CD011227. Acta Med Port. 2017;30(1):7–11.

2. Guignard R, Nguyen-Thanh V, Delmer O, Lenormand MC, Blanchoz JM, Arwidson P. Interventions for smoking cessation among low socioeconomic status smokers: A literature review. Sante Publique (Paris). 2018;30(1):45–60.

3. Fekete C, Weyers S. Soziale Ungleichheit im Ernährungsverhalten: Befundlage, Ursachen und Interventionen. Bundesgesundheitsblatt - Gesundheitsforsch - Gesundheitsschutz. 2016;59(2):197–205.

4. Li WW, Lai WS. The use of telemedicine interventions to improve hypertension management among racial ethnic minorities: A systematic review. J Nurs. 2016;63(4):25–34.

5. Rozendo CA, Santos Salas A, Cameron B. A critical review of social and health inequalities in the nursing curriculum. Nurse Educ Today [Internet]. 2017;50:62–71. Available from: http://dx.doi.org/10.1016/j.nedt.2016.12.006

6. Ortiz JR, Perut M, Dumolard L, Wijesinghe PR, Jorgensen P, Ropero AM, et al. A global review of national influenza immunization policies: Analysis of the 2014 WHO/UNICEF Joint Reporting Form on immunization. Vaccine [Internet]. 2016;34(45):5400–5. Available from: http://dx.doi.org/10.1016/j.vaccine.2016.07.045

7. Gallagher RW, Polanin JR. A meta-analysis of educational interventions designed to enhance cultural competence in professional nurses and nursing students. Nurse Educ Today [Internet]. 2015;35(2):333–40. Available from: http://dx.doi.org/10.1016/j.nedt.2014.10.021

8. Mann-Jackson L, Choi D, Sutfin EL, Song EY, Foley KL, Wilkin AM, et al. A Qualitative Systematic Review of Cigarette Smoking Cessation Interventions for Persons Living with HIV. J Cancer Educ. 2019;

9. Robertson J, Stevenson L, Usher K, Devine S, Clough A. A Review of Trends in Indigenous Australian Tobacco Research (From 2004 to 2013), its Associated Outputs and Evidence of Research Translation. Nicotine Tob Res [Internet]. 2015 Aug 15;17(8):1039–48. Available from: https://academic.oup.com/ntr/article-lookup/doi/10.1093/ntr/ntv018

10. Yehia F, Nahas Z, Saleh S. A roadmap to parity in mental health financing: the case of Lebanon. J Ment Health Policy Econ. 2014;17(3):131–41.

11. Farmer J, Kenny A, McKinstry C, Huysmans RD. A scoping review of the association between rural medical education and rural practice location. Hum Resour Health. 2015;13(1).

12. Lopez-Class M, Peprah E, Zhang X, Kaufmann PG, Engelgau MM. A strategic framework for utilizing late-stage (t4) translation research to address health inequities. Ethn Dis. 2016;26(3):387–94.

13. Driscoll S. A SYSTEMATIC REVIEW AND QUANTITATIVE META-ANALYSIS OF THE ACCURACY OF VISUAL INSPECTION FOR CERVICAL CANCER SCREENING: DOES PROVIDER TYPE OR TRAINING MATTER? 2016.

14. De Dassel JL, Ralph AP, Cass A. A systematic review of adherence in Indigenous Australians: An opportunity to improve chronic condition management. BMC Health Serv Res. 2017;17(1):1–13.

15. Han HR, McKenna S, Nkimbeng M, Wilson P, Rives S, Ajomagberin O, et al. A Systematic Review of Community Health Center Based Interventions for People with Diabetes. J Community Health [Internet]. 2019; Available from: https://doi.org/10.1007/s10900-019-00693-y

16. Brooker K, Van Dooren K, McPherson L, Lennox N, Ware R. Systematic review of interventions aiming to improve involvement in physical activity among adults with intellectual disability. J Phys Act Heal. 2015;12(3):434–44.

17. Conrad E, Knowlden AP. A systematic review of obesity interventions targeting anthropometric changes in youth with intellectual disabilities. J Intellect Disabil. 2018;174462951879691.

18. Pelletier CA, Smith-Forrester J, Klassen-Ross T. A systematic review of physical activity interventions to improve physical fitness and health outcomes among Indigenous adults living in Canada. Prev Med Reports [Internet]. 2017;8(July):242–9. Available from: https://doi.org/10.1016/j.pmedr.2017.11.002

19. Stangl AL, Singh D, Windle M, Sievwright K, Footer K, Iovita A, et al. A systematic review of selected human rights programs to improve HIV-related outcomes from 2003 to 2015: what do we know? BMC Infect Dis. 2019;19(1).

20. Newton RL, Griffith DM, Kearney WB, Bennett GG. A systematic review of weight loss, physical activity and dietary interventions involving African American men. Obes Rev. 2014;15(October):93–106.

21. Pressick EL, Gray MA, Cole RL, Burkett BJ. A systematic review on research into the effectiveness of group-based sport and exercise programs designed for Indigenous adults. J Sci Med Sport [Internet]. 2016;19(9):726–32. Available from: http://dx.doi.org/10.1016/j.jsams.2015.11.005

22. Johri M, Verguet S, Morris SK, Sharma JK, Ram U, Gauvreau C, et al. Adding interventions to mass measles vaccinations in India. Bull World Health Organ. 2016;94(10):718–27.

23. Lee J, Schram A, Riley E, Harris P, Baum F, Fisher M, et al. Addressing health equity through action on the social determinants of health: A global review of policy outcome evaluation methods. Int J Heal Policy Manag [Internet]. 2018;7(7):581–92. Available from: https://doi.org/10.15171/ijhpm.2018.04

24. Farrer L, Marinetti C, Cavaco YK, Costongs C. Advocacy for health equity: A synthesis review. Milbank Q. 2015;93(2):392–437.

25. Chopel A. An Ecological Community-Based Participatory Research Study of Late Diagnosed HIV/AIDS in Oakland, California: Investigating influential factors in racial/ ethnic health inequities. University of California, Berkeley; 2014.

26. Bottorff JL, Seaton CL, Johnson ST, Caperchione CM, Oliffe JL, More K, et al. An Updated Review of Interventions that Include Promotion of Physical Activity for Adult Men. Sport Med. 2015;45(6):775–800.

27. Brown FL, de Graaff AM, Annan J, Betancourt TS. Annual Research Review: Breaking cycles of violence – a systematic review and common practice elements analysis of psychosocial interventions for children and youth affected by armed conflict. J Child Psychol Psychiatry Allied Discip. 2017;58(4):507–24.

28. Hatzenbuehler ML, Flores JE, Cavanaugh JE, Onwuachi-Willig A, Ramirez MR. Anti-bullying Policies and Disparities in Bullying: A State-Level Analysis. Am J Prev Med [Internet]. 2017;53(2):184–91. Available from: http://dx.doi.org/10.1016/j.amepre.2017.02.004

29. Koller TS, Saint V, Floranita R, Koemara Sakti GM, Pambudi I, Hermawan L, et al. Applying the Innov8 approach for reviewing national health programmes to leave no one behind: lessons learnt from Indonesia. Glob Health Action [Internet]. 2018;11(1):3–9. Available from: https://doi.org/10.1080/16549716.2018.1423744

30. Mawn L, Oliver EJ, Akhter N, Bambra CL, Torgerson C, Bridle C, et al. Are we failing young people not in employment, education or training (NEETs)? A systematic review and meta-analysis of re-engagement interventions. Syst Rev [Internet]. 2017;6(1):1–17. Available from: http://dx.doi.org/10.1186/s13643-016-0394-2

31. Njoroge M, Zurovac D, Ogara EAA, Chuma J, Kirigia D. Assessing the feasibility of eHealth and mHealth: A systematic review and analysis of initiatives implemented in Kenya. BMC Res Notes. 2017;10(1):1–11.

32. Farnbach S, Eades AM, Hackett ML. Australian Aboriginal and Torres Strait Islander-focused primary healthcare social and emotional wellbeing research: A systematic review protocol. Syst Rev [Internet]. 2015;4(1):2–7. Available from: http://dx.doi.org/10.1186/s13643-015-0180-6

33. Rice K, Te Hiwi B, Zwarenstein M, Lavallee B, Barre DE, Harris SB. Best Practices for the Prevention and Management of Diabetes and Obesity-Related Chronic Disease among Indigenous Peoples in Canada: A Review. Can J Diabetes [Internet]. 2016;40(3):216–25. Available from: http://dx.doi.org/10.1016/j.jcjd.2015.10.007

34. Rayner G, Crossen K. Can a paediatric department provide health care for vulnerable adolescents? N Z Med J [Internet]. 2014 Jul 18;127(1398):67–76. Available from: http://www.ncbi.nlm.nih.gov/pubmed/25146862

35. van Urk F, Brown T, Waller R, Mayo-Wilson E. Centre-based day care for children younger than five years of age in high-income countries ( Review ). Cochrane Database Syst Rev. 2014;(9).

36. Tsuji H, Usuda K, Takahashi Y, Kono K, Tamaki J. Challenges and solutions in immigrant occupational health in the United States: a literature review and comparative analysis. Sangyo Eiseigaku Zasshi [Internet]. 2016;58(2):63–71. Available from: https://www.jstage.jst.go.jp/article/sangyoeisei/58/2/58_E15005/_article/-char/ja/

37. Islam NS, Zanowiak JM, Riley L, Nadkarni SK, Kwon SC, Trinh-Shevrin C. Characteristics of Asian American, Native Hawaiian, and Pacific Islander Community Health Worker Programs: A Systematic Review. J Health Care Poor Underserved [Internet]. 2015;26(2A):238–68. Available from: https://muse.jhu.edu/content/crossref/journals/journal_of_health_care_for_the_poor_and_underserved/v026/26.2A.islam.html

38. Cheng TL, Emmanuel MA, Levy DJ, Jenkins RR. Child health disparities: What can a clinician do? Pediatrics. 2015;136(5):962–8.

39. Lauer N. Childhood Healthy Behaviors Intervention in a Pediatric Primary Care Setting: Impact on Provider Practice. The Catholic University of America In; 2014.

40. Killian-Farrell C. Childhood trauma and adolescent mental health: A transdisciplinary approach for social work research and practice. The University of North Carolina at Chapel Hill; 2016.

41. Racine AD, Long TF, Helm ME, Hudak M, Shenkin BN, Snider IG, et al. Children’s Health Insurance Program (CHIP): Accomplishments, challenges, and policy recommendations. Pediatrics. 2014;133(3).

42. Carethers JM. Clinical and genetic factors to inform reducing colorectal cancer disparitites in African Americans. Front Oncol. 2018;8(NOV):4–10.

43. León Cabrera P, Pría Barro M del C, Perdomo Victoria I. Cobertura y acceso a los servicios de salud para el abordaje de la tuberculosis. Rev Cuba Salud Pública [Internet]. 2018;44(4):186–99. Available from: http://www.scielosp.org/scielo.php?script=sci_arttext&pid=S0864-34662018000400186&lang=es%0Ahttp://www.scielosp.org/pdf/rcsp/v44n4/1561-3127-rcsp-44-04-186.pdf

44. Cooper LA. Commentary: Training and mentoring the next generation of health equity researchers: Insights from the field. Ethn Dis. 2018;28(4):579–85.

45. Schiavo R, May Leung M, Brown M. Communicating risk and promoting disease mitigation measures in epidemics and emerging disease settings. Pathog Glob Health [Internet]. 2014;108(2):76–94. Available from: http://www.scopus.com/inward/record.url?eid=2-s2.0-84897875907&partnerID=40&md5=47a8e24f8b357c4816de001e796f2359

46. McFadden A, Atkin K, Bell K, Innes N, Jackson C, Jones H, et al. Community engagement to enhance trust between Gypsy/Travellers, and maternity, early years’ and child dental health services: protocol for a multi-method exploratory study. Int J Equity Health [Internet]. 2016;15(1):1–9. Available from: http://dx.doi.org/10.1186/s12939-016-0475-9

47. Eslava-Schmalbach J, Garzón-Orjuela N, Elias V, Reveiz L, Tran N, Langlois E V. Conceptual framework of equity-focused implementation research for health programs (EquIR). Int J Equity Health [Internet]. 2019 Dec 31;18(1):80. Available from: https://equityhealthj.biomedcentral.com/articles/10.1186/s12939-019-0984-4

48. Lyman DR, Braude L, George P, Dougherty RH, Daniels AS, Ghose SS, et al. Consumer and family psychoeducation: Assessing the evidence. Psychiatr Serv. 2014;65(4):416–28.

49. Orkin AM, McArthur A, Venugopal J, Kithulegoda N, Martiniuk A, Buchman DZ, et al. Defining and measuring health equity in research on task shifting in high-income countries: A systematic review. SSM - Popul Heal [Internet]. 2019;7(October 2018):100366. Available from: https://doi.org/10.1016/j.ssmph.2019.100366

50. Mazarello Paes V, Hesketh K, O’Malley C, Moore H, Summerbell C, Griffin S, et al. Determinants of sugar-sweetened beverage consumption in young children: A systematic review. Obes Rev. 2015;16(11):903–13.

51. Manaf MRA, Surendra NK, Gafor AHA, Hooi LS, Bavanandan S. Dialysis Provision and Implications of Health Economics on Peritoneal Dialysis Utilization: A Review from a Malaysian Perspective. Int J Nephrol. 2017;2017.

52. Loveman E, Colquitt JL, Mead E, Johnson RE, Fraser H, Olajide J, et al. Diet , physical activity and behavioural interventions for the treatment of overweight or obese adolescents aged 12 to 17 years ( Review ). Cochrane [Internet]. 2017;2016(6):CD012105. Available from: http://as.wiley.com/WileyCDA/Brand/id-6.html%0Ahttp://ovidsp.ovid.com/ovidweb.cgi?T=JS&PAGE=reference&D=emexa&NEWS=N&AN=609169090

53. Heitkemper EM, Mamykina L, Travers J, Smaldone A. Do health information technology self-management interventions improve glycemic control in medically underserved adults with diabetes? A systematic review and meta-analysis. J Am Med Informatics Assoc. 2017;24(5):1024–35.

54. Houle-Johnson SA, Kakinami L. Do sex differences in reported weight loss intentions and behaviours persist across demographic characteristics and weight status in youth? A systematic review. BMC Public Health. 2018;18(1):1–17.

55. Brody C, de Hoop T, Vojtkova M, Warnock R, Dunbar M, Murthy P, et al. Economic Self-Help group Programs for Improving Women’s Empowerment: A Systematic Review. Campbell Syst Rev. 2015;11(1):1–182.

56. Munns A, Watts R, Hegney D, Walker R. Effectiveness and experiences of families and support workers participating in peer-led parenting support programs delivered as home visiting programs. JBI Database Syst Rev Implement Reports [Internet]. 2016 Oct;14(10):167–208. Available from: https://insights.ovid.com/crossref?an=01938924-201610000-00016

57. Hudon C, Chouinard M, Lambert M, Dufour I, Krieg C. Effectiveness of case management interventions for frequent users of healthcare services : a scoping review. 2016;(Cm).

58. Ricci F, Chiesi A, Bisio C. Effectiveness of occupational health and safety training A systematic review with meta-analysis. 2016;28(6):355–77.

59. Walter H, Rose FS, Doreen U, Aileen C, Singleton J. Effectiveness of school-based family asthma educational programs on quality of life and asthma exacerbations in asthmatic children aged five to 18 : a systematic review. 2016;113–38.

60. Salerno JP. Effectiveness of Universal School-Based Mental Health Awareness Programs Among Youth in the United States: A Systematic Review. J Sch Health [Internet]. 2016 Dec;86(12):922–31. Available from: http://doi.wiley.com/10.1111/josh.12461

61. Jia L, Meng Q, Yuan B, Fang L. Effects of Drug Cost Sharing Policy on the Drug Use, Financial Risks and Moral Hazard for the Health Insurance Beneficiaries. Value Heal [Internet]. 2014 Nov;17(7):A795. Available from: https://linkinghub.elsevier.com/retrieve/pii/S1098301514023894

62. Seward N, Neuman M, Colbourn T, Osrin D, Lewycka S, Azad K, et al. Effects of women ’ s groups practising participatory learning and action on preventive and care-seeking behaviours to reduce neonatal mortality : A meta-analysis of cluster-randomised trials. 2017;1–22.

63. Burström B, Burström K, Nilsson G, Tomson G, Whitehead M, Winblad U. Equity aspects of the Primary Health Care Choice Reform in Sweden – a scoping review. 2017;1–10.

64. Lehne G, Voelcker-rehage C, Meyer J, Bammann K, Gansefort D, Brüchert T, et al. Equity Impact Assessment of Interventions to Promote Physical Activity among Older Adults : A Logic Model Framework.

65. Lehne G, Brand T, Bolte G. Equity impact assessment-Considering social inequalities in the evaluation of interventions to promote physical activity among older adults. An equity-focused review as part of the project EQUAL. [Internet]. HEC 2016. Health – Exploring Complexity: An Interdisciplinary Approach. 2016 [cited 2019 Aug 19]. Available from: https://www.ipp.uni-bremen.de/projects/en/?publ=6293&proj=593

66. Lehne G, Bolte G. Equity impact of interventions to promote physical activity in older adults : protocol for a systematic review. Syst Rev [Internet]. 2016;1–7. Available from: http://dx.doi.org/10.1186/s13643-016-0194-8

67. Drain PK, Sartorius B. Evaluating the accessibility and utility of HIV-related point-of-care diagnostics for maternal health in rural South Africa : a study protocol. 2016;

68. Eugenio SJ, Arena Ventura CA. Estratégia saúde da família: iniciativa pública destinada a populações vulneráveis para garantia do direto à saúde - uma revisão crítica da literatura. Cad IBERO-AMERICANOS DIREITO SANITÁRIO [Internet]. 2017 Sep 29;6(3):129. Available from: http://www.cadernos.prodisa.fiocruz.br/index.php/cadernos/article/view/402

69. Mph JB, Adams K, Hayes R, Associate A. Food and nutrition programs for Aboriginal and Torres Strait Islander Australians : an overview of systematic reviews. (July 2017).

70. Gelormino E, Melis G, Marietta C, Costa G. From built environment to health inequalities : An explanatory framework based on evidence. PMEDR [Internet]. 2015;2:737–45. Available from: http://dx.doi.org/10.1016/j.pmedr.2015.08.019

71. Angell BJ, Muhunthan J, Irving M, Eades S, Jan S. Global Systematic Review of the Cost-Effectiveness of Indigenous Health Interventions. 2014;9(11).

72. Loyola-Sanchez A, Hurd K, Barnabe C. Healthcare utilization for arthritis by indigenous populations of Australia, Canada, New Zealand, and the United States: A systematic review ☆. Semin Arthritis Rheum [Internet]. 2017 Apr;46(5):665–74. Available from: http://www.embase.com/search/results?subaction=viewrecord&from=export&id=L613889334%0Ahttp://dx.doi.org/10.1002/art.39977

73. Márquez-González H, Valdez-Martinez E, Bedolla M. Hysterectomy for the management of menstrual hygiene in women with intellectual disability. A systematic review focusing on standards and ethical considerations for developing countries. Front Public Heal. 2018;6(NOV).

74. Pitama SG, Palmer SC, Huria T, Lacey C, Wilkinson T. Implementation and impact of indigenous health curricula: a systematic review. Med Educ. 2018;52(9):898–909.

75. Hopkin G, Evans-Lacko S, Forrester A, Shaw J, Thornicroft G. Interventions at the Transition from Prison to the Community for Prisoners with Mental Illness: A Systematic Review. Adm Policy Ment Heal Ment Heal Serv Res. 2018;45(4):623–34.

76. Behera MR, Prutipinyo C, Sirichotiratana N, Viwatwongkasem C. Interventions for improved retention of skilled health workers in rural and remote areas. Ann Trop Med Public Heal. 2017;10(1):16.

77. Short MM, Mushquash CJ, Bédard M. Interventions for motor vehicle crashes among Indigenous communities: Strategies to inform Canadian initiatives. Can J Public Heal. 2014;105(4).

78. Jenkins F, Jenkins C, Gregoski MJ, Magwood GS. Interventions Promoting Physical Activity in African American Women. J Cardiovasc Nurs [Internet]. 2017 Jun 21;32(1):22–9. Available from: http://www.nature.com/articles/nature22814

79. M. C, G. W, T.A. H, E.G. B. Interventions to improve physical activity among socioeconomically disadvantaged groups: An umbrella review. Int J Behav Nutr Phys Act [Internet]. 2018;15(1):1–11. Available from: http://www.embase.com/search/results?subaction=viewrecord&from=export&id=L622136605%0Ahttp://dx.doi.org/10.1186/s12966-018-0676-2

80. Clasen TTF, Alexander KTK, Sinclair D, Boisson S, Peletz R, Chang HH, et al. Interventions to improve water quality for preventing diarrhoea ( Review ) SUMMARY OF FINDINGS FOR THE MAIN COMPARISON. Cochrane Libr [Internet]. 2015;2015(10):CD004794. Available from: http://www.embase.com/search/results?subaction=viewrecord&from=export&id=L620549078%0Ahttp://dx.doi.org/10.1002/14651858.CD004794.pub3%0Ahttp://mgetit.lib.umich.edu/sfx_locater?sid=EMBASE&issn=1469493X&id=doi:10.1002%2F14651858.CD004794.pub3&atitle=Interv

81. Bull ER, McCleary N, Li X, Dombrowski SU, Dusseldorp E, Johnston M. Interventions to Promote Healthy Eating, Physical Activity and Smoking in Low-Income Groups: a Systematic Review with Meta-Analysis of Behavior Change Techniques and Delivery/Context. Int J Behav Med. 2018;25(6):605–16.

82. Goudet S, Griffiths P, Bogin B, Madise N. Interventions to tackle malnutrition and its risk factors in children living in slums: a scoping review. Ann Hum Biol. 2017;44(1):1–10.

83. Mitchell K. Knowledge-related effect of interventions communicated in American Sign Language in the deaf population: A meta-analysis. [Internet]. Vol. 160, American Annals of the Deaf. 2015. Available from: https://muse.jhu.edu/content/crossref/journals/american_annals_of_the_deaf/v160/160.2.article04.html

84. Aaron GJ, Dror DK, Yang Z. Multiple-micronutrient fortified non-dairy beverage interventions reduce the risk of anemia and iron deficiency in school-aged children in low-middle income countries: A systematic review and meta-analysis(i–iv). Nutrients. 2015;7(5):3847–68.

85. Burton AE, Shaw RL. Pain Management Programmes for Non-English-Speaking Black and Minority Ethnic Groups With Long-Term or Chronic Pain. Musculoskeletal Care. 2015;13(4):187–203.

86. Busolo D, Woodgate R. Palliative care experiences of adult cancer patients from ethnocultural groups: a qualitative systematic review protocol. JBI database Syst Rev Implement reports. 2015;13(1):99–111.

87. Emami E, Kadoch N, Homayounfar S, Harnagea H, Dupont P, Giraudeau N, et al. Patient satisfaction with E-Oral Health care in rural and remote settings: A systematic review protocol. Syst Rev. 2017;6(1):1–6.

88. Yiu A, Bajorek B. Patient-focused interventions to support vulnerable people using oral anticoagulants: a Type of study: Narrative review. Ther Adv Drug Saf [Internet]. 2019 Jan 13;10(2s):204209861984742. Available from: http://journals.sagepub.com/doi/10.1177/2042098619847423

89. Pesantes MA, Lazo-Porras M, Abu Dabrh AM, Avila-Ramirez J.R., Caycho M, Villamonte G.Y., et al. Resilience in vulnerable populations with type 2 dm and hypertension: Review and Meta-Analysis. Can J Cardiol. 2015;31(9):1180–8.

90. Kirkland L, Apatu E, Mease A, Largo-Wight E. School-based nutrition and garden programs and parental dietary changes in low-income settings: a review. Int J Heal Promot Educ [Internet]. 2018;56(4–5):226–36. Available from: https://doi.org/10.1080/14635240.2018.1512881

91. Feltner C, Wallace I, Berkman N, Kistler CE, Middleton JC, Barclay C, et al. Screening for Intimate Partner Violence, Elder Abuse, and Abuse of Vulnerable Adults: Evidence Report and Systematic Review for the US Preventive Services Task Force. JAMA - J Am Med Assoc. 2018;320(16):1688–701.

92. Andermann A. Screening for social determinants of health in clinical care: Moving from the margins to the mainstream. Public Health Rev. 2018;39(1):1–17.

93. Tugwell P, Petkovic J, Welch V, Vincent J, Bhutta ZA, Churchill R, et al. Setting priorities for knowledge translation of Cochrane reviews for health equity: Evidence for Equity. Int J Equity Health. 2017;16(1):1–8.

94. Mont R, Barreto A, Suelen A, Cavalcante P. AÇÕES EDUCATIVAS EM SAÚDE PARA D PÚDLICD ADOLESCENTE: UMA REVISÃO INTEGRATIVA. Rev APS. 2016;19(2):277–85.

95. Hamilton SJ, Mills B, Birch EM, Thompson SC. Smartphones in the secondary prevention of cardiovascular disease: A systematic review. BMC Cardiovasc Disord. 2018;18(1):1–23.

96. Latulippe K, Hamel C, Giroux D. Social health inequalities and eHealth: A literature review with qualitative synthesis of theoretical and empirical studies. J Med Internet Res. 2017;19(4):1–14.

97. Garcia R, Ali N, Papadopoulos C, Randhawa G. Specific antenatal interventions for Black, Asian and Minority Ethnic (BAME) pregnant women at high risk of poor birth outcomes in the United Kingdom: A scoping review. BMC Pregnancy Childbirth [Internet]. 2015;15(1):1–13. Available from: http://dx.doi.org/10.1186/s12884-015-0657-2

98. Ghebre RG, Jones LA, Wenzel JA, Martin MY, Durant RW, Ford JG. State-of-the-science of patient navigation as a strategy for enhancing minority clinical trial accrual. Cancer [Internet]. 2014 Apr 1;120(1):1122–30. Available from: http://doi.wiley.com/10.1002/cncr.28570

99. Soe NMK, Bird Y, Schwandt M, Moraros J. STI health disparities: A systematic review and meta-analysis of the effectiveness of preventive interventions in educational settings. Int J Environ Res Public Health. 2018;15(12).

100. Canuto K, Harfield S, Brown A, Wittert G. Strategies that target the utilization of primary health care services by Indigenous men in Australia, New Zealand, Canada and America: a comprehensive systematic review protocol. JBI Database Syst Rev Implement Reports. 2015;13(9):95.

101. Liljas AEM, Walters K, Jovicic A, Iliffe S, Manthorpe J, Goodman C, et al. Strategies to improve engagement of ‘hard to reach’ older people in research on health promotion: a systematic review. BMC Public Health. 2017;17(1):1–12.

102. William AG, Kate MP, Norrving B, Mensah GA, Davis S, Roth GA, et al. Strategies to Improve Stroke Care Services in Low- and Middle-Income Countries: A Systematic Review. Neuroepidemiology. 2017;49(1–2):45–61.

103. Pierron A, Fond-Harmant L, Laurent A, Alla F. Supporting parenting to address social inequalities in health: A synthesis of systematic reviews. BMC Public Health. 2018;18(1).

104. Smith M, Hosking J, Woodward A, Witten K, MacMillan A, Field A, et al. Systematic literature review of built environment effects on physical activity and active transport - an update and new findings on health equity. Int J Behav Nutr Phys Act. 2017;14(1):1–27.

105. Harris Nwanyanwu K, Grossetta Nardini HK, Shaughness G, Nunez-Smith M, Newman-Casey P-A. Systematic review of community-engaged research in ophthalmology. Expert Rev Ophthalmol [Internet]. 2017 May 4;12(3):233–41. Available from: https://www.tandfonline.com/doi/full/10.1080/17469899.2017.1311787

106. Bakitas MA, Elk R, Astin M, Ceronsky L, Clifford KN, Nicholas Dionne-Odom J, et al. Systematic review of palliative care in the rural setting. Cancer Control. 2015;22(4):450–64.

107. Adedoyin AC, Sherr ME, Adedoyin OO, Royse DD, Jackson MS, Adu-Boahene AB. The characteristics of effective cancer education media interventions among African Americans: A systematic review. J Evidence-Informed Soc Work. 2016;13(4):331–44.

108. Tanninen HM, Häggman-Laitila A, Pietilä AM, Kangasniemi M. The content and effectiveness of home-based nursing interventions to promote health and well-being in families with small children: A systematic review. Scand J Caring Sci. 2016;30(2):217–33.

109. Marples O, Baldwin C, Weekes CE. The effect of nutrition training for health care staff on learner and patient outcomes in adults: A systematic review and meta-analysis. Am J Clin Nutr. 2017;106(1):284–310.

110. Stormacq C, Wosinski J, van den Broucke S. The effectiveness of health literacy interventions on health-related outcomes among socioeconomically disadvantaged adults living in the community: A systematic review protocol. JBI Database Syst Rev Implement Reports. 2016;14(2):49–63.

111. Vijayaraghavan M, Schroeder SA, Kushel M. The effectiveness of tobacco control policies on vulnerable populations in the USA: A review. Postgrad Med J. 2016;92(1093):670–6.

112. Houweling TAJ, Looman CWN, Azad K, Das S, King C, Kuddus A, et al. The equity impact of community women’s groups to reduce neonatal mortality: A meta-analysis of four cluster randomized trials. Int J Epidemiol. 2019;48(1):168–82.

113. Pullar J, Allen L, Townsend N, Williams J, Foster C, Roberts N, et al. The impact of poverty reduction and development interventions on noncommunicable diseases and their behavioural risk factors in low and lower-middle income countries: A systematic review. PLoS One. 2018;13(2).

114. Clayton WM, Tariman JD. The Nurse’s Role in Addressing Barriers to Cancer Screening of African Americans: An Integrative Literature Review. J Nurs Pract Appl Rev Res. 2009;

115. Schroeder K, McCormick R, Perez A, Lipman TH. The role and impact of community health workers in childhood obesity interventions: a systematic review and meta-analysis. Obes Rev. 2018;19(10):1371–84.

116. Thagard AS, Napolitano PG, Bryant AS. The Role of Extremes in Interpregnancy Interval in Women at Increased Risk for Adverse Obstetric Outcomes Due to Health Disparities: A Literature Review. Curr Women s Heal Rev [Internet]. 2018 Jul 3;14(3):242–50. Available from: http://www.eurekaselect.com/151101/article

117. Yu SWY, Hill C, Ricks ML, Bennet J, Oriol NE. The scope and impact of mobile health clinics in the United States: A literature review. Int J Equity Health. 2017;16(1):1–12.

118. Lawrence B. UNDERSTANDING STRATEGIES THAT PROMOTE MINORITY PARTICIPATION IN BREAST CANCER CLINICAL TRIALS. 2013.

119. Smylie J, Kirst M, McShane K, Firestone M, Wolfe S, O’Campo P. Understanding the role of Indigenous community participation in Indigenous prenatal and infant-toddler health promotion programs in Canada: A realist review. Soc Sci Med [Internet]. 2016;150:128–43. Available from: http://dx.doi.org/10.1016/j.socscimed.2015.12.019

120. Canuto K, Brown A, Wittert G, Harfield S. Understanding the utilization of primary health care services by Indigenous men: A systematic review 11 Medical and Health Sciences 1117 Public Health and Health Services. BMC Public Health. 2018;18(1):1–12.

121. Maden M, Cunliffe A, McMahon N, Booth A, Carey GM, Paisley S, et al. Use of programme theory to understand the differential effects of interventions across socio-economic groups in systematic reviews-a systematic methodology review. Syst Rev. 2017;6(1):1–23.

122. Oude Hengel KM, Coenen P, Robroek SJW, Boot CRL, Van Der Beek AJ, Van Lenthe FJ, et al. Socioeconomic inequalities in reach, compliance and effectiveness of lifestyle interventions among workers: Protocol for an individual participant data meta-analysis and equity-specific reanalysis. BMJ Open. 2019;9(2).

123. Quansah R, Ochieng CA, Semple S, Juvekar S, Emina J, Armah FA, et al. Effectiveness of interventions to reduce indoor air pollution and / or improve health in homes using solid fuel in lower and middle income countries : protocol for a systematic review. 2015;1–5.

124. Morrow M, Armstrong G, Dayal P, Kermode M. Documenting a long-term development model in the slums of Delhi. BMC Int Health Hum Rights [Internet]. 2016;16(1):1–11. Available from: http://dx.doi.org/10.1186/s12914-016-0088-9

125. Alicea-Alvarez N, Swanson-Biearman B, Kelsen SG. A review of barriers to effective asthma management in Puerto Ricans: Cultural, healthcare system and pharmacogenomic issues. J Asthma. 2014;51(1):97–105.

126. Glover M, Kira A, Johnston V, Walker N, Thomas D, Chang AB, et al. A systematic review of barriers and facilitators to participation in randomized controlled trials by Indigenous people from New Zealand, Australia, Canada and the United States. Glob Health Promot. 2015;22(1):21–31.

127. Alston L V., Peterson KL, Jacobs JP, Allender S, Nichols M. A systematic review of published interventions for primary and secondary prevention of ischaemic heart disease (IHD) in rural populations of Australia. BMC Public Health [Internet]. 2016;16(1). Available from: http://dx.doi.org/10.1186/s12889-016-3548-1

128. Morone J. Systematic review of sociodemographic representation and cultural responsiveness in psychosocial and behavioral interventions with adolescents with type 1 diabetes. J Diabetes. 2019;11(7):582–92.

129. Haby MM, Chapman E, Clark R, Galvão LAC. Agriculture, food, and nutrition interventions that facilitate sustainable food production and impact health: an overview of systematic reviews TT - Intervenciones agropecuarias, alimentarias y nutricionales que favorecen la producción sostenible de alim. Rev Panam Salud Publica [Internet]. 2016;40(1):48–56. Available from: http://www.scielosp.org/scielo.php?script=sci_arttext&pid=S1020-49892016000700048

130. Utamsingh PD, Kenya S, Lebron CN, Carrasquillo O. Beyond Sensitivity. LGBT Healthcare Training in U.S. Medical Schools: A Review of the Literature. Am J Sex Educ [Internet]. 2017;12(2):148–69. Available from: https://doi.org/10.1080/15546128.2017.1298070

131. Moniz-Cook E, Hart C, Woods B, Whitaker C, James I, Russell I, et al. Challenge Demcare: management of challenging behaviour in dementia at home and in care homes – development, evaluation and implementation of an online individualised intervention for care homes; and a cohort study of specialist community mental health car. Program Grants Appl Res. 2017;5(15):1–290.

132. Comrie-Thomson L, Tokhi M, Ampt F, Portela A, Chersich M, Khanna R, et al. Challenging gender inequity through male involvement in maternal and newborn health: critical assessment of an emerging evidence base. Cult Heal Sex [Internet]. 2015;17:S177–89. Available from: http://dx.doi.org/10.1080/13691058.2015.1053412

133. Robertson F. Challenging the generational transmission of tobacco smoking: a novel harm reduction approach in vulnerable families. Child Fam Soc Work. 2017;22(1):106–15.

134. Gandhi G. Charting the evolution of approaches employed by the Global Alliance for Vaccines and Immunizations (GAVI) to address inequities in access to immunization: A systematic qualitative review of GAVI policies, strategies and resource allocation mechanisms thr. BMC Public Health [Internet]. 2015;15(1). Available from: http://dx.doi.org/10.1186/s12889-015-2521-8

135. Larson S, Chapman S, Spetz J, Brindis CD. Chronic Childhood Trauma, Mental Health, Academic Achievement, and School-Based Health Center Mental Health Services. J Sch Health. 2017;87(9):675–86.

136. Perry M, Becerra F, Kavanagh J, Serre A, Vargas E, Becerril V. Community-based interventions for improving maternal health and for reducing maternal health inequalities in high-income countries: A systematic map of research. Global Health. 2015;10(1):1–12.

137. Buscemi J, Odoms-Young A, Stolley MR, Schiffer L, Blumstein L, Clark MH, et al. Comparative effectiveness trial of an obesity prevention intervention in EFNEP and SNAP-ED: Primary outcomes. Nutrients. 2019;11(5).

138. Duncan KM, MacGillivray S, Renfrew MJ. Costs and savings of parenting interventions: results of a systematic review. Child Care Health Dev. 2017;43(6):797–811.

139. Rodriguez-Fernandez R, Siopa M, Simpson SJ, Amiya RM, Breda J, Cappuccio FP. Current salt reduction policies across gradients of inequality-adjusted human development in the WHO European region: minding the gaps. Public Health Nutr [Internet]. 2014 Aug 8;17(8):1894–904. Available from: https://www.cambridge.org/core/product/identifier/S136898001300195X/type/journal_article

140. Mabweazara SZ, Leach LL, Ley C. Development of a context-sensitive physical activity intervention for persons living with HIV and AIDS of low socioeconomic status using the behaviour change wheel. BMC Public Health. 2019;19(1):1–16.

141. Lu JB, Danko KJ, Elfassy MD, Welch V, Grimshaw JM, Ivers NM. Do quality improvement initiatives for diabetes care address social inequities? Secondary analysis of a systematic review. BMJ Open. 2018;8(2):1–7.

142. Hahn RA, Barnett WS, Knopf JA, Truman BI, Johnson RL, Fielding JE, et al. Early Childhood Education to Promote Health Equity: A Community Guide Systematic Review. J Public Heal Manag Pract [Internet]. 2016;22(5):E1–8. Available from: https://insights.ovid.com/crossref?an=00005082-201407000-00005

143. Onarheim KH, Iversen JH, Bloom DE. Economic benefits of investing in women’s health: A systematic review. PLoS One. 2016;11(3):1–23.

144. ECHEVERRI M, CHEN AMH. Educational Interventions for Culturally Competent Healthcare: Developing a Protocol to Conduct a Systematic Review of the Rationale, Content, Teaching Methods, and Measures of Effectiveness. J Best Pract Heal Prof Divers Educ Res Policy [Internet]. 2016;9(1):1160–77. Available from: http://offcampus.lib.washington.edu/login?url=http://search.ebscohost.com/login.aspx?direct=true&db=bth&AN=122513022&site=ehost-live

145. Kumar G, Quigley J, Singh M, Keeping S, Pitman R, Carroll S. Do local enhanced services in primary care improve outcomes? Results from a literature review. Qual Prim Care. 2014;22(3):157–69.

146. Id KT, Oliver S, Magwood O, Kpade V, Mayhew D, Pottie K. Effectiveness of home-based records on maternal , newborn and child health outcomes : A systematic review and meta- analysis. 2019;1–17.

147. Gibson O, Lisy K, Davy C, Aromataris E, Kite E, Lockwood C, et al. Enablers and barriers to the implementation of primary health care interventions for Indigenous people with chronic diseases : a systematic review. Implement Sci [Internet]. 2015;1–11. Available from: http://dx.doi.org/10.1186/s13012-015-0261-x

148. Pasetto R, Mattioli B, Marsili D. Environmental Justice in Industrially Contaminated Sites . A Review of Scientific Evidence in the WHO European Region. 2019;

149. Kachikis A, Moller A, Allen T, Say L, Chou D. Equity and intrapartum care by skilled birth attendant globally : protocol for a systematic review. 2018;1–5.

150. Martinez O, Wu E, Sandfort T, Dodge B, Carballo-Dieguez A, Pinto R, et al. Evaluating the Impact of Immigration Policies on Health Status Among Undocumented Immigrants: A Systematic Review. J Immigr Minor Heal [Internet]. 2015 Jun 28;17(3):947–70. Available from: http://link.springer.com/10.1007/s10903-013-9968-4

151. Sawin EM, Sobel LL, Annan SL, Schminkey DL. From Systematic Review to Call for Action : In Search of Evidence-Based Interventions to Decrease Intimate Partner Violence in Rural Hispanic American Women. 2017;15(2):79–87.

152. Mcfadden A, Siebelt L, Gavine A, Atkin K, Bell K, Innes N, et al. Gypsy , Roma and Traveller access to and engagement with health services : a systematic review. 2018;28(1):74–81.

153. Alcazar-Bejerano IL. Health Behaviors, Disparities and Deterring Factors for Breast Cancer Screening of Immigrant Women - A Challenge to Health Care Professionals. J Lifestyle Med [Internet]. 2014 Mar 31;4(1):55–63. Available from: http://www.jlifestylemed.org/journal/DOIx.php?id=10.15280/jlm.2014.4.1.55

154. McCollum R, Gomez W, Theobald S, Taegtmeyer M. How equitable are community health worker programmes and which programme features influence equity of community health worker services? A systematic review. BMC Public Health. 2016;16(1):1–16.

155. Agency for Healthcare Research and Quality. Improving cultural competence to reduce health disparities for priority populations. Eff Heal Care Progr [Internet]. 2014;(170):1–19. Available from: www.effectivehealthcare.ahrq.gov

156. Diaz E, Ortiz-Barreda G, Ben-Shlomo Y, Holdsworth M, Salami B, Rammohan A, et al. Interventions to improve immigrant health. A scoping review. Eur J Public Health. 2017;27(3):433–9.

157. Tiwari T, Jamieson L, Broughton J, Lawrence HP, Batliner TS, Arantes R, et al. Reducing Indigenous Oral Health Inequalities: A Review from 5 Nations. J Dent Res. 2018;97(8):869–77.

158. Pons-Vigués M, Diez È, Morrison J, Salas-Nicás S, Hoffmann R, Burstrom B, et al. Social and health policies or interventions to tackle health inequalities in European cities: A scoping review. BMC Public Health. 2014;14(1).

159. de Silva AM, Meyenn LK, Calache H, Hegde S, Akudo Nwagbara B, Gussy MG, et al. Community-based population-level interventions for promoting child oral health. Cochrane Database Syst Rev. 2016;2016(9).

160. Benmarhnia T, Rey L, Cartier Y, Clary CM, Deguen S, Brousselle A. Addressing equity in interventions to reduce air pollution in urban areas: a systematic review. Int J Public Health. 2014;59(6):933–44.
