# Supplementary material for: An overview of reviews on strategies to reduce health inequalities
Source: Int J Equity Health. 2020 Oct 28;19:192. doi: 10.1186/s12939-020-01299-w (PMC7594271; doi:10.1186/s12939-020-01299-w)
Supplement: Supplementary file 3 — Additional file 3. Characteristics of studies included in the evidence synthesis. [file 12939_2020_1299_MOESM3_ESM.docx]

Additional file 3. Characteristics of studies included in the evidence synthesis

| **Nº** | **Author** | **Year*** | **Total of studies included** | **Countries**** | **Population** | **Approach Health thematic** | **Sector** | **Type of interventions ***** | **Quality of the review ****** | **Main conclusion** |
| --- | --- | --- | --- | --- | --- | --- | --- | --- | --- | --- |
| **1** | Araujo M, et.al. (1) | 2016 | 22 | United States, Canada | Indigenous population | General health | Social Health Politics | Delivery arrangements, Implementation Strategies | Moderate | Interventions do exist that have potential for producing positive effects on access to health services by indigenous populations in the Americas, but available studies are limited to Canada and the United States. |
| **2** | Man LC, et.al. (2) | 2018 | 7 | United States | African American, Hispanic and Asian population | Cancer | Social Health | Delivery arrangements | Critially Low | The Initiative was is a health system-based learning community developed to address colorectal cancer screening disparities among minority patients in primary care. |
| **3** | Coughlin SS, et.al. (3) | 2016 | 15 | United States | African American population | Healthy lifestyles | Social Health | Delivery arrangements | Critially Low | Additional community-based participatory research studies and faith-based interventions are needed to identify effective ways to promote physical activity in African American communities and to address health disparities. Of particular interest are community-engaged studies that have an adequate sample size and a rigorous design, and which overcome limitations of previous studies, especially in states such as Georgia that have substantial health disparities |
| **4** | Rodrigues AL, et.al. (4) | 2016 | 25 | China, Norway, Canada, Vietnam, Netherlands, Malaysia, India, United States, | Rural population | Cardiovascular disease | Social Health | Implementation Strategies, Delivery arrangements | Critially Low | The setting for applying cardio-metabolic prevention programmes is important given its likelihood to influence programme efficacy. Further investigation is needed to elucidate the individual determinants of cardio-metabolic risk in non-urban populations and in contrast to urban populations |
| **5** | Hamilton K, et.al. (5) | 2018 | 20 | United States, Australia, Chile, Israel, France, Brazil, Peru, Switzerland | Vulnerable population | Tuberculosis | Social Health | Implementation Strategies, Delivery arrangements, Financial arrangements | Moderate | The strongest evidence for improving screening uptake and completion was for incentives, with mixed evidence for peer support. Descriptive evidence showed that professional support and mandatory screening could improve uptake. |
| **6** | Lee-Tauler SY, et.al. (6) | 2018 | 29 | United States | African American, Hispanic and Asian population | Mental health | Health | Delivery arrangements | Moderate | It found evidence that integrated care models hold promise in reducing these disparities. Importantly, effective interventions to reduce disparities in care must be widely disseminated and implemented to be of value. |
| **7** | Starbird LE, et.al. (7) | 2019 | 10 | Canada, Uganda, United States | Adults | Chronic diseases | Social Health Politics  Transport | Governance arrangements, Financial arrangements | Moderate | This systematic review found evidence to support the provision of bus passes, taxi vouchers, and reimbursement for transportation to improve healthcare utilization and chronic disease health outcomes among diverse socioeconomically disadvantaged populations, particularly older adults |
| **8** | Melvin CL, et.al. (8) | 2017 | 29 | United States | Minorities (racial or ethnic group) | Healthy lifestyles | Social Health | Delivery arrangements, Implementation Strategies, | Moderate | It is important to consider why interventions targeting weight loss through health behavior change had significant effects on weight outcomes, but did not substantially change diet and physical activity in all cases. Fruit and vegetable intake were among the metrics used to evaluate the effects of weight loss interventions on dietary behaviors. Many patients in these studies had limited socioeconomic resources that likely reduced their capacity to make dietary changes. |
| **9** | Pratt CA, et.al. (9) | 2017 | 49 | United States | African American, Hispanic and Indigenous population | Obesity | Health | Delivery arrangements | Critially Low | The reviews call for cardiovascular-related obesity disparities research that is long term and includes population research, and multilevel, policy, and environmental, or “whole of community,” interventions |
| **10** | Tovar A, et.al. (10) | 2014 | 20 | United States | Hispanic population | Obesity | Health | Governance arrangements, Implementation Strategies, | Moderate | The number of obesity prevention interventions among immigrant populations identified in this systematic review does not reflect the rapid demographic and cultural transformation that the United States has experienced over the last two decades. |
| **11** | Hillier-Brown FC, et.al. (11) | 2014 | 28 | United States, Brazil, Chile, Peru, Netherlands, France, Australia, Israel. | Infant/adolescent population | Obesity | Health | Delivery arrangements, Implementation Strategies | Moderate | The review has found only limited evidence although some individual and community based interventions may be effective in reducing socio-economic inequalities in obesity-related outcomes amongst children but further research is required, particularly of more complex, societal level interventions and amongst adolescents. |
| **12** | Hillier-Brown FC, et.al. (12) | 2014 | 20 | Australia, Turkey, United Kingdom, United States | Adults | Obesity | Economic Health | Delivery arrangements, Implementation Strategies | Low | The best available international evidence suggests that some individual and community-based interventions may be effective in reducing socio-economic inequalities in obesity among adults in the short term. Further research is required particularly of more complex, multifaceted and societal-level interventions |
| **13** | Wang L, et.al. (13) | 2016 | 15 | Hungary, Ireland, United Kingdom, United States, Spain, Sweden, Italy, Germany | General population | Environmental pollution | Environment Health | Implementation Strategies | Critially Low | This review not only highlighted the effectiveness and the need for environmental strategies to improve air quality and health, but also explored the connections between socioeconomic status, vulnerability and air pollution exposure. The health co-benefits obtained from the four groups of air pollution control strategies indicated that there was a strong case for promoting Health in All Policies (HiAP) |
| **14** | McGill R, et.al. (14) | 2015 | 36 | United Kingdom | General population | Healthy lifestyles | Health Politics Economic | Delivery arrangements | Low | Interventions categorised by a “6 Ps” framework show differential effects on healthy eating outcomes by socioeconomic position (SEP). “Upstream” interventions categorised as “Price” appeared to decrease inequalities, and “downstream” “Person” interventions, especially dietary counselling seemed to increase inequalities. However the vast majority of studies identified did not explore differential effects by SEP. |
| **15** | Yadee J, et.al. (15) | 2019 | 13 | United States, Spain | Immigrants / displaced | General health | Health | Delivery arrangements | Moderate | Current evidence reveals that there is a paucity of studies assessing equity attributes of health interventions developed for migrant populations. This indicates that equity has not been receiving attention in these studies of migrant populations. More attention to equity-focused outcome assessment is needed to help policy-makers to consider all relevant outcomes for sound decision making concerning migrants. |
| **16** | Martinez O, et.al. (16) | 2017 | 13 | United States | Adults | Human immunodeficiency virus | Judicial Health Social | Delivery arrangements | Critially Low | This study was the first comprehensive systematic literature review on the impact of Medical-legal partnerships (MLPs) in addressing health disparities and improving health outcomes. These results speak to the potential for MLPs to address health and legal needs, while highlighting the need for more rigorous investigations (including longitudinal assessments) to better capture the efficacy and impact of MLPs. |
| **17** | Harris J, et.al. (17) | 2015 | 13 | United States | Minorities (racial or ethnic group) | Health literacy | Social Politics Education | Delivery arrangements, Implementation Strategies | Low | Peer-support programmes have the potential to improve health literacy (HL) and reduce health inequalities but potential is dependent upon the surrounding equity context. More explicit empirical research is needed, which establishes clearer links between peer-supported HL and health inequalities. |
| **18** | LM A, et.al. (18) | 2015 | 59 | United States, Australia, Canada, England, Netherlands | Minorities (racial or ethnic group) | General health | Social Health | Delivery arrangements, Implementation Strategies | Moderate | Coalition-led interventions are characterized by connection of multi-sectoral networks of health and human service providers with ethnic and racialminority communities. These interventions benefit a diverse range of individual health outcomes and behaviors, as well as health and social care delivery systems. Evidence in this review shows that interventions led by community coalitions may connect health and human service providers with ethnic and racial minority communities in ways that benefit individual health outcomes and behaviors, as well as care delivery systems. However, because information on characteristics of the coalitions themselves is insufficient, evidence does not provide an explanation for the underlying mechanisms of beneficial effects. |
| **19** | Verhagen I, et.al. (19) | 2014 | 7 | United States, Mexico | Minorities (racial or ethnic group) in older alduts | General health | Health | Delivery arrangements, Implementation Strategies | Low | Community health workers (CHWs) serve as a means of improving health care use and health behaviour and, to a lesser extent, health outcomes among ethnic minority older adults. Further research is required to draw more solid conclusions on the effectiveness of CHW interventions in this target group |
| **20** | Banna J, et.al. (20) | 2018 | 49 | United States | Indigenous population | Chronic diseases | Health | Delivery arrangements, Implementation Strategies | Low | The extent of use of strategies to promote community engagement in programs reporting significant outcomes is notable. In planning interventions in Indigenous groups, researchers should consider ways to involve the community in intervention design, execution and evaluation. There is a particular need for studies focused on Indigenous youth in diverse regions of the United States to further address diet-related chronic conditions. |
| **21** | Walton-Moss B, et.al. (21) | 2014 | 32 | Australia, China, Guatemala, Netherlands, Norway, Pakistan, South Africa, Sweden, United States | Vulnerable population | Cardiovascular disease | Health | Delivery arrangements, Implementation Strategies | Critially Low | Blood pressure interventions were the most promising while behavior change interventions (such as those targeting increased physical activity or decreased smoking) were the most challenging. Education interventions were the most common type of intervention although almost half of interventions were multi-component. Almost all of the interventions were at the individual level. Finally, differential effects of interventions were observed with some results that were moderated by age, sex, education or residence. |
| **22** | Coughlin SS, et.al. (22) | 2017 | 18 | United States | African American population | Healthy lifestyles | Health | Delivery arrangements, Implementation Strategies | Critially Low | To address health disparities, additional community-based participatory research (CBPR) studies are needed to promote healthy diet, nutrition, and weight management in African American communities. Of particular interest are multilevel CBPR studies that include interventions aimed at multiple levels of the socioecological model. |
| **23** | Vallury KD, et.al. (23) | 2015 | 10 | Australia, New Zealand, United Kingdom | Rural population | Mental health | Health Politics Telecommunication | Delivery arrangements | Moderate | Computerized cognitive behavior therapy (CCBT), can be effective for addressing depression and anxiety and is acceptable among rural participants. Further work is required to confirm these results across a wider range of countries, and to determine the most feasible model of CCBT delivery, in partnership with people who live and work in rural and remote communities. |
| **24** | Gardner F, et.al. (24) | 2017 | 14 | United Kingdom | Minorities (racial or ethnic group) | Mental health | Economic Health | Implementation Strategies | High | There is no evidence that the benefits of the Incredible Years® (IY) parenting intervention are reduced in disadvantaged or minority families; benefits are greater in the most distressed families, including parents who are depressed. Thus, the intervention is unlikely to widen socioeconomic inequalities in disruptive behaviour and may have effects in narrowing inequalities due to parent depression. It was as likely to be effective for older as for younger children |
| **25** | Dauvrin M, et.al. (25) | 2014 | 61 | New Zealand, United States, United Kingdom, Mexico, Canada, Germany, | General population | Diabetes | Health | Delivery arrangements, Implementation Strategies | Critially Low | Overall, culturally competent (CC) interventions addressing Type 2 diabetes mellitus are not congruent with the reduction of ethnic health inequalities. The future of CC interventions may involve going one step further and going back to basic tenets of cultural competence: the integration of difference, whatever its source, into the delivery of fair health care for patients. |
| **26** | Skeie MS, et.al. (26) | 2018 | 37 | Australia, Austria, Brazil, France, Germany, England, Ireland, Netherlands, Iran, India, Portugal, Sweden, Spain, Scotland, United States, Turkey, Tanzania | Vulnerable population | Oral health | Health | Implementation Strategies, Delivery arrangements | Moderate | Studies of water fluoridation and fluoride toothpaste, there are other preventive intervention studies providing scientific evidence for caries reduction among children and adolescents with immigrant or low socioeconomic backgrounds. |
| **27** | Durand MA, et.al. (27) | 2014 | 19 | Australia, Nicaragua, United States | Vulnerable population | General health | Social Health | Delivery arrangements, Implementation Strategies | High | Results indicate that shared decision-making (SDM) interventions significantly improve outcomes for disadvantaged patients. According to the narrative synthesis, SDM interventions may be more beneficial to disadvantaged groups than higher literacy/socioeconomic status patients. However, given the small sample sizes and variety in the intervention types, study design and quality, those findings should be interpreted with caution. |
| **28** | Pottie K, et.al. (28) | 2014 | 13 | Tanzania, Zimbabwe, South Africa, United States, Nigeria, Kenya, Australia, Uganda | Vulnerable population | Human immunodeficiency virus (HIV) | Health | Delivery arrangements | High | Rapid voluntary counselling and testing (VCT) in health facilities and communities was associated with a large increase in HIV-testing uptake and receipt of results. The routine use of rapid VCT may also help avoid human rights violations among marginalised populations where testing may occur without informed consent and where existing stigma may create barriers to testing. |
| **29** | Batsis JA, et.al. (29) | 2019 | 17 | United States | Older adults | Telemedicine | Health Telecommunication Social | Delivery arrangements | Moderate | Telemedicine is feasible and acceptable in delivering care to older adults. Research should focus on well-designed randomized trials to overcome the high degree of bias observed in our synthesis. Clinicians should consider using telemedicine in routine practice to overcome barriers of distance and access to care. |
| **30** | Pinzón Flórez CE, et.al. (30) | 2015 | 17 | Bangladesh, India, Pakistan, Uganda, South Africa, Burkina Faso, Nigeria, Ghana, Mexico, Argentina, Philippines | Maternal infant | Maternal infant health | Health | Implementation Strategies, Delivery arrangements | Moderate | Inclusion of community workers is effective in reducing maternal and infant mortality in low- and middle-income. Moreover it is improving access and coverage of health services to vulnerable populations |
| **31** | Joo JY, et.al. (31) | 2014 | 9 | United States | Immigrants / displaced | Diabetes | Social Health Education | Delivery arrangements, Implementation Strategies | Moderate | There is strong evidence of the effectiveness of tailoring diabetes interventions to Asian immigrant populations’ cultures. Further studies, including longitudinal studies and studies with rigorous research designs that subclassify Asian immigrants, are needed to encourage the implementation of culturally tailored diabetes intervention for this ethnic minority. |
| **32** | LaCroix JM, et.al. (32) | 2014 | 54 | Armenia, Australia, Botswana, Burkina Faso, Cameron, China, Colombia, Gabón, Ghana, Guinea, India, Jamaica, Kenya, Lesotho, Madagascar, Malawi, Mozambique, Nepal, Netherlands, Nicaragua, Nigeria, Peru, Rwanda, Saint Lucia, Sierra Leone, South Africa, Switzerland, Tanzania, Thailand, Uganda, United Kingdom, United States, Zambia, Zimbabwe | General population | Human immunodeficiency virus | Health Politics Social | Delivery arrangements, Implementation Strategies | Low | the present results provide strong testimony to the power that mass media campaigns can have for people living in nations most at need for HIV prevention and other health promotion interventions. Results also suggest that such campaigns generally lack effectiveness in relatively developed countries, where the need for health promotion is generally lessened except in particular locales |
| **33** | Escribà-Agüir V, et.al. (33) | 2016 | 17 | United States, Israel | African American, Hispanic and Asian population | Cancer | Health | Delivery arrangements, Financial arrangements, Implementation Strategies | Moderate | Culturally adapted patient-targeted healthcare interventions can help to reduce racial or ethnic inequalities in access to cancer screening programmes. Further research is needed to develop interventions to promote adherence to cancer screening programmes with repeat testing and vigorous economic evaluation methodologies |
| **34** | Mbuzi V, et.al. (34) | 2018 | 8 | Australia | Indigenous population | Cardiovascular disease | Health | Delivery arrangements, Implementation Strategies | Moderate | The limited evidence available demonstrated that interventions targeted at Indigenous cardiovascular health and related risk factors can be effective. The results indicate that there are opportunities to improve cardiovascular health of Indigenous people at all stages of the disease continuum. There is a need for further research into evidence-based interventions that are sensitive to Indigenous culture and needs. |
| **35** | Reilly R, et.al. (35) | 2016 | 10 | Australia, New Zealand, Canada | Indigenous population | Chronic kidney | Health | Implementation Strategies, Delivery arrangements | Low | Given the human cost of dialysis and the growing population of people living with chronic kidney disease , there is an urgent need to draw lessons from the available evidence from this and other sources, including studies in the broader population, to better serve this population with programs that address the barriers to receiving high-quality care and improve quality of life. |
| **36** | Blanchard AK, et.al. (36) | 2019 | 22 | Brazil, Kenya, India, Pakistan, Nepal, Bangladesh, Vietnam, Malawi | Maternal infant | Maternal infant health | Health | Implementation Strategies, Financial arrangements | Moderate | While current evidence remains limited, particularly for mortality, existing studies suggest that Community health worker (CHW) interventions involving home visits, cash transfers, participatory women’s groups or multiple components can improve equity in maternal and newborn health. Future mixed-methods research should explore intervention strategies and contextual processes shaping such effects on equity to optimise these efforts. |
| **37** | Kim K, et.al. (37) | 2016 | 67 | Canada, United States, India, Pakistan, Taiwán | Vulnerable population | Chronic diseases | Health | Financial arrangements | Moderate | Interventions by Community-based health workers (CBHWs) appear to be effective when compared with alternatives and also cost-effective for certain health conditions, particularly when partnering with low-income, underserved, and racial and ethnic minority communities. Future research is warranted to fully incorporate CBHWs into the health care system to promote noncommunicable health outcomes among vulnerable populations |
| **38** | Hahn RA, et.al. (38) | 2014 | 40 | United States | Minorities (racial or ethnic group) in children | Quality of life | Education Health | Delivery arrangements, Implementation Strategies | Critially Low | There is strong evidence that full-day kindergarten (FDK) improves academic achievement, a predictor of longer-term health benefits. To sustain early benefits, intensive elementary school education is needed. If targeted to low-income and minority communities, FDK can advance health equity. |
| **39** | Speyer R, et.al. (39) | 2018 | 44 | Australia, Canada, Guam, Italy, Sweden, United Kingdom, United States | Rural population | General health | Health | Delivery arrangements | High | Telehealth services may be as effective as face-to-face interventions, which is encouraging given the potential benefits of telehealth in rural and remote areas with regards to healthcare access and time and cost savings. |
| **40** | Antoniades J, et.al. (40) | 2014 | 15 | United States | Immigrants / displaced | Mental health | Health | Delivery arrangements | High | Depression may be effectively treated by means of psychotherapies, especially when treatments are culturally adapted. However the reviewed studies were limited due to methodological weaknesses and were predominantly undertaken in the United States with Latino patients |
| **41** | Parker S, et.al. (41) | 2018 | 18 | United States, Australia, Canada | Vulnerable population | Chronic diseases | Health Social Politics Telecommunication | Delivery arrangements | Low | Research incorporating these tools with vulnerable groups is not comprehensive. Apart from intrinsic motivation, health literacy may also influence the reaction of vulnerable groups to technology. Social persuasion was the main way interventions sought to achieve better self-management. Efforts to engage patients by healthcare providers were lower than expected. Use of social networks or other eHealth mechanisms to link patients and provide opportunities for vicarious experience could be further explored in relation to vulnerable groups. |
| **42** | Abbott LS, et.al. (42) | 2017 | 39 | United States | Minorities (racial or ethnic group) | General health | Health | Implementation Strategies, Delivery arrangements | Moderate | Community and home visitation interventions by nurses can provide an effective means for mitigating social determinants of health by empowering people at risk for health disparities to avoid injury, maintain health, and prevent and manage existing disease. |
| **43** | Brown T, et.al. (43) | 2014 | 29 | Belgium, Denmark, France, Israel, Netherlands, Poland, Spain, Turkey, United Kingdom, United Kingdom | General population | Healthy lifestyles | Health | Implementation Strategies | Moderate | Untargeted smoking cessation interventions in Europe may have contributed to reducing adult smoking but are, on balance, likely to have increased inequalities in smoking. However, United Kingdom National Health Service stop-smoking services appear to reduce inequalities in smoking through increased relative reach through targeting services to low- status smokers. More research is needed to strengthen the evidence-base for reducing smoking inequalities. |
| **44** | Brown T, et.al. (44) | 2014 | 38 | Canada, Finlandia, France, Germany, Ireland, Israel, Netherlands, New Zealand, Portugal, Spain, Sweden, United Kingdom, United States | Infant/adolescent population | Healthy lifestyles | Health | Implementation Strategies | Low | Very few studies have assessed the equity impact of tobacco control interventions/policies on young people. Price/tax increases had the most consistent positive equity impact. There is a need to strengthen the evidence base for the equity impact of youth tobacco control interventions. |
| **45** | Brown T, et.al. (45) | 2014 | 117 | Australia, Belgium, Canada, Croatia, France, Germany, Ireland, Italy, Netherlands, New Zealand, n, Sweden, United Kingdom, United States | General population | Healthy lifestyles | Health Economic Social Politics | Implementation Strategies | Moderate | Few studies have assessed the equity impact of tobacco control policy/interventions. Price/tax increases had the most consistent positive equity impact. More research is needed to strengthen the evidence-base for reducing smoking inequalities and to develop effective equity-orientated tobacco control strategies |
| **46** | Attwood S, et.al. (46) | 2016 | 173 | United Kingdom, United States, Canada, Spain, Australia, Netherlands | General population | Healthy lifestyles | Social Health | Delivery arrangements | Low | The majority of randomised controlled trials (RCTs) of physical activity interventions in primary care record sufficient information on PROGRESS-Plus factors to allow differential effects to be studied. However, very few actually report details of relevant analyses to determine which population subgroups may stand to benefit or be further disadvantaged by intervention efforts |
| **47** | Cyril S, et.al. (47) | 2015 | 24 | Bangladesh, Canada, China, India, Iran, Kenya, United Kingdom, United States | Vulnerable population | General health | Health | Governance arrangements, Delivery arrangements | Low | The findings suggest that community engagement (CE) models can lead to improved health and health behaviours among disadvantaged populations if designed properly and implemented through effective community consultation and participation. |
| **48** | Ashman AM, et.al. (48) | 2017 | 20 | Australia, Canada, United States | Pregnant Indigenous population | Nutrition | Health | Implementation Strategies | Moderate | Heterogeneity of included studies made it challenging to make firm recommendations regarding program success. Authors of included studies recommended community consultation be included when designing studies and working with communities at all stages of the research process. Individualized counseling/education can contribute to successful programoutcomes, as can the use of Indigenousworkers to deliver program content. |
| **49** | Kristjansson E, et.al. (49) | 2015 | 21 | India, Bangladesh, Jamaica, Indonesia, Colombia, Malawi, Níger, Nigeria, Kenya, Peru, South Africa, Vietnam, Thailand, Brazil, Ecuador, Haiti, Mexico, Bolivia, Caledonia, Congo, Senegal, Australia, Canada, United States | Infant/adolescent population | Nutrition | Health | Delivery arrangements, Implementation Strategies | High | Feeding programmes for young children in low- and middle-income countries can work, but good implementation is key. |
| **50** | Valdez LA, et.al. (50) | 2018 | 12 | United States | Hispanic population | Healthy lifestyles | Social Health | Implementation Strategies, Delivery arrangements | Moderate | While studies are limited and findings are mixed, culturally tailored work shows promise. The growth rate of the Latino population and the current epidemic nature of substance abuse in the United States generate urgency to identify methods to diminish the disparate burden of alcohol and substance abuse in Latinos. |
| **51** | Fernández-Gutiérrez M, et.al. (51) | 2017 | 9 | United States | Immigrants / displaced | General health | Education Health Social | Implementation Strategies | Moderate | The interventions studied were reported as being effective in improving health literacy in immigrants, particularly the functional aspects. Regarding the role played by nursing, this review observed little involvement |
| **52** | Kim SH, et.al. (52) | 2016 | 13 | United States | General population | Diabetes | Health | Delivery arrangements, Implementation Strategies | Low | Healthcare providers should consider active implementation of strategies for accommodating people with low health literacy in diabetes self-management interventions. The routine use of spoken communication strategies would be necessary to achieve the best health outcomes in diabetes self-management interventions |
| **53** | Mojica CM, et.al. (53) | 2018 | 7 | United States | Hispanic population | Cancer | Health | Delivery arrangements, Implementation Strategies | Moderate | Although intervention studies focused on colorectal cancer screening among men of racial/ethnic minorities are scarce, our findings highlight promising strategies that were effective at increasing colorectal cancer screening among Latino men. Additional research in the area of Latino men’s health is needed, especially to further develop and test theoretically grounded interventions that promote colorectal cancer screening with larger samples of men and across diverse geographic areas in the United States. |
| **54** | Mayén AL, et.al. (54) | 2016 | 7 | Brazil, Chile, Colombia, Iran, Panama, Trinidad and Tobago, Tunicia | Adults | Healthy lifestyles | Health | Implementation Strategies | Critially Low | In low- and middle-income countries (LMICs), agentic interventions promoting healthy eating reduced social inequalities in diet when specifically targeting disadvantaged populations. Further research should assess the impact on social inequalities in diet of a combination of agentic and structural approaches in interventions promoting healthy eating |
| **55** | Robinson JL, et.al. (55) | 2017 | 21 | United States, South Africa, Zambia, Zimbabwe, Nigeria | Adults (Women) | Human immunodeficiency virus | Health | Implementation Strategies | Moderate | While more research is needed, the limited existing evidence suggests that these interventions may help support the sexual and reproductive health (SRH) and rights of women living with HIV. This review particularly highlights the importance of these interventions for preventing sexually transmitted infections (STIs), which present a significant health burden for women living with HIV that is rarely addressed holistically. Empowerment- based interventions should be considered as part of a comprehensive package of STI and other SRH services for women living with HIV. |
| **56** | Barley EA, et.al. (56) | 2016 | 0 | No studies included | General population | Mental health | Health | Delivery arrangements, Financial arrangements | Moderate | A comprehensive search showed that currently there is no RCT evidence for any method of encouraging cancer screening uptake in people with severe mental illness (SMI). No specific approach can therefore be recommended. High-quality, large-scale RCTs are needed urgently to help address the disparity between people with SMI and others in cancer screening uptake. |
| **57** | Ramke J, et.al. (57) | 2017 | 2 | China | General population | General Health | Health | Delivery arrangements | Moderate | Current evidence on the effect on equity of interventions to improve access to cataract services in LMICs is limited. They identified only two studies, both conducted in rural China. Assessment of equity effects will be improved if future studies disaggregate outcomes by relevant social subgroups. To assist with assessing generalisability of findings to other settings, robust data on contextual factors are also needed. |
| **58** | Ontario Health Quality (58) | 2015 | 5 | United States | Vulnerable population | General health | Health | Implementation Strategies, Delivery arrangements | Moderate | Moderate-quality evidence indicates that orientation to clinic services (either alone or combined with outreach) improves access to a primary care provider in adults who are homeless, without serious mental illness, and living in urban centres. |
| **59** | Clifford A, et.al. (59) | 2015 | 16 | United States, Australia | Indigenous population | General health | Health | Implementation Strategies | Moderate | There is a lack of evidence from rigorous evaluations on the effectiveness of interventions for improving cultural competency in health care for Indigenous peoples. Future evaluations should employ more rigorous study designs and extend their measurement of outcomes beyond those relating to health professionals, to those relating to the health of Indigenous peoples |
| **60** | Mercer, et.al. (60) | 2019 | 13 | Nepal, Tanzania | General population | Oftalmology | Health | Implementation Strategies | Moderate | Limited evidence exists to inform health service planners regarding interventions to reduce gender inequity in visual impairment and blindness. Training community volunteers to identify and counsel affected individuals, and empower them to circumvent or challenge socioeconomic barriers to accessing care holds promise. |
| **61** | Rees I, et.al. (61) | 2018 | 13 | France, United States, Mexico, Thailand, Italy, Sweden | Adults (Women) | Cancer | Health | Implementation Strategies, Delivery arrangements | Moderate | HPV self-testing can improve uptake of cervical cancer screening among lower socioeconomic groups. This review also provides additional evidence on the effectiveness of Lay health advisors (LHAs)-led interventions; and suggests that intensive, culturally appropriate education, based on health-behavior models, can be effective, particularly if help is given to the women to help overcome structural barriers. This review has also shown mixed strategies utilizing LHAs with additional outreach and media support can be effective at increasing cervical cancer screening rates. |
| **62** | Bhui KS, et.al. (62) | 2015 | 21 | United Kingdom, United States | Minorities (racial or ethnic group) | Mental health | Social Health | Delivery arrangements, Implementation Strategies | Low | Culturally adapted psychotherapies, and ethnographic and motivational assessment leading to psychotherapies were effective and favoured by patients and carers. |
| **63** | Hu D, et.al. (63) | 2014 | 36 | United States | Hispanic and African American population | General health | Health | Delivery arrangements, Implementation Strategies | Critially Low | The studies varied significantly in their methods and design as well as the populations studied. While some types of interventions (eg, motivational interviewing, reminder devices, one-time educational session) have been effective, no one intervention was proven to be universally successful. A consensus on a “gold standard” for measuring medication adherence would further contribute to facilitating the comparison of different interventions. For patients taking multiple medications, there exists a need for such research in the development of interventions to improve adherence. |
| **64** | Crocker-Buque T, et.al. (64) | 2017 | 41 | United Kingdom, United States, Scotland, Canada y Australia | Infant/adolescent population | Vaccunation | Health | Delivery arrangements | Critially Low | Locally designed, multicomponent interventions should be used in urban, ethnically diverse, deprived populations. Some evidence is emerging for text-message reminders, particularly in adolescents. |
| **65** | Raison H, et.al. (65) | 2019 | 6 | United States | Maternal infant | Maternal infant – Oral health | Health | Implementation Strategies, Delivery arrangements | Moderate | Evidence in this area is limited and results are mixed. More work is needed to investigate the effectiveness of interventions to reduce SES inequalities, especially in different healthcare systems and involving a wider participant range |
| **66** | Moore KL, et.al. (66) | 2018 | 10 | United States | Infant/adolescent population | General health | Health | Delivery arrangements, Implementation Strategies | Moderate | Cultural adaptations to improve engagement in mental health services will need to be highly adaptive to young adults individual identities as well as to their developmental needs to be relevant and effective. |
| **67** | Anderson-Lewis C, et.al. (67) | 2018 | 16 | United States | Minorities (racial or ethnic group) | General health | Health | Delivery arrangements | Critially Low | mHealth is a promising area of development for public health and health education. While successful research has been done using text messaging (short message service, SMS) and other mHealth strategies, there is a need for more research using mobile phones and tablet applications. This literature review demonstrates mHealth technology has the ability to increase prevention and health education in health disparate communities and concludes that more specified research is needed. |
| **68** | Santos Salas A, et.al. (68) | 2016 | 3 | United States | Vulnerable population | Cancer | Social Health | Governance arrangements, Implementation Strategies | Low | Findings from this review point to the need to develop effective supportive care interventions designed to serve cancer populations affected by social disparities. Comprehensive, long-term, gender-sensitive, and culturally appropriate interventions are needed to enhance pain and symptom relief in vulnerable populations. Some minorities bear a larger cancer burden than their counterparts and are therefore overrepresented in the cancer population. |
| **69** | Goudet SM, et.al. (69) | 2019 | 15 | Bangladesh, India, Peru | Infant/adolescent population | Nutrition | Health | Implementation Strategies | High | All the nutritional interventions reviewed had the potential to decrease stunting, based on evidence from outside of slum contexts; however, there was no evidence of an effect of the interventions included in this review. Challenges linked to urban slum programming (high mobility, lack of social services, and high loss of follow-up) should be taken into account when nutrition-specific interventions are proposed to address low birth weight and stunting in such environments. More evidence is needed of the effects of multi-sectorial interventions, combining nutrition-specific and sensitive methods and programmes, as well as the effects of ’up-stream’ practices and policies of governmental, non-governmental organisations, and the business sector on nutrition related outcomes such as stunting. |
| **70** | Tsai C, et.al. (70) | 2017 | 19 | United States, Brazil, Canada, Australia, Taiwan | Indigenous population | Oral health | Health | Delivery arrangements | Moderate | Successful programmes considered cultural appropriateness in their design. Collaborative approaches were seen as an advantage, not only with Indigenous communities, but also with local health providers and organizations, with particular emphasis on incorporation into existing services. Challenges included low community participation, unstable funding and staffing issues. |
| **71** | Khanassov V, et.al. (71) | 2016 | 36 | Canada, Australia | Vulnerable population | General health | Health | Delivery arrangements | Critially Low | While there appears to be a limited number of published research studies about organizational interventions aimed at improving access to primary care for vulnerable populations, this scoping review showed that there are enough studies for a future systematic review to test the following hypothesis: formal integration of services (increased approachability, availability and affordability of primary care services) could be associated with a reduction of hospitalization, emergency room admission and unmet health care needs |
| **72** | McCurley JL, et.al. (72) | 2017 | 15 | United States | Young Hispanic population | Diabetes | Health | Delivery arrangements | Moderate | Few interventions demonstrated success in reducing body mass index and glucose regulation and follow-up times were brief. More studies are needed that recruit larger samples sizes, extend follow-up times, explore innovative delivery modalities, and examine effectiveness across sex and age. |
| **73** | Mitchell SA, et.al. (73) | 2016 | 23 | The review does not informe | Rural population (Women) | Healthy lifestyles | Economic Health Social | Delivery arrangements | Critially Low | Few studies have focused on the specific needs and difficulties of smoking cessation among rural low-income women and interventions have not targeted the complex social network of this population. Incongruence in study findings supports the need for smoking assessment and cessation interventions that incorporates the unique social and cultural meanings of smoking in rural low-income women. |
| **74** | Moore GF, et.al. (74) | 2015 | 20 | United Kingdom, Norway, Netherlands, New Zealand, Austria, Belgium, Germany, Grecia, Italy, Spain, Sweden, India, Denmark | Infant/adolescent population | General health | Health | Implementation Strategies | Moderate | Universal school-based interventions may narrow, widen or have no effect on inequality. There is a significant need for more routine testing of the effects of such interventions on inequality to enable firmer conclusions regarding types of interventions which affect inequality. |
| **75** | Heller C, et.al. (75) | 2014 | 20 | The review does not inform | Minorities (racial or ethnic group) | General health | Health | Implementation Strategies, Delivery arrangements | Critially Low | Multiple and flexible strategies targeting providers and participants at provider sites and within communities might be needed to enroll underrepresented populations into clinical trials |
| **76** | Jia L (76) | 2014 | 2 | United States | Vulnerable population | General health | Health | Governance arrangements | Moderate | Community-based case managers who provide health insurance information, application support, and negotiate with the insurer probably increase enrolment of children in health insurance schemes. However, the transferability of this intervention to other populations or other settings is uncertain. Handing out insurance application materials in hospital emergency departments may help increase the enrolment of children in health insurance schemes. |
| **77** | Domingo JLB, et.al. (77) | 2018 | 9 | United States | Minorities (racial or ethnic group) | Cardiovascular disease (CVD) | Education Health | Delivery arrangements | Moderate | Given the high prevalence of CVD in FA populations, tailored interventions rooted in Filipino cultural values are vital to address this known health disparity. Health education curricula should acknowledge the cultural significance of food, encourage family participation in sessions, and foster a supportive environment to build relationships among participants. |
| **78** | Sharma BB, et.al. (78) | 2018 | 11 | Nepal, Bangladesh, India, Pakistan | Rural population (Women) | General health | Education Health | Implementation Strategies, Delivery arrangements | High | Women’s education interventions may improve the number of women seeking birth at a health care facility, but the evidence is of low quality. No impact on maternal mortality was observed. Future research should explore the effectiveness of including male mobilisers |
| **79** | Luque JS, et.al. (79) | 2017 | 5 | Bangladesh, India, Nepal, Pakistan | Women Hispanic population | General health | Health | Implementation Strategies | Critially Low | This review has demonstrated that rigorously designed community-based studies employing promotoras who use motivational interviewing techniques can be successfully implemented in Hispanic communities and produce positive mammography screening outcomes. Future studies need to examine the financial sustainability of these types of cancer education intervention programs, taking into account different health care systems. The eventual adoption of promotora-led educational interventions as a routine part of mammography screening outreach will lead to improved cancer outcomes for Hispanic patients. |
| **80** | Conserve DF, et.al. (80) | 2017 | 7 | Australia, India, United Kingdom, China, South Africa | Vulnerable population | Human immunodeficiency virus | Health | Delivery arrangements | Moderate | MHealth interventions may prove beneficial in reducing the proportion of undiagnosed persons living with HIV, particularly among vulnerable and key populations. However, more rigorous and tailored intervention trials are needed to assess the effectiveness of widespread use. |
| **81** | Morrison J, et.al. (81) | 2014 | 23 | Scotland, United Kingdom, Ireland, Sweden | General population | Infant health | Education Health Social | Delivery arrangements | Critially Low | Interventions with better outcomes and a higher level of evidence combined workshops and educational programmes for both parents and children beginning in early pregnancy and included home visits by specialised staff. More literature reviews focusing on the grey literature are needed to develop a larger evidence base on early childhood interventions |
| **82** | Mosdøl A, et.al. (82) | 2017 | 6 | United States | Minorities (racial or ethnic group) | Healthy lifestyles | Health | Delivery arrangements, Implementation Strategies | High | The available evidence is inadequate for understanding whether mass media interventions targeted toward ethnic minority populations are more effective in changing health behaviours than mass media interventions intended for the population at large. When compared to no intervention, a targeted mass media intervention may increase the number of calls to smoking quit line, but the effect on health behaviours is unclear. These studies could not distinguish the impact of different components, for instance the effect of hearing a message regarding behavioural change, the cultural adaptation to the ethnic minority group, or increase reach to the target group through more appropriate mass media channels. |
| **83** | Beauchamp A, et.al. (83) | 2014 | 14 | United States, Netherlands, France, Germany, Australia | General population | Obesity | Health | Delivery arrangements, Implementation Strategies | Moderate | This review suggests that more structural approaches to obesity prevention are less likely to widen existing inequalities in obesity. If we are to reduce the burden of overweight and obesity among lower socioeconomic groups then all existing and proposed obesity prevention interventions must be measured for their impact on socioeconomic inequalities in health. Those that are more equitable should be prioritized, while those that are not must either not be used, or must be complemented with strategies that will not leave the disadvantaged behind. |
| **84** | Kornet-van DA, et.al. (84) | 2017 | 13 | United States, Australia, Sweden, India | Infant/adolescent population | Obesity | Health | Delivery arrangements, Implementation Strategies | Moderate | The current evidence suggests that involving adolescents in the development and delivering of interventions seems promising. Parents also appear to play an important role in facilitating a healthier lifestyle for their children. For adolescents from disadvantaged backgrounds, we also recommend to use experiential activities as opposed to didactic lessons and to deliver interventions at no or low costs to schools and students. |
| **85** | O'Mara-Eves A, et.al. (85) | 2015 | 131 | United States, United Kingdom, Canada | Vulnerable population | General health | Health | Delivery arrangements, Implementation Strategies, Governance arrangements | Critially Low | There is solid evidence that community engagement interventions have a positive impact on a range of health and psychosocial outcomes, across various conditions. There is insufficient evidence to determine whether one particular model of community engagement is most likely to be more effective than any other |
| **86** | Ruiz-Perez I, et.al. (86) | 2017 | 34 | United States, Europe | Women Minorities (racial or ethnic group) | Human immunodeficiency virus | Health | Delivery arrangements, Implementation Strategies | Critially Low | This study demonstrates the feasibility and effectiveness of developing HIV prevention interventions targeting socioeconomically deprived ethnic minority women. Additional research in this area is necessary in countries outside the United States, and there is also a need to determine the cost-effectiveness of such interventions. |
| **87** | Bastos ML, et.al. (87) | 2018 | 31 | United States | General population | General health | Health | Delivery arrangements | Moderate | The Family Health Strategy expansion was consistently associated with reductions in post-neonatal and child mortality, and less-consistently with reductions in hospitalizations from primary care sensitive conditions. This evidence supports the vital role for primary healthcare in improving health outcomes, and as part of progress towards Universal Health Coverage and the Sustainable Development Goals. |
| **88** | Schaffler J, et.al. (88) | 2017 | 23 | Brazil | Vulnerable population | Chronic diseases | Health | Delivery arrangements | Moderate | Low income and low health literacy are both associated with poorer health outcomes and higher rates of chronic disease. Thus, self-management represents an important healthcare strategy for these populations. Overall, the current review found that effective interventions tended to focus on problem-solving and to a certain extent taking action and resource utilization. A wide range of health-related outcomes were considered, but only empowerment (or self-efficacy) and disease-specific quality of life were found to be positively affected by the interventions. Tailoring did not seem to impact on efficacy. |
| **89** | de Vries DH, et.al. (89) | 2017 | 32 | Kenya, Pakistan, Bangladesh, Brazil, Uganda, India, South Africa, Ghana, Zambia, Benin, Madagascar, Honduras, Burma, Banco Oeste, Sierra Leone | General population | General health | Economic Health Social | Delivery arrangements, Implementation Strategies | Critially Low | Published, peer-reviewed studies evaluating the effectiveness and sustainability of Community or Lay Health Workers (CLHWs) interventions in health programs have not yet adequately tested for the potential of utilizing existing community health roles or social networks for the development of effective and sustainable (retentive) CLHW programs. Community relationships are generally seen as a “black box” represented by an interchangeable CLHW labor unit. This disconnect from community relationships and resources may have led to a systematic and chronic undervaluing of community agency in explanations of programmatic effectiveness and sustainability |
| **90** | Boland VC, et.al. (90) | 2018 | 13 | United States, United Kingdom, New Zealand | General population | General health | Health | Delivery arrangements | Moderate | Few methodologically rigorous studies were identified. Mobile phone text-messaging, computer- and website-delivered quit support showed promise at increasing quit rates among Indigenous, psychiatric and inpatient substance use disorder patients. Further research is needed to address the role technology-based interventions have on overcoming health inequalities to meet the needs of disadvantaged groups. |
| **91** | Butel J, et.al. (91) | 2019 | 8 | United States, Tanzania, Nepal, India | Vulnerable population | Oral health | Health | Delivery arrangements | Critially Low | This review found that interventions that utilize collective efficacy hold the promise to reduce health disparities in communities. The findings also point to the importance of better understanding how collective efficacy works to reduce health disparities. This is especially relevant as the decrease in prevention funding requires more collaborative, grassroots initiatives to affect community change |
| **92** | Nathan, et.al. (92) | 2016 | 17 | The review does not informe | Vulnerable population | Diabetes e hypertension | Health | Delivery arrangements | Low | Decision aids (DAs)in minority populations have been similarly efficacious as DAs in majority populations. However, there is currently a lack of research on DAs in racial, ethnic, sexual, and gender minority populations and a lack of diversity of tools and study participants within existing literature. The lack of DA research in minority populations parallels a general lack of shared decision making in actual clinical practice for minority patients |
| **93** | Oliver-Williams C, et.al. (93) | 2017 | 11 | India, Pakistan, Malawi, Nigeria, Bangladesh, Zambia, Zimbabwe, Kenya | General population | Healthy lifestyles | Telecommunication Health Education | Delivery arrangements | Moderate | Although the potential for mHealth interventions to improve vaccination coverage seems clear, the evidence for such interventions is not. The dearth of studies in countries facing the greatest barriers to immunization impedes the prospects for evidence-based policy and practice in these settings. |
| **94** | Cairns JM, et.al. (94) | 2014 | 14 | United States, Chile, Brazil, Australia, Korea, Germany | Vulnerable population | Obesity | Economic Health | Delivery arrangements, Governance arrangements | High | There is experimental evidence that workplace delivered physical activity interventions have the potential to reduce inequalities in obesity by targeting lower occupational groups. However, overall, the evidence base is small, largely from the USA, and of a low quality. More high-quality, experimental study designs are required |
| **95** | Yuan B, et.al. (95) | 2014 | 22 | Bangladesh, Brazil, China, Colombia, Guinea, India, Indonesia, Nepal, Nigeria, Philippines, South Africa, Tanzania, Uganda, Nigeria, Zambia | Maternal infant | Maternal infant health | Health | Implementation Strategies | Moderate | Studies about the effectiveness of interventions on equity in maternal or child health are limited. The limited evidence showed that the interventions that were effective in reducing inequity included the improvement of health care delivery by outreach methods, using human resources in local areas or provided at the community level nearest to residents and the provision of financial or knowledge support to demand side. |
| **96** | Byrne A, et.al. (96) | 2014 | 34 | Afganistan, Bolivia, Ethiopia, Guatemala, Indonesia, Kenya, Kirguistan, Nepal, Pakistan, Papua New Guinea, Tayikistán | Maternal infant | Maternal infant health | Health Politics | Implementation Strategies, Delivery arrangements | Moderate | The review highlights where known evidence-based strategies have increased the utilisation of reproductive maternal, new-born and child health services in low income mountainous areas. While these are known strategies in public health, in such disadvantaged settings additional supports are required to address both supply and demand barriers |
| **97** | Van Rijn RM, et.al. (97) | 2016 | 16 | The review does not informe | General population | Mental health | Education Occupation Health Social | Delivery arrangements, Implementation Strategies | Moderate | This systematic review shows indications that reemployment programmes based on the place-than-train model have some effect on the quality of life of participants, but the effect size is modest. The current systematic review highlights two important challenges for the future. |
| **98** | Njeru JW, et.al. (98) | 2018 | 54 | United States | Vulnerable population | Diabetes | Health | Delivery arrangements, Implementation Strategies | Moderate | Multiple types of interventions are available for type 2 diabetes mellitus (T2DM) management among patients with limited English proficiency (LEP). Multicomponent interventions delivered face-to-face seem most effective for glycemic control. More research is needed to better understand other aspects of multicomponent interventions that are critical for improving important outcomes for patients with T2DM and LEP. Physician-specific educational tools must be developed to facilitate care of patients with T2DM and LEP. |
| * Publication year of the review  ** Countries of the included studies in the systematic review  *** Effective Practice and Organisation of Care (EPOC) Taxonomy. Cochrane Effective Practice and Organisation of Care. 2015 (99)  **** Tool AMSTAR 2 a critical appraisal tool for systematic reviews that include randomised or non-randomised studies of healthcare interventions, or both (100,101) | | | | | | | | | | |

**Referencias**

1. Araujo M, Moraga C, Chapman E, Barreto J, Illanes E. Intervenciones para mejorar el acceso a los servicios de salud de los pueblos indígenas en las Américas. Rev Panam Salud Pública [Internet]. 2016;40(5):371–81. Available from: http://www.scielosp.org/scielo.php?script=sci_arttext&pid=S1020-49892016001100371&lang=pt%0Ahttp://www.scielosp.org/pdf/rpsp/v40n5/1020-4989-RPSP-40-05-371.pdf

2. Man LC, DiCarlo M, Lambert E, Sifri R, Romney M, Fleisher L, et al. A learning community approach to identifying interventions in health systems to reduce colorectal cancer screening disparities. Prev Med Reports [Internet]. 2018;12(February):227–32. Available from: https://doi.org/10.1016/j.pmedr.2018.10.009

3. Coughlin SS, Smith SA. A review of community-based participatory research studies to promote physical activity among African Americans. J Georg Public Heal Assoc [Internet]. 2016;5(3):220–7. Available from: http://www.ncbi.nlm.nih.gov/pubmed/27034993

4. Rodrigues AL, Ball J, Ski C, Stewart S, Carrington MJ. A systematic review and meta-analysis of primary prevention programmes to improve cardio-metabolic risk in non-urban communities. Prev Med (Baltim) [Internet]. 2016;87:22–34. Available from: http://dx.doi.org/10.1016/j.ypmed.2016.02.011

5. Hamilton K, Tolfree R, Mytton J. A systematic review of active case-finding strategies for tuberculosis in homeless populations. Int J Tuberc Lung Dis [Internet]. 2018 Oct 1;22(10):1135–44. Available from: http://www.ingentaconnect.com/content/10.5588/ijtld.17.0784

6. Lee-Tauler SY, Eun J, Corbett D, Collins PY. A systematic review of interventions to improve initiation of mental health care among racial-ethnic minority groups. Psychiatr Serv. 2018;69(6):628–47.

7. Starbird LE, DiMaina C, Sun CA, Han HR. A Systematic Review of Interventions to Minimize Transportation Barriers Among People with Chronic Diseases. J Community Health [Internet]. 2019;44(2):400–11. Available from: http://dx.doi.org/10.1007/s10900-018-0572-3

8. Melvin CL, Jefferson MS, Rice LJ, Nemeth LS, Wessell AM, Nietert PJ, et al. A systematic review of lifestyle counseling for diverse patients in primary care. Prev Med (Baltim) [Internet]. 2017 Jul;100:67–75. Available from: http://link.springer.com/10.1007/s10903-013-9968-4

9. Pratt CA, Loria CM, Arteaga SS, Nicastro HL, Lopez-Class M, de Jesus JM, et al. A Systematic Review of Obesity Disparities Research. Am J Prev Med [Internet]. 2017;53(1):113–22. Available from: http://dx.doi.org/10.1016/j.amepre.2017.01.041

10. Tovar A, Renzaho AMN, Guerrero AD, Mena N, Ayala GX. A Systematic Review of Obesity Prevention Intervention Studies among Immigrant Populations in the US. Curr Obes Rep. 2014;3(2):206–22.

11. Hillier-Brown FC, Bambra CL, Cairns J-M, Kasim A, Moore HJ, Summerbell CD. A systematic review of the effectiveness of individual, community and societal level interventions at reducing socioeconomic inequalities in obesity amongst children. BMC Public Health [Internet]. 2014 Dec 11;14(1):834. Available from: http://bmcpublichealth.biomedcentral.com/articles/10.1186/1471-2458-14-834

12. Hillier-Brown FC, Bambra CL, Cairns JM, Kasim A, Moore HJ, Summerbell CD. A systematic review of the effectiveness of individual, community and societal-level interventions at reducing socio-economic inequalities in obesity among adults. Int J Obes [Internet]. 2014;38(12):1483–90. Available from: http://dx.doi.org/10.1038/ijo.2014.75

13. Wang L, Zhong B, Vardoulakis S, Zhang F, Pilot E, Li Y, et al. Air quality strategies on public health and health equity in Europe—A systematic review. Int J Environ Res Public Health. 2016;13(12).

14. McGill R, Anwar E, Orton L, Bromley H, Lloyd-Williams F, O’Flaherty M, et al. Are interventions to promote healthy eating equally effective for all? Systematic review of socioeconomic inequalities in impact Health behavior, health promotion and society. BMC Public Health [Internet]. 2015;15(1):1–15. Available from: ???

15. Yadee J, Bangpan M, Thavorn K, Welch V, Tugwell P, Chaiyakunapruk N. Assessing evidence of interventions addressing inequity among migrant populations: A two-stage systematic review. Int J Equity Health. 2019;18(1):1–16.

16. Martinez O, Boles J, Muñoz-Laboy M, Levine EC, Ayamele C, Eisenberg R, et al. Bridging health disparity gaps through the use of medical legal partnerships in patient care: A systematic review. J Law, Med Ethics. 2017;45(2):260–73.

17. Harris J, Springett J, Croot L, Booth A, Campbell F, Thompson J, et al. Can community-based peer support promote health literacy and reduce inequalities? A realist review. Public Heal Res. 2015;3(3):1–192.

18. Lm A, Kl A, Shinn C, Safranek S, Lk K. Community coalition-driven interventions to reduce health disparities among racial and ethnic minority populations. Cochrane Libr. 2015;(6):1–182.

19. Verhagen I, Steunenberg B, De Wit NJ, Ros WJG. Community health worker interventions to improve access to health care services for older adults from ethnic minorities: A systematic review. BMC Health Serv Res. 2014;14(1):1–8.

20. Banna J, Bersamin A. Community involvement in design, implementation and evaluation of nutrition interventions to reduce chronic diseases in indigenous populations in the U.S.: A systematic review. Int J Equity Health. 2018;17(1).

21. Walton-Moss B, Samuel L, Nguyen TH, Commodore-Mensah Y, Hayat MJ, Szanton SL. Community-Based Cardiovascular Health Interventions in Vulnerable Populations. J Cardiovasc Nurs [Internet]. 2014 Jul;29(4):293–307. Available from: https://insights.ovid.com/crossref?an=00005082-201407000-00005

22. Coughlin SS, Smith SA. Community-based participatory research to promote healthy diet and nutrition and prevent and control obesity among African-Americans: A literature review. J Racial Ethn Heal Disparities. 2017;4(2):259–68.

23. Vallury KD, Jones M, Oosterbroek C. Computerized cognitive behavior therapy for anxiety and depression in rural areas:a systematic review. J Med Internet Res. 2015;17(6):e139.

24. Gardner F, Leijten P, Mann J, Landau S, Harris V, Beecham J, et al. Could scale-up of parenting programmes improve child disruptive behaviour and reduce social inequalities? Using individual participant data meta-analysis to establish for whom programmes are effective and cost-effective. Public Heal Res. 2017;5(10):1–144.

25. Dauvrin M, Lorant V. Culturally competent interventions in Type 2 diabetes mellitus management: An equity-oriented literature review. Ethn Heal [Internet]. 2014;19(6):579–600. Available from: http://dx.doi.org/10.1080/13557858.2013.857763

26. Skeie MS, Klock KS. Dental caries prevention strategies among children and adolescents with immigrant-or low socioeconomic backgrounds-do they work? A systematic review. BMC Oral Health. 2018;18(1):1–12.

27. Durand MA, Carpenter L, Dolan H, Bravo P, Mann M, Bunn F, et al. Do interventions designed to support shared decision- making reduce health inequalities? A systematic review and meta-analysis. PLoS One. 2014;9(4).

28. Pottie K, Medu O, Welch V, Dahal GP, Tyndall M, Rader T, et al. Effect of rapid HIV testing on HIV incidence and services in populations at high risk for HIV exposure: An equity-focused systematic review. BMJ Open. 2014;4(12).

29. Batsis JA, Dimilia PR, Seo LM, Fortuna KL, Kennedy MA, Blunt HB, et al. Effectiveness of Ambulatory Telemedicine Care in Older Adults : A Systematic Review. 2019;1–13.

30. Pinzón Flórez CE, Díaz-Quijano DM, Yáñez Álvarez I, Catalina Mesa D. Efectividad de los trabajadores comunitarios en medidas preventivas para salud maternal e infantil en países de bajos y medianos ingresos: revisión sistemática de la literatura. Vol. 31, Revista Salud Uninorte. scieloco; 2015. p. 309–28.

31. Joo JY. Effectiveness of Culturally Tailored Diabetes Interventions for Asian Immigrants to the United States. Diabetes Educ [Internet]. 2014 Sep 14;40(5):605–15. Available from: http://journals.sagepub.com/doi/10.1177/0145721714534994

32. Lacroix JM, Snyder LB, Huedo-medina TB, Johnson BT. Effectiveness of Mass Media Interventions for HIV Prevention , 1986 – 2013 : A Meta-analysis. 2014;66:329–40.

33. Escribà-Agüir V, Rodríguez-Gómez M, Ruiz-Pérez I. Effectiveness of patient-targeted interventions to promote cancer screening among ethnic minorities: A systematic review. Cancer Epidemiol [Internet]. 2016 Oct;44:22–39. Available from: http://dx.doi.org/10.1016/j.canep.2016.07.009

34. Mbuzi V, Fulbrook P, Jessup M. Effectiveness of programs to promote cardiovascular health of Indigenous Australians : a systematic review. 2018;1–17.

35. Reilly R, Evans K, Gomersall J, Gorham G, Peters MDJ, Warren S, et al. Effectiveness, cost effectiveness, acceptability and implementation barriers/enablers of chronic kidney disease management programs for Indigenous people in Australia, New Zealand and Canada: a systematic review of mixed evidence. BMC Health Serv Res [Internet]. 2016 Dec 6;16(1):119. Available from: http://dx.doi.org/10.1186/s12913-016-1363-0

36. Blanchard AK, Prost A, Houweling TAJ. Effects of community health worker interventions on socioeconomic inequities in maternal and newborn health in low-income and middle- income countries : a mixed-methods systematic review. 2019;1–11.

37. Kim K, Choi JS, Choi E, Nieman CL, Joo JH, Lin FR, et al. Effects of Community-Based Health Worker Interventions to Improve Chronic Disease Management and Care Among Vulnerable Populations : A Systematic Review. 2016;106(4):3–28.

38. Hahn RA, Rammohan V, Truman BI, Milstein B, Johnson RL, Muntañer C. Effects of Full-Day Kindergarten on the Long-Term Health Prospects of Children in Low-Income and Racial/Ethnic-Minority Populations. Am J Prev Med [Internet]. 2014;46(3):312–23. Available from: http://dx.doi.org/10.1016/j.amepre.2013.12.003

39. Speyer R, Denman D, Wilkes-Gillan S, Chen Y, Bogaardt H, Kim J, et al. Effects of telehealth by allied health professionals and nurses in rural and remote areas: A systematic review and meta-analysis. J Rehabil Med [Internet]. 2018;50(3):225–35. Available from: https://www.medicaljournals.se/jrm/content/abstract/10.2340/16501977-2297

40. Antoniades J, Mazza D, Brijnath B. Efficacy of depression treatments for immigrant patients : results from a systematic review. 2014;14(1):1–12.

41. Parker S, Prince A, Thomas L, Song H, Milosevic D, Harris MF. Electronic, mobile and telehealth tools for vulnerable patients with chronic disease: a systematic review and realist synthesis. BMJ Open [Internet]. 2018 Aug 29;8(8):e019192. Available from: http://bmjopen.bmj.com/lookup/doi/10.1136/bmjopen-2017-019192

42. Abbott LS, Elliott LT. Eliminating Health Disparities through Action on the Social Determinants of Health : A Systematic Review of Home Visiting in the United States , 2005 – 2015. 2020;34(1):2–30.

43. Brown T, Platt S, Amos A. Equity impact of European individual-level smoking cessation interventions to reduce smoking in adults : a systematic review. 2013;551–6.

44. Brown T, Platt S, Amos A. Equity impact of interventions and policies to reduce smoking in youth : systematic review. 2014;1–8.

45. Brown T, Platt S, Amos A. Equity impact of population-level interventions and policies to reduce smoking in adults : A systematic review ଝ. Drug Alcohol Depend [Internet]. 2014;138:7–16. Available from: http://dx.doi.org/10.1016/j.drugalcdep.2014.03.001

46. Attwood S, Sluijs E Van, Sutton S. Exploring equity in primary-care-based physical activity interventions using PROGRESS-Plus : a systematic review and evidence synthesis. Int J Behav Nutr Phys Act [Internet]. 2016; Available from: http://dx.doi.org/10.1186/s12966-016-0384-8

47. Cyril S, Smith BJ, Possamai-Inesedy A, Renzaho AMN. Exploring the role of community engagement in improving the health of disadvantaged populations: a systematic review. Glob Health Action [Internet]. 2015 Dec 18;8(1):29842. Available from: https://www.medicaljournals.se/jrm/content/abstract/10.2340/16501977-2297

48. Ashman AM, Brown LJ, Collins CE, Rollo ME, Rae KM. Factors Associated with Effective Nutrition Interventions for Pregnant Indigenous Women: A Systematic Review. J Acad Nutr Diet [Internet]. 2017 Aug;117(8):1222-1253.e2. Available from: http://dx.doi.org/10.1016/j.jand.2017.03.012

49. Kristjansson E, Dk F, Liberato S, M BJ, Welch V, Batal M, et al. Food supplementation for improving the physical and psychosocial health of socio-economically disadvantaged children aged three months to five years ( Review ). 2015;(3).

50. Valdez LA, Flores M, Ruiz J, Oren E, Carvajal S, David O. Gender and Cultural Adaptations for Diversity : A Systematic Review of Alcohol and Substance Abuse Interventions for Latino Males Gender and Cultural Adaptations for Diversity : A Systematic Review of Alcohol. Subst Use Misuse [Internet]. 2018;53(10):1608–23. Available from: https://doi.org/10.1080/10826084.2017.1417999

51. Fernández-Gutiérrez M, Bas-Sarmiento P, Albar-Marín MJ, Paloma-Castro O, Romero-Sánchez JM. Health literacy interventions for immigrant populations: a systematic review. Int Nurs Rev. 2018;65(1):54–64.

52. Kim SH, Lee RNA. Health-Literacy-Sensitive Diabetes Self-Management Interventions : A Systematic Review and Meta-Analysis. 2016;324–33.

53. Mojica CM, Parra-Medina D, Vernon S. Interventions promoting colorectal cancer screening among Latino men: A systematic review. Prev Chronic Dis. 2018;15(3):1–7.

54. Mayén AL, De Mestral C, Zamora G, Paccaud F, Marques-Vidal P, Bovet P, et al. Interventions promoting healthy eating as a tool for reducing social inequalities in diet in low- and middle-income countries: A systematic review. Int J Equity Health. 2016;15(1):1–10.

55. Robinson JL, Narasimhan M, Amin A, Morse S, Beres LK, Yeh PT, et al. Interventions to address unequal gender and power relations and improve self-efficacy and empowerment for sexual and reproductive health decision-making for women living with HIV: A systematic review. PLoS One. 2017;12(8):1–19.

56. Barley EA, Borschmann RD, Walters P, Tylee A. Interventions to encourage uptake of cancer screening for people with severe mental illness. Cochrane Database Syst Rev [Internet]. 2016 Sep 26;7(9). Available from: http://doi.wiley.com/10.1002/14651858.CD009641.pub3

57. Ramke J, Petkovic J, Welch V, Blignault I, Gilbert C, Blanchet K, et al. Interventions to improve access to cataract surgical services and their impact on equity in low- and middle-income countries. Cochrane Database Syst Rev [Internet]. 2017 Nov 9;(9). Available from: http://doi.wiley.com/10.1002/14651858.CD011307.pub2

58. Health Quality Ontario. Interventions to Improve Access to Primary Care for People Who Are Homeless: A Systematic Review. Ont Health Technol Assess Ser [Internet]. 2016;16(9):1–50. Available from: http://www.hqontario.ca/Evidence-to-Improve-Care/Journal-Ontario-Health-Technology-Assessment- Series

59. Clifford A, Mccalman J, Bainbridge R, Tsey K. Interventions to improve cultural competency in health care for Indigenous peoples of Australia, New Zealand, Canada and the USA: A systematic review. Int J Qual Heal Care. 2015;27(2):89–98.

60. Mercer GD, Lyons P, Bassett K. Interventions to improve gender equity in eye care in low-middle income countries: A systematic review. Ophthalmic Epidemiol [Internet]. 2019;26(3):189–99. Available from: https://doi.org/10.1080/09286586.2019.1574839

61. Rees I, Jones D, Chen H, Macleod U. Interventions to improve the uptake of cervical cancer screening among lower socioeconomic groups: A systematic review. Prev Med (Baltim). 2018;111(March):323–35.

62. Bhui KS, Aslam RW, Palinski A, McCabe R, Johnson MRD, Weich S, et al. Interventions to improve therapeutic communications between Black and minority ethnic patients and professionals in psychiatric services: Systematic review. Br J Psychiatry. 2015;207(2):95–103.

63. Hu D, Juarez DT air., Yeboah M, Castillo TP. Interventions to increase medication adherence in African-American and Latino populations: a literature review. Hawaii J Med Public Health. 2014;73(1):11–8.

64. Crocker-Buque T, Edelstein M, Mounier-Jack S. Interventions to reduce inequalities in vaccine uptake in children and adolescents aged < 19 years: A systematic review. J Epidemiol Community Health. 2016;71(1):87–97.

65. Raison H, Harris R V. Interventions to reduce socio-economic inequalities in dental service utilisation - a systematic review. Community Dent Health [Internet]. 2019 Feb 25;36(1):39–45. Available from: http://www.ncbi.nlm.nih.gov/pubmed/30779498

66. Moore KL. Mental Health Service Engagement Among Underserved Minority Adolescents and Young Adults: a Systematic Review. J Racial Ethn Heal Disparities. 2018;5(5):1063–76.

67. Anderson-Lewis C, Darville G, Mercado RE, Howell S, Di Maggio S. mHealth technology use and implications in historically underserved and minority populations in the united states: Systematic literature review. JMIR mHealth uHealth. 2018;6(6).

68. Santos Salas A, Fuentes Contreras J, Armijo-Olivo S, Saltaji H, Watanabe S, Chambers T, et al. Non-pharmacological cancer pain interventions in populations with social disparities: a systematic review and meta-analysis. Support Care Cancer. 2016;24(2):985–1000.

69. Goudet SM, Griffiths PL, Bogin BA, Madise NJ. Nutritional interventions for preventing stunting in children (0 to 5 years) living in urban slums in low and middle-income countries (LMIC). Cochrane Database Syst Rev [Internet]. 2015 May 12;(5). Available from: http://doi.wiley.com/10.1002/14651858.CD011695

70. Tsai C, Blinkhorn A, Irving M. Oral Health Programmes in Indigenous Communities Worldwide—Lessons learned from the field: A qualitative systematic review. Community Dent Oral Epidemiol. 2017;45(5):389–97.

71. Khanassov V, Pluye P, Descoteaux S, Haggerty JL, Russell G, Gunn J, et al. Organizational interventions improving access to community-based primary health care for vulnerable populations: a scoping review. Int J Equity Health. 2016;15(1):1–34.

72. McCurley JL, Crawford MA, Gallo LC. Prevention of Type 2 Diabetes in U.S. Hispanic Youth: A Systematic Review of Lifestyle Interventions. Am J Prev Med [Internet]. 2017 Oct;53(4):519–32. Available from: https://linkinghub.elsevier.com/retrieve/pii/S074937971730291X

73. Mitchell SA, Kneipp SM, Giscombe CW. Social Factors Related to Smoking among Rural, Low-Income Women: Findings from a Systematic Review. Public Health Nurs. 2016;33(3):214–23.

74. Moore GF, Littlecott HJ, Turley R, Waters E, Murphy S. Socioeconomic gradients in the effects of universal school-based health behaviour interventions: A systematic review of intervention studies. BMC Public Health. 2015;15(1).

75. Heller C, Balls-Berry JE, Nery JD, Erwin PJ, Littleton D, Kim M, et al. Strategies addressing barriers to clinical trial enrollment of underrepresented populations: A systematic review. Contemp Clin Trials [Internet]. 2014;39(2):169–82. Available from: http://dx.doi.org/10.1016/j.cct.2014.08.004

76. Jia L, Yuan B, Huang F, Lu Y, Garner P, Meng Q. Experiencia, intersubjetividad y existencia. Hacia una teoría-práctica de la Etnografía. Run Arch para las Ciencias del Hombre. 1994;21(1):347–80.

77. Domingo JLB, Gavero G, Braun KL. Strategies to increase Filipino American participation in cardiovascular health promotion: A systematic review. Prev Chronic Dis. 2018;15(5):1–11.

78. Sharma BB, Jones L, Loxton DJ, Booth D, Smith R. Systematic review of community participation interventions to improve maternal health outcomes in rural South Asia. BMC Pregnancy Childbirth. 2018;18(1):1–16.

79. Luque JS, Logan A, Soulen G, Armeson KE, Garrett DM, Davila CB, et al. Systematic Review of Mammography Screening Educational Interventions for Hispanic Women in the United States. J Cancer Educ. 2019;34(3):412–22.

80. Conserve DF, Jennings L, Aguiar C, Shin G, Handler L, Maman S. Systematic review of mobile health behavioural interventions to improve uptake of HIV testing for vulnerable and key populations. J Telemed Telecare. 2016;23(2):347–59.

81. Morrison J, Pikhart H, Ruiz M, Goldblatt P. Systematic review of parenting interventions in European countries aiming to reduce social inequalities in children’s health and development. BMC Public Health. 2014;14(1).

82. Mosdøl A, Lidal IB, Straumann GH, Vist GE. Targeted mass media interventions promoting healthy behaviours to reduce risk of non-communicable diseases in adult, ethnic minorities. In: Mosdøl A, editor. Cochrane Database of Systematic Reviews [Internet]. Chichester, UK: John Wiley & Sons, Ltd; 2015. p. 200. Available from: http://doi.wiley.com/10.1002/14651858.CD011683

83. Beauchamp A, Backholer K, Magliano D, Peeters A. The effect of obesity prevention interventions according to socioeconomic position: A systematic review. Obes Rev. 2014;15(7):541–54.

84. Kornet-van DA, Altenburg TM, van Randeraad-van der Zee CH, Chinapaw MJM. The effectiveness and promising strategies of obesity prevention and treatment programmes among adolescents from disadvantaged backgrounds: a systematic review. Obes Rev. 2017;18(5):581–93.

85. O’Mara-Eves A, Brunton G, Oliver S, Kavanagh J, Jamal F, Thomas J. The effectiveness of community engagement in public health interventions for disadvantaged groups: A meta-analysis. BMC Public Health. 2015;15(1):1–23.

86. Ruiz-Perez I, Murphy M, Pastor-Moreno G, Rojas-García A, Rodríguez-Barranco M. The effectiveness of HIV prevention interventions in socioeconomically disadvantaged ethnic minority women: A systematic review and meta-analysis. Am J Public Health. 2017;107(12):e13–21.

87. Bastos ML, Menzies D, Hone T, Dehghani K, Trajman A. The impact of the Brazilian family health on selected primary care sensitive conditions: A systematic review. PLoS One. 2017;12(8):1–14.

88. Schaffler J, Leung K, Tremblay S, Merdsoy L, Belzile E, Lambrou A, et al. The Effectiveness of Self-Management Interventions for Individuals with Low Health Literacy and/or Low Income: A Descriptive Systematic Review. J Gen Intern Med. 2018;33(4):510–23.

89. De Vries DH, Pool R. The influence of community health resources on effectiveness and sustainability of community and lay health worker programs in lower-income countries: A systematic review. PLoS One. 2017;12(1):1–28.

90. Boland VC, Stockings EA, Mattick RP, McRobbie H, Brown J, Courtney RJ. The Methodological Quality and Effectiveness of Technology-Based Smoking Cessation Interventions for Disadvantaged Groups: A Systematic Review and Meta-analysis. Nicotine Tob Res. 2018;20(3):276–85.

91. Butel J, Braun KL. The Role of Collective Efficacy in Reducing Health Disparities: A Systematic Review. Fam Community Heal. 2019;42(1):8–19.

92. Nathan AG, Marshall IM, Cooper JM, Huang ES. Use of Decision Aids with Minority Patients: a Systematic Review. J Gen Intern Med. 2016;31(6):663–76.

93. Oliver-Williams C, Brown E, Devereux S, Fairhead C, Holeman I. Using Mobile Phones to Improve Vaccination Uptake in 21 Low- and Middle-Income Countries: Systematic Review. JMIR mHealth uHealth. 2017;5(10):e148.

94. Cairns JM, Bambra C, Hillier-Brown FC, Moore HJ, Summerbell CD. Weighing up the evidence: A systematic review of the effectiveness of workplace interventions to tackle socio-economic inequalities in obesity. J Public Heal (United Kingdom). 2015;37(4):659–70.

95. Yuan B, Målqvist M, Trygg N, Qian X, Ng N, Thomsen S. What interventions are effective on reducing inequalities in maternal and child health in low- and middle-income settings? A systematic review. BMC Public Health. 2014;14(1).

96. Byrne A, Hodge A, Jimenez-Soto E, Morgan A. What works? Strategies to increase reproductive, maternal and child health in difficult to access mountainous locations: A systematic literature review. PLoS One. 2014;9(2).

97. Van Rijn RM, Carlier BE, Schuring M, Burdorf A. Work as treatment? the effectiveness of re-employment programmes for unemployed persons with severe mental health problems on health and quality of life: A systematic review and meta-analysis. Occup Environ Med. 2016;73(4):275–9.

98. Njeru JW, Wieland ML, Kwete G, Tan EM, Breitkopf CR, Agunwamba AA, et al. Diabetes Mellitus Management Among Patients with Limited English Proficiency: A Systematic Review and Meta-Analysis. J Gen Intern Med. 2018;33(4):524–32.

99. Effective Practice and Organisation of Care (EPOC). EPOC Taxonomy [Internet]. Cochrane Effective Practice and Organisation of Care. 2015 [cited 2019 Oct 10]. Available from: https://epoc.cochrane.org/epoc-taxonomy

100. Shea BJ, Reeves BC, Wells G, Thuku M, Hamel C, Moran J, et al. AMSTAR 2: a critical appraisal tool for systematic reviews that include randomised or non-randomised studies of healthcare interventions, or both. BMJ [Internet]. 2017 Sep 21;j4008. Available from: http://www.bmj.com/lookup/doi/10.1136/bmj.j4008

101. Ciapponi A. AMSTAR-2: herramienta de evaluación crítica de revisiones sistemáticas de estudios de intervenciones de salud. Evidencia [Internet]. 2017;21(1):4–13. Available from: http://www.evidencia.org.ar/index.php/Evidencia/article/view/3298/1581
